# Interpretable Machine Learning Identifies an Emergent Absence Seizure Mechanism

**DOI:** 10.1101/2025.09.23.678032

**Published:** 2026-09-16

**Authors:** Jacob M. Hull, Nicholas Denomme, Surya Ganguli, John R. Huguenard

**Affiliations:** 1. Department of Neurology and Neurological Sciences, Stanford University, Stanford, CA, USA; 2. Department of Psychiatry, Stanford University, Stanford CA, USA; 3. Department of Applied Physics, Stanford University, Stanford CA USA

## Abstract

Absence seizures are widespread spike-and-wave oscillations disrupting consciousness. Several consciousness frameworks emphasize intercortical/thalamocortical feedback dynamics, but we lack explicit dynamical mechanisms for how seizures disrupt these circuits. Using interpretable machine learning, we derived dynamical equations directly from seizure electrocorticogram data, reproducing seconds-long multi-regional local field potentials with precision matching inter-mouse variability. The model contained a low-dimensional chaotic seizure attractor emerging from between-region synchronization at preferred phase-lags. Unit recordings revealed corresponding spiking synchrony at single-neuron resolution, linking somatosensory and motor cortex with posterior thalamic nucleus (PO). Model coupling terms predicted tonic and burst firing spatiotemporal organization across regions and guided multisite-optogenetic stimulations. These stimulations showed PO controls corticocortical connectivity gain and that motor/somatosensory corticocortical functional connectivity varies at predicted phase-lags to drive seizures. Our results define absence seizures as dynamics confined to a chaotic attractor within a circuit implicated in anesthetic unconsciousness, linking distributed network chaos to loss of consciousness.

## Graphical Abstract

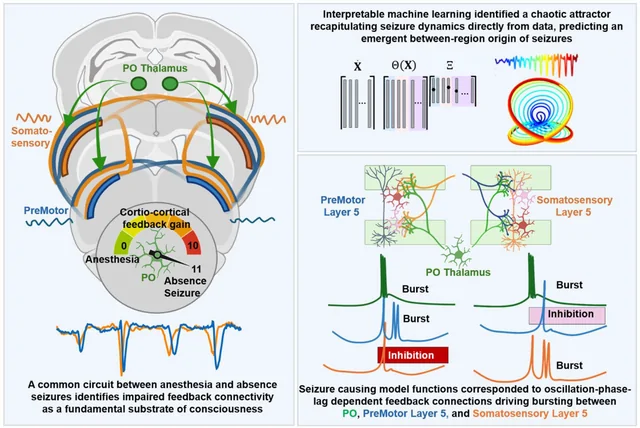

## Introduction

Absence seizures are generalized non-convulsive seizures characterized by impaired consciousness and widespread bilaterally symmetric 3 Hz spike-and-wave oscillations (SWD) on EEG. SWDs are multicomponent sequences of negative spikes accompanying lower frequency positive waves. Their onset is generalized across bilateral frontal/parietal regions, implying multi-regional mechanisms for their generation or rapid spread between regions. The cortical focus theory in rat absence models posits a single somatosensory cortical focus that rapidly spreads.^1^ A core feature of this theory is that somatosensory face/whisker cortical hyperexcitability drives initiation of each seizure, which then rapidly recruits other thalamocortical networks leading to near global cortical synchrony.^2^ Recent in vivo multielectrode and fMRI evidence however suggests little or no somatosensory cortical hyperexcitability in the transition to seizure leading to increased interest in how oscillatory firing and synchrony are coordinated.^3–6^ Absence seizure mechanisms involving hyperexcitability and oscillatory activity may however be interdependent. For example, focal cortical activation can recruit thalamic reticular neurons, inhibit thalamocortical cells and trigger rebound firing that completes an oscillatory cortico-thalamic feedback loop.^7–11^ Consistent with this mechanism, in vitro stimulation of cortical fibers projecting to the thalamus in a pattern mimicking high-frequency cortical bursting, transforms spindle-like (6–10 Hz) thalamic oscillations into hypersynchronous, SWD-like (3–4 Hz) thalamic bursts.^7, 8^ Acting in the opposite direction, thalamic stimulations in cat that induce spindle oscillations can be converted to SWDs when cortical excitability is increased with GABA_A_ blocker penicillin. This conversion from spindle to SWD oscillations occurs through progressive amplification of every second spindle peak, suggesting SWDs emerge from an underlying spindle-like cortico-thalamic oscillation at twice the SWD frequency.^12^

Long-range reciprocal feedback connections, can in principle lead to population level oscillations and synchrony, thereby coordinating sensation and action, and may support consciousness through cortical and subcortical mechanisms.^13–15^ Cortico-thalamic oscillations may be nonlinear, multi-regional, and high dimensional, suggesting the need for a quantitative framework. One framework for understanding large neuronal population dynamics is the concept of attractor states in which system dynamics are confined to a subset of neuronal activity patterns.^16^ Attractors have been identified in many circuits including those involving navigation, motor planning, perceptual decision making, and epilepsy.^17–23^ Such mechanisms may account for the recurrent and stereotyped activity patterns underlying absence epilepsy. While a dynamical systems perspective has greatly expanded our conception of seizure mechanisms beyond excitatory/inhibitory imbalance^23^, a central challenge is discovering how such abstract dynamics arise from specific cellular substrates. Here we combined recent advances in system identification via machine learning, high-density recording, and multisite optogenetic stimulation to systematically investigate the dynamics of absence seizure generation. We used interpretable machine learning to identify a highly accurate system of dynamical equations directly from seizure local field potential (LFP) data. This system accurately simulates seizures to within the high precision of inter-mouse variability. These equations gave rise to a chaotic attractor, where between-region oscillation coupling and phase-lags, rather than a region-specific deficit, drove absence seizures. High density multielectrode recordings and multisite optogenetics then allowed us to identify that this between-region phase-lag dependent seizure drive corresponded to the relative timing of burst firing in layer 5 pyramidal neurons of the motor and somatosensory systems and the amplification of cortico-cortical connectivity gain via higher order thalamic nucleus PO. A detailed summary of our results is given in the discussion section, which may be helpful to read before the results.

## Results

### Absence seizures are stereotyped by sensory-motor system relationships but not trajectories in time

**Figure 1.**
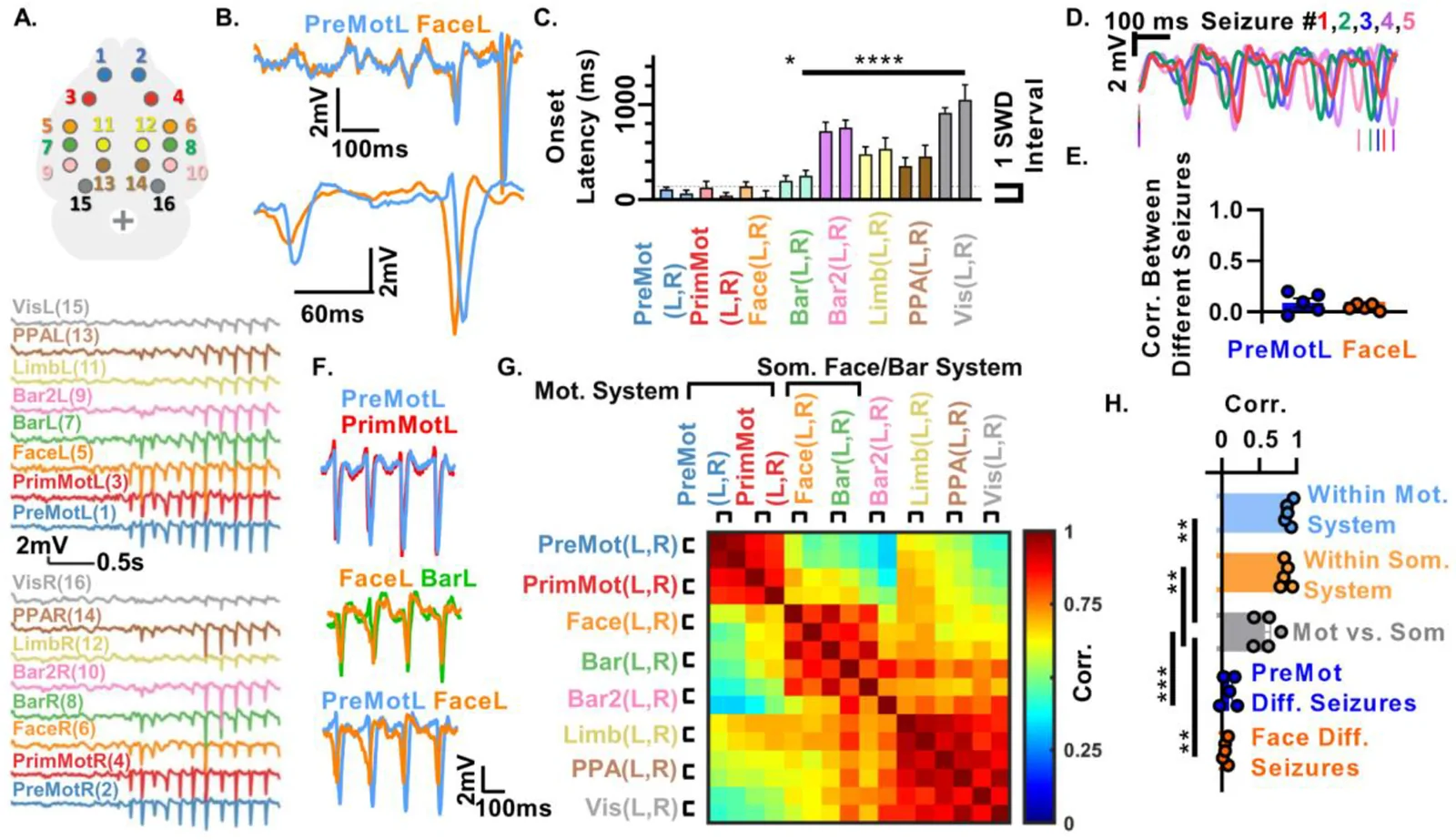
Between-region relationships describe absence seizure dynamics better than temporal trajectories (A) Recording location schematic (top) and example Ecog traces (bottom). (B) Example spike-and-wave discharge (SWD) onset in premotor left (PreMotL) and somatosensory FaceL (FaceL) regions, expanded view in bottom. (C) Median onset latency ± 95% CI relative to the earliest onset site of each seizure (*N* = 5 mice, 30 seizures per mouse). (D) Example traces of five seizures from PreMotL in one mouse aligned to the peak of the first SWD-spike crossing (< −1000 mV); vertical lines mark SWD-spikes at first and sixth crossings. (E) Correlation between seizures within the same animal aligned as in (G): PreMotL 0.09 ± 0.04, FaceL 0.05 ± 0.01 AU. (F) Example motor (PreMotL, PrimMotL) and somatosensory (FaceL, BarL) system waveforms are similar within motor (top) and somatosensory (middle) systems but not between systems (bottom). (G) Average correlation between all region combinations (*N* = 5 mice, 5 seizures per mouse). (H) Within-system correlations: Mot 0.89 ± 0.02, Som 0.85 ± 0.03 vs between-system 0.58 ± 0.07 AU vs between different seizures from recorded from the same region of the same animal from E. Statistical analyses were performed by Cox proportional hazards model (C) and standard or Welch’s two-tailed Student’s *t* test (H); *N* = 5 mice. Data represent mean ± SEM except in C where data are median and 95% CI.

We first aimed to uncover the dynamical laws governing absence seizures by examining seizures in a well-characterized mouse model (med mice, hemizygous for *Scn8a*, encoding Nav1.6).^24^ We developed a high-fidelity electrocorticogram (Ecog) approach with electrodes over 16 cortical regions (Fig. 1A). Individual spikes of a SWD complex in mice span ~133 ms (7.5 Hz), with additional rhythms shaping patterns within and between SWDs (Fig. 1B). Seizure power and onset times were quantified using our “seizure score” metric (Fig. S1 A–D). Median seizure onset times showed no detectable latency differences among premotor left/right (PreMotL/R), primary motor (PrimMotL/R) and somatosensory face (FaceL/R) cortices- all with onset within a single SWD interval (Fig. 1C).^25^ The somatosensory barrel cortex (BarL, BarR) onset was modestly delayed and greater delays were observed in other regions.

We next examined the temporal trajectories of seizures to identify regularities that can be used to understand seizure dynamics. Comparison of trajectories from the same region of the same mouse revealed that different individual seizures quickly diverge from each other in time, with SWD-spikes spanning the full SWD cycle within 1s (Fig. 1D) and with negligible signal correlation between different seizures (Fig. 1E). In contrast, relationships between regions were robustly maintained during seizures. Among early-onset areas, PreMotL and PrimMotL of the motor system showed highly similar SWD waveforms (Fig. 1F, top), as did FaceL and BarL of the somatosensory system (Fig. 1F, middle), while motor vs. somatosensory system signals were more distinct (Fig. 1F, bottom). We quantified similarity using pairwise correlations over the first 3 s of seizure across all region combinations (Fig. 1G). Motor and face/barrel somatosensory systems showed strong, nearly identical correlations within the respective systems and weaker but still strong between-system correlations (Fig. 1H). Comparing these between-system correlations to the between-different-seizure correlations of the same region of the same mouse (Fig. 1E) revealed that between-region relationships far better describe seizure dynamics than temporal trajectories (Fig. 1H).

Overall, these results revealed an early motor and somatosensory seizure network, with highly stereotyped signals within and between these systems, but with rapidly diverging dynamics in time between different seizures.

### Projection onto a toroidal coordinate system revealed highly constrained phase and amplitude dynamics during absence seizures

**Figure 2.**
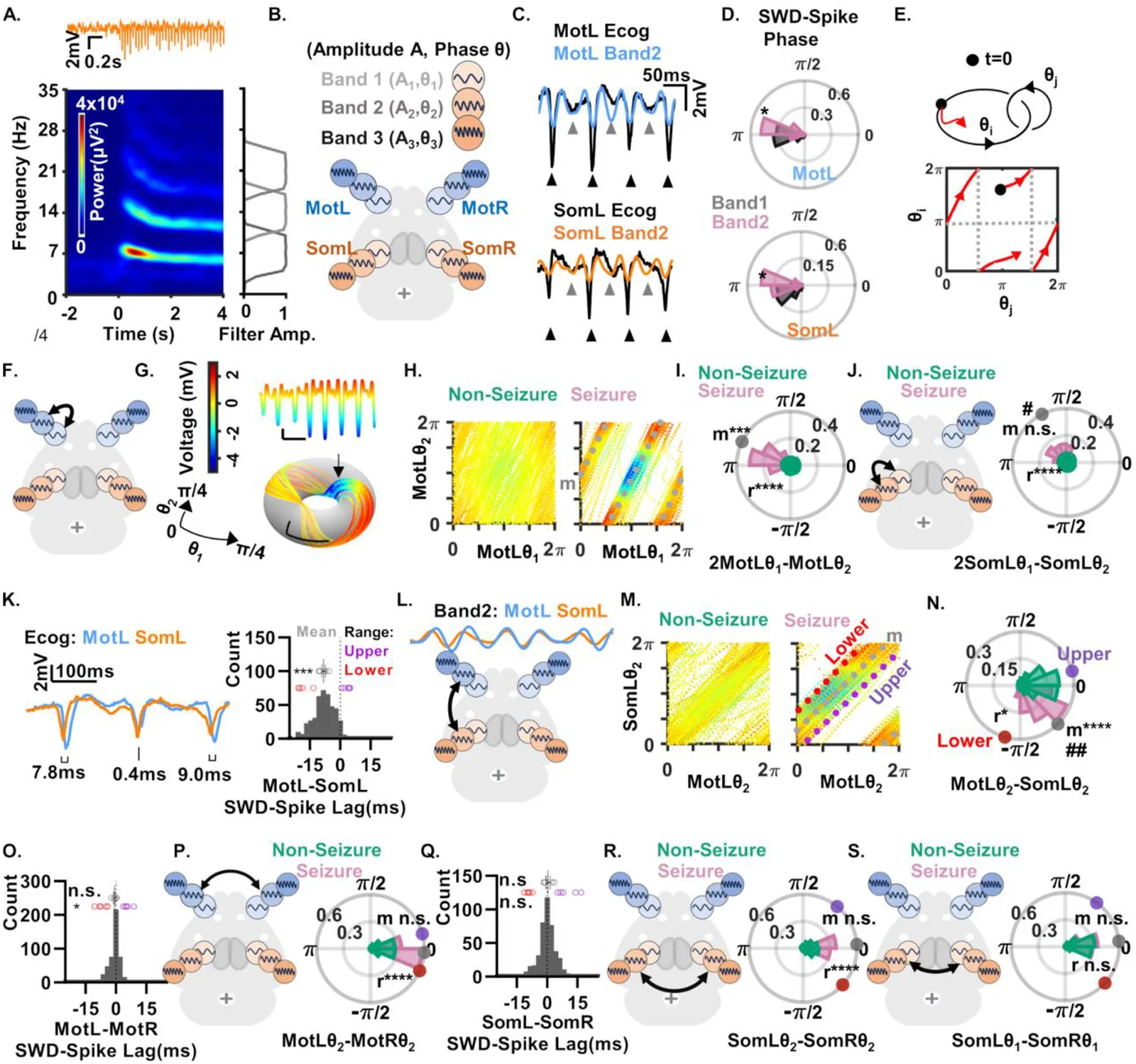
A toroidal state space defines absence seizures (A and B) Average spectrogram of FaceL seizures (A, bottom-left) aligned to an example FaceL seizure (A, top) and corresponding band filters (A, right). Schematic (B) of Mot (L,R) and Som (L,R) signals composed of three harmonics with amplitudes Aᵢ and phases θ_i_. (C) Example traces of full spectrum SWDs (black) and band 2 (blue, orange) in MotL (top) and SomL (bottom). Black arrows indicate negative SWD-spike troughs aligned to band 2 troughs (π rad). Gray arrows indicate Ecog troughs between SWD-spikes aligned with band 2 troughs. (D) Circular histograms of phase of SWD-spikes in bands 1 and 2. Asterisks indicate higher circular standard deviation (more variability) for band 1 than band 2, paired t-test on per mouse circular standard deviation. See Fig. S2A. (E) Toroidal coordinate system (top) and unwrapped representation with periodic boundary wrapping (bottom) illustrating an example phase trajectory (red) and phase wrapping at edges (dashed lines). (F and G) Schematic (F) of the MotL band 1 and 2 interaction. Experimental trajectories (G, bottom) on the toroidal manifold of bands 1 and 2 for MotL color denotes summed amplitudes of the three bands (G, top). Arrow indicates tight cluster of all SWD-spikes. Contrast to dispersed spikes in time in Fig. 1D. (H) MotL band 1:2 unfolded torus in nonseizure (left) and seizure (right) states from 5 seizures. Color as in G. Gray line indicates slope 2 seizure trajectory at phase-lag m. Contrast SWD-spike (blue region) regularity with Fig. 1D. (I) Phase-difference histogram as in H from all mice showing a phase-lag shift m and increased coherence r from the non-seizure to seizure state, paired t-tests. Gray circle indicates mean phase-lag m. (J) SomL band 1:2 interaction schematic (left) and phase histogram (right) showing increased coherence r from the non-seizure to seizure state, paired t-test. Seizure mean phase-lag m (gray circle) differs from MotL (I) # p<0.05, t-test n=5 mean phase-lags, 1 per animal. (K) Overlaid example MotL and SomL Ecog traces with variable SWD-spike timing delays (left). Histogram (right) of MotL-SomL SWD-spike timing delays. Gray circles indicate individual mouse time-lag averages (−8.2 ms, one-sample t-test vs 0), purple (upper: 3.3 ms) and red (lower: −19.6 ms) bounds of 95% range of SWD-spike time-lags. (L) MotL:SomL band 2 interaction schematic (bottom) and example band 2 traces (top) from K. (M) MotL:SomL band 2 torus, color as in H. Dashed lines indicate lower (~−π/2 rad, red) and upper (~+0, purple) phase-lag bounds containing 95% of phase-lags and mean phase-lag m (~−π/4 rad, gray). (N) Phase-difference histogram, showing shifted mean phase-lag m and increased coherence r in the seizure vs non-seizure state, paired t-tests. Dots indicate mean phase-lag (gray) and 95% range of phase-lag distribution respectively as in M, right. Phase-lag differed from 0 in the seizure state (#, one-sample t-test vs 0). Note similarity with non-zero SWD-spike time-lags in K. (O-R) SWD-spike time-lags (O and Q) showing SWD-spike time-lags ~0 ms for MotL-MotR (n.s. one-sample t-test vs 0) and SomL-SomR (n.s. one-sample t-test vs 0). Gray, purple, red circles as in K. 95% range reduced in P relative to K, t-test. Corresponding band interaction schematics and phase-difference histograms (P and R) showing ~0 mean phase-lags m, n.s. one-sample t-test vs 0. There was increased coherence r but not phase-lag m in the seizure vs non-seizure state, paired t-test. Note similarity between 0 rad phase-lags to 0 ms time-lags in O and Q. (S) Schematic SomL/SomR band 1 interaction (left) and corresponding phase-difference histogram (right) showing no difference in phase-lag m or coherence r between seizure and non-seizure, paired t-tests. Histograms contain observations from all mice, N=5 mice, 5 seizures/mouse. Statistical comparisons were performed with values computed per mouse from 5 seizures/mouse.

Fig. 1D and E suggest significant oscillatory phase drift as each seizure unfolds, while Fig. 1F–H suggests waveforms are stereotyped within-systems and that pairs of regions maintain robust phase locking during seizures, especially within and between somatosensory and motor systems. These results motivated a switch from time-voltage coordinates to coordinates that directly represent within- and between-region oscillatory phase-lags and dynamics. Hereafter, we refer to PreMot Ecog LFP signals as Mot and somatosensory face as Som unless otherwise specified. To gain a clear geometric view of within- and between-regional SWD oscillation dynamics, we utilized the dominant frequency bands in the SWD spectrogram. The SomL spectrogram (Fig. 2A) revealed a fundamental frequency in a ~7.5 Hz band (band 1) that slightly decays over time, accompanied by two substantial harmonics at 15 Hz and 22.5 Hz (bands 2 and 3). This 3-band pattern was seen across electrodes (PreMotL, FaceL, Visual L; Fig. S1A). We constructed three filters (Fig. 2A, right) to extract phases and amplitudes of these oscillations via the Hilbert transform (schematic, Fig. 2B).

While the fundamental SWD frequency is ~7.5 Hz, the timing of the SWD-spike (Fig. 2C, black arrows) aligned more strongly with the trough of band 2 than band 1. The trough of band 2 is at π rad by definition and the SWD-spike occurred near the trough in both MotL (2.97 ± 0.02 rad) and SomL (3.10 ± 0.04 rad). To quantify how reliably SWD-spikes are reflected by the phase of the different bands, we compared the circular standard deviation of the phase where SWD-spikes occurred. Standard deviation was 43% and 34% greater (i.e. more variable) for band 1 than 2 in SomL and MotL respectively, indicating tighter locking of SWD-spike timing to band 2 phase over band 1 phase (Fig. 2D, Fig. S2A). Similarly, band 3 standard deviation was 37% greater than band 2 in MotL, indicating tighter locking of SWD-spike to band 2 than band 3, while SomL band 2 and 3 were not different (Fig. S2A). The unfiltered Ecog trace (Fig. 2C black trace) also exhibited an additional peak and trough (gray arrows) between the SWD-spikes which also aligned with band 2 peaks and troughs (Fig. 2C blue and orange traces, gray arrows).

The substantial association of SWD-spike timing across bands suggests understanding both within- and between-region oscillation phase relationships is necessary to understand seizure dynamics and their origin. A standard method to examine how two oscillations evolve relative to each other, is to observe their joint dynamics on a phase torus. Fig. 2E shows a schematic of an arbitrary phase trajectory (red) on a torus defined by two oscillation phases, θ_i_ and θ_j_, where the phase torus is unfolded into a square with a periodic wraparound in both directions. Mapping MotL voltages onto the MotL band 1 phase versus band 2 phase torus (connection schematic Fig. 2F), revealed that SWD occurrences are confined to a specific region of the phase torus across many SWD cycles. For example, the dark blue SWD-spike troughs Fig. 2G, top, occur primarily at a specific point on the torus (Fig. 2G, bottom).

More generally, confinement of the entire trajectory along a limited region on the phase torus (Fig. 2G) suggests the presence of a dynamic attractor. To anticipate the quantitative interpretation of seizure dynamics in the toroidal coordinate system, we outline several theoretical possibilities and how to quantitatively detect them. For two oscillator phases (θ_i_, θ_j_), identical oscillation frequencies produce a slope-1 trajectory that repeats indefinitely, while differing frequencies cause diverging trajectories, filling the torus for irrational slopes (i.e. irrational oscillation frequency ratios, Fig. S2B). This stability of phase relationships is captured by phase-difference histograms, quantified by the coherence r ranging from 0 to 1 (Fig. S2C, Supplemental Methods, Equation 3). High coherence implies tight phase locking and narrow phase-difference histograms, while low coherence indicates the opposite. Note when computing phase-difference histograms and coherence for two oscillators with integer frequency ratios (e.g., 1 or 2), we first multiply the phase of the slower oscillator by the frequency ratio, otherwise the phase locking is not reflected (Fig. S2D and E). Finally, a constant phase difference or phase-lag between two phases reflects an offset of the trajectory without affecting slope, and it is captured by the mean m of the histogram of joint phase-differences (Fig. S2F and G).

Seizure trajectories exhibited numerous band- and region-specific changes in phase-lag and coherence when transitioning from non-seizure to seizure states. For example, plotting five seizures on the unfolded MotL band 1–2 torus as in Fig. 2G, revealed a lack of association between phases outside of seizures (Fig. 2H, left) but confinement to a narrow region within seizures (Fig. 2H, right). Thus, plotting voltage as a function of band 1 and band 2 phases revealed stereotyped structure in seizure dynamics that is not apparent in the temporally diverging trajectories shown in Fig. 1D and E. In particular, during seizures, dynamics condensed along a narrow slope-2 path (gray dotted trajectory), with SWD-spikes (blue) clustered in a specific torus region (Fig 2H, right). A circular histogram of 2θ_1_ - θ_2_ from all mice (Fig. 2I) revealed increased coherence and a shifted phase-lag from the nonseizure to seizure state (Fig. 2I gray circle). The SomL band 1–2 torus also showed increased coherence and a shifted phase-lag (Fig. 2J gray circle) from the non-seizure to seizure state, with a SomL phase-lag that was distinct from the MotL phase-lag. These examples represent a small subset of within-brain-region but between-frequency-band phase relationships, showing that within-region phase-lags have coherences and mean phase-lags that vary substantially across Mot (L/R) and Som (L/R) region pairs (schematic in Fig. S2H, left; histograms for bands 1:2, 1:3, and 2:3 across regions in Fig. S2I).

Overall, these results show that while there is significant temporal drift of SWD-timing when comparing different seizures (Fig. 1D and E), the toroidal coordinate system captures strong regularities in SWD waveforms across many oscillation cycles (Fig. 2F–J).

### Projection onto a toroidal coordinate system describes between-region SWD time-lags

Fig. 1G and H showed robust correlations between-regions during seizures. To examine how well the toroidal coordinate system captures between-region oscillatory features, we compared phase-lag measures in the toroidal system to a specific between-region feature of SWDs: the time-lag of SWD-spikes between-regions. The tight within-region locking of the SWD-spike time with band 2 phase in Fig. 2D suggested that between-region band 2 phase-lags may distinctly inform the SWD-spike time-lags. Indeed, with our toroidal coordinate system we found a strong quantitative correspondence between SWD-spike time-lags and the corresponding band 2 phase-lags. Individual MotL-SomL SWD-spike lags were strongly correlated with the corresponding phase-lags (Corr=0.70) between MotL and SomL band 2 (MotLθ_2_-SomLθ_2_, schematic and example traces, Fig. 2L). Bands 1 and 3 were more-weakly correlated (Corr = 0.45 and 0.46 respectively) (Fig. S2J and K). Additionally, there was a tight correspondence between the mean and ranges of SWD-spike time-lags with the mean and ranges of band 2 phase-lags. Mean MotL-SomL SWD-spike time-lag was −8.2 ± 0.9 ms, indicating SomL spikes typically occur before MotL spikes (example traces Fig. 2K, left and mean time-lags Fig. 2K, right gray circles). Since band 2’s frequency is 15 Hz (a period of 67 ms), SWD-spike time-lags of −8.2 ms divided by the period of 67 ms times 2π, should correspond to a phase-lag of −0.77 ± 0.1 rad or approximately −π/4 rad. Similarly, the 95% range (~two standard deviations) of SWD-spike time-lags between −19.6 ms to +3.3 ms (purple and red circles respectively, Fig. 2K, right), corresponds to band 2 phase-lags ranging from −1.84 rad to + 0.31 rad, or equivalently from slightly less than −π/2 rad to slightly more than 0 rad. As predicted, the directly measured MotLθ_2_-SomLθ_2_ phase-lag was near −π/4 rad (−0.80 ± 0.10 rad) during seizures, with a 95% interval between −1.86 ± 0.29 rad and 0.27 ± 0.15 rad (gray, red, and purple trajectories and corresponding circles in Fig. 2M and N). We will see below that these values correspond to specific dynamical drives of absence seizures and region-specific burst firing at the single neuron level. As observed above for within-region band 1 and 2 tori (Fig 2F–I), the MotLθ_2_-SomLθ_2_ trajectories were confined to a specific torus region, here confined to the 95% phase-lag interval described above (Fig. 2M and N red and purple trajectories). Moreover, the MotLθ_2_-SomLθ_2_ phase histogram captured seizure vs. non-seizure features including increased coherence during seizure relative to nonseizure and a phase-lag shift to near −π/4 rad (−0.80 ± 0.10 rad) from the near 0 rad (0.09 ± 0.03 rad) phase-lag outside of seizure (Fig. 2N).

We observed a similarly tight correspondence between band 2 phase-lags and SWD-spike time-lags in SomL-SomR and MotL-MotR (Fig. 2O–R, Fig. S2K, see calculations in Supplemental Methods). In contrast to MotL-SomL, mean SWD-spike time-lags in MotL-MotR and SomL-SomR showed no timing delay between either transcallosal pair during seizures (MotL: −0.7 ± 0.4 ms and SomL: 0.42 ± 1.05 ms, Fig. 2O and 2Q Gray circles). This was correspondingly reflected by a ~ 0 rad mean phase-lag in band 2 for MotLθ_2_-MotRθ_2_ (−0.07 ± 0.05 rad, Fig. 2P schematic and histogram) and SomLθ_2_-SomRθ_2_ (0.07 ± 0.11 rad, Fig. 2R schematic and histogram). Coherence increased from the non-seizure to seizure state in both transcallosal region pairs with no change in mean phase-lag (Fig. 2P and 2R). In contrast to band 2, the SomLθ_1_-SomRθ_1_ band 1 phase difference showed no change in mean phase-lag or coherence from seizure to non-seizure (Fig. 2T), indicating a lack of substantial changes during the seizure state in band 1.

Overall, these are examples of one particular SWD feature (the SWD-spike time-lags) and the relation to a small subset of all possible phase-difference relationships which have numerous band- and region-specific changes (schematic in Fig. S2H; histograms Fig. S2I and L). The switch to a toroidal coordinate system provides a rich quantitative phenomenology of mean phase-lags and coherences between frequency bands and regions with accompanying amplitude variations that determine the relative timing and waveform of SWDs. The large number of possible phase-difference and amplitude relationships, make it extremely difficult in general to understand individual pairs of oscillatory relationships and their causes in isolation. However, when considered in aggregate, they form a powerful set of metric constraints for identifying dynamical models that evolve to a putative absence seizure attractor described by these relationships.

### Identifying a sparse dynamical system model that recapitulates seizure dynamics

To discover a dynamical mechanism capable of generating these numerous complex relationships, we applied a system identification approach: Sparse Identification of Nonlinear Dynamics (SINDy).^26^ SINDy expresses how the system evolves toward future states as a function of the current state (Supplemental Methods, equation 4), selecting model terms from a candidate function library (Supplemental Methods, equation 5). Model complexity is limited by using sparsity-promoting regularization (see Supplemental Methods). We focused on modeling the earliest SWD onset regions observed in Fig. 1, limiting the model to bilateral exemplars of the motor and somatosensory systems: MotL (PreMotL), MotR (PreMotR), SomL (FaceL), and SomR (FaceR). We learned a model that tracks the real (x) and imaginary (y) components of the Hilbert transform of the 3 frequency bands as in (Fig. 2B) across 4 regions, forming a 2*3*4=24 dimensional dynamical system.

We trained the model on a single-step prediction task using data from 400 ms before to 800 ms after seizure onset using 25 seizures from each of 5 mice. We then tested its ability to simulate 3 s of seizure activity from 25 different pre-seizure initial conditions (5 from each mouse) that were not in the training set. This held-out test performance was evaluated by errors in predicting multi-second amplitude dynamics across 3 bands × 4 regions (12 metrics, Supplemental Methods equation 6), phase-lag (Supplemental Methods equation 7) and coherence (Supplemental Methods equation 8) across bands-within regions (2* 4 regions * 3 frequency pairs = 24 metrics), and within-bands across 4 region pairs: 2 transcallosal (MotL–MotR, SomL–SomR) and 2 ipsilateral somatosensory motor (MotL–SomL, MotR–SomR), yielding another 24 metrics (Fig. S2H). Together, these 60 metrics provide a strong basis for assessing how well our model recapitulates the state space of held-out seizure dynamics over seconds long timescales of future state prediction. We define an overall error metric composed of the average of these 60 metrics to quantify model performance (Supplemental Methods equation 9).

### The identified system of dynamic equations defines a chaotic absence seizure attractor

**Figure 3.**
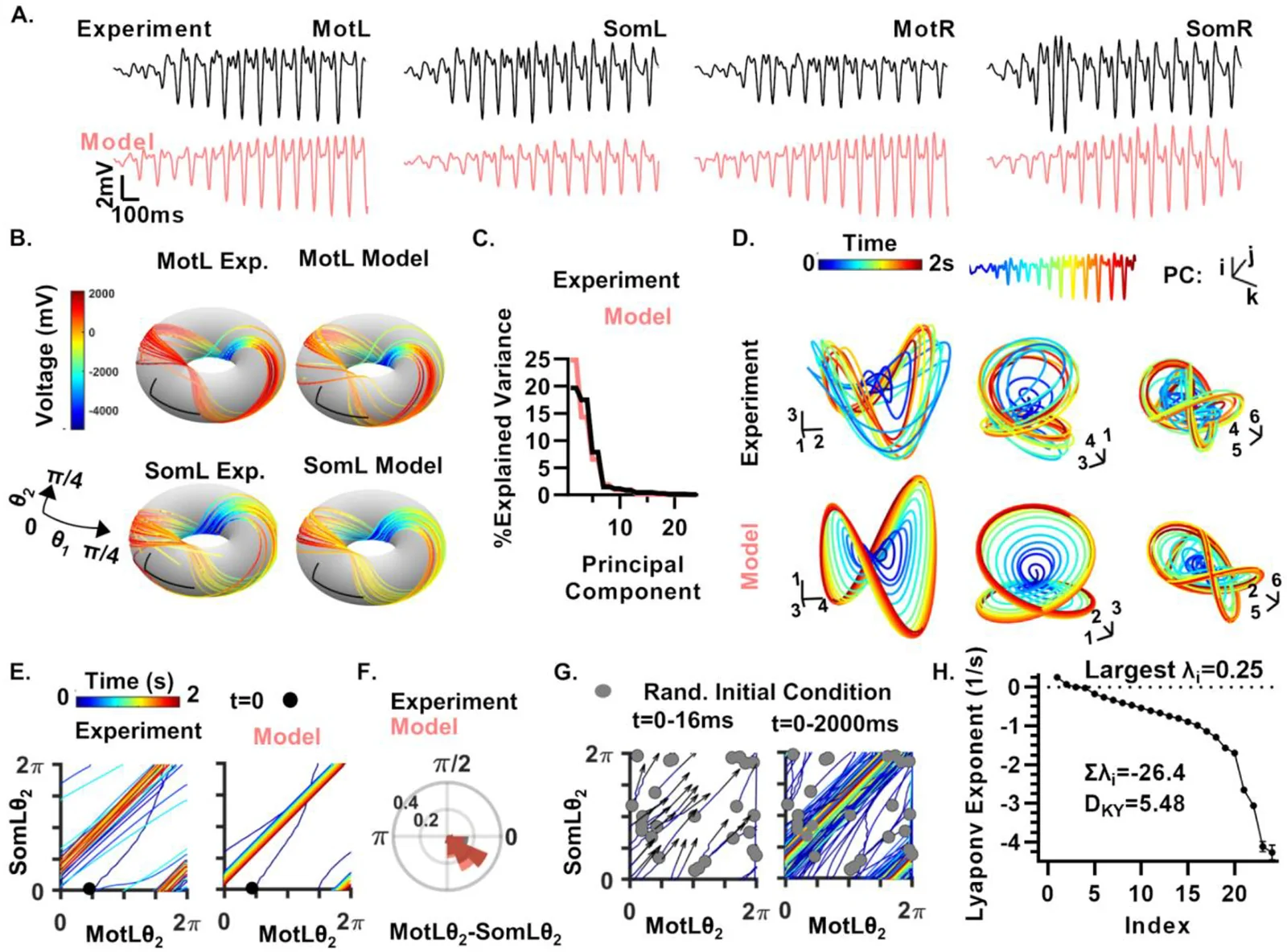
Sparse identification of nonlinear dynamics revealed governing equations for an absence seizure chaotic attractor (A) Experimental and simulated voltage traces during seizures. (B) Experimental and simulated dynamics on band 1:2 tori for MotL and SomL. Color indicates voltage as in Fig. 2G. (C) Percent variance explained by principal components (PCs) in experiment and model. (D) Example PC traces showing model simulation (bottom) and experimental (top) dynamics across the first six PC components; trajectories colored by time. Scalebars 1000 μV, numbered by PC number. (E) Time-colored trajectory on the band 2 Mot/Som torus from an initial condition outside the seizure state space. (F) Phase-difference histogram comparing model and experiment across 25 pre-seizure initial conditions as in E. See Fig. S3 I and L for all between-band and between-region comparisons. (G) Simulations from 25 random initial conditions, initially disorganized during the first 16 ms (left) converge to the seizure state space (right); trajectories colored by time as in E. (H) Lyapunov exponent spectrum from simulations as in Fig. S3F. Largest exponent λ>0 indicating a chaotic system. Sum of λs being negative indicative of an attractor. Kaplan–Yorke dimension (D_KY_) of 5.48 indicates the attractor is a fractal occupying a between 5 and 6 dimensional subspace.

Using these metrics to guide model selection (see Supplemental Methods), our model closely reproduced experimental data across regions, capturing both voltage waveforms (Fig. 3A) and toroidal dynamics (example band 1–2 torus dynamics shown in Fig. 3B). The model reproduced seizure features even outside the toroidal coordinate system, with principle component analysis (PCA) projections of experimental and simulated data showing strong similarity across the first six components, which explain 90.1% and 91.3% of total variance, in experiment and the model, respectively (Fig. 3C). These components contained oscillatory trajectories progressing from low-amplitude pre-seizure states (blue) toward a common region in PC space that is consistent between experimental data and the model (Fig. 3D).

We next examined trajectories toward the seizure state space from the pre-seizure initial conditions. An example torus of band 2 MotLθ_2_ and SomLθ_2_ phases is shown in Fig. 3E; both experimental and model trajectories followed a similar curved path towards the seizure state space, afterward following straight-line trajectories as described in Fig. 2M, right. The corresponding phase-difference histogram showed high agreement between model and experiment, both in mean phase-lag and coherence (Fig. 3F). Our model performed well across 60 such phase and amplitude metrics (Fig. S3A–D). We will see below that the interpretable nature of the model allowed us to uncover a mechanistic basis for this and other phase-difference relationships. Total model error did not increase in simulating data from within the training set (0.19) vs held out test data (0.19). Remarkably, the model aligned so closely with experiment that replacing the model predicted future dynamics of seizures from an individual mouse with seizures from another experimental mouse yields higher total error (0.24) than simulation from the specific pre-seizure state (0.19), an effect reflected across phase and amplitude metrics (Fig S3C and E). Thus, our model describes seizure properties with greater accuracy than the level of between-subject variability in these properties.

Moreover, late-time seizure dynamics indicated an attractor state. Random initial conditions (real and imaginary components sampled between ± 150 μV) all converged to a limited seizure state space. An example torus is shown in Fig. 3G during the first 16 ms (left) and after 2 s (right). Replacing seizure-specific initial conditions with random initial conditions increased prediction error only slightly from 0.19 to 0.23, indicating specific pre-seizure states, while tracked, were not necessary for reaching the attractor state.

To assess long-term stability, we analyzed 1200 s long model simulations which showed bounded oscillatory SWD dynamics (first 25 s in Fig. S3F). A recurrence plot of the final 120 s (Fig S3G) revealed strong aperiodicity across long timescales, indicating that the system does not settle into a simple limit cycle oscillation. However, on short time scales on the order of the SWD-spike interval of 133 ms, the model exhibits approximately periodic oscillations with cycle-to-cycle variations in amplitude and phase that differ across regions. This strong aperiodicity over long time scales suggests the learned dynamical system exhibits chaotic dynamics within an attractor region. We confirmed this by computing the Lyapunov exponent spectrum which indicated a 5.48 dimensional (Kaplan-Yorke dimension: 5.48), chaotic (largest exponent: 0.25/s), and dissipative system (sum of exponents= −26.43/s), indicating the presence of a chaotic attractor (Fig. 3H).

Overall, these findings show that our dynamical equations, learned directly from data both: (1) quantitatively account for 60 different metrics characterizing held-out seizure dynamics, not in the training data, at a high level of accuracy, comparable to that of inter-subject variability between individual mice, and (2) reveals that absence seizure dynamics are well modelled by a dynamical system containing a 5.48 dimensional chaotic attractor.

### Our dynamical systems model for absence seizures is highly sparse and interpretable

A priori, the final dynamical system could have been highly complex, with 1134 candidate functions across 24 variables (27,216 parameters). However, our iterative expansion and sparsification method of model selection yielded a much smaller model (see Supplemental Methods). The model was built by iteratively expanding simpler candidate-function libraries and incrementally testing terms for removal (Schematic: Fig. S4A). Several quantal drops in prediction error for our 60 seizure metrics occurred with the addition of specific libraries (Fig. S4B). In contrast, single-step cross-validated mean squared error poorly distinguished between libraries (Fig. S4C). This dichotomy reveals the fundamental discriminative power of our 60 seizure state-space metrics in performing model selection, rather than simply following the widely used method of single-step prediction performance. In particular, improvements in specific metrics appeared at different library stages - e.g., autonomous, between-band, or between-regional terms (Fig. S4D).

**Figure 4.**
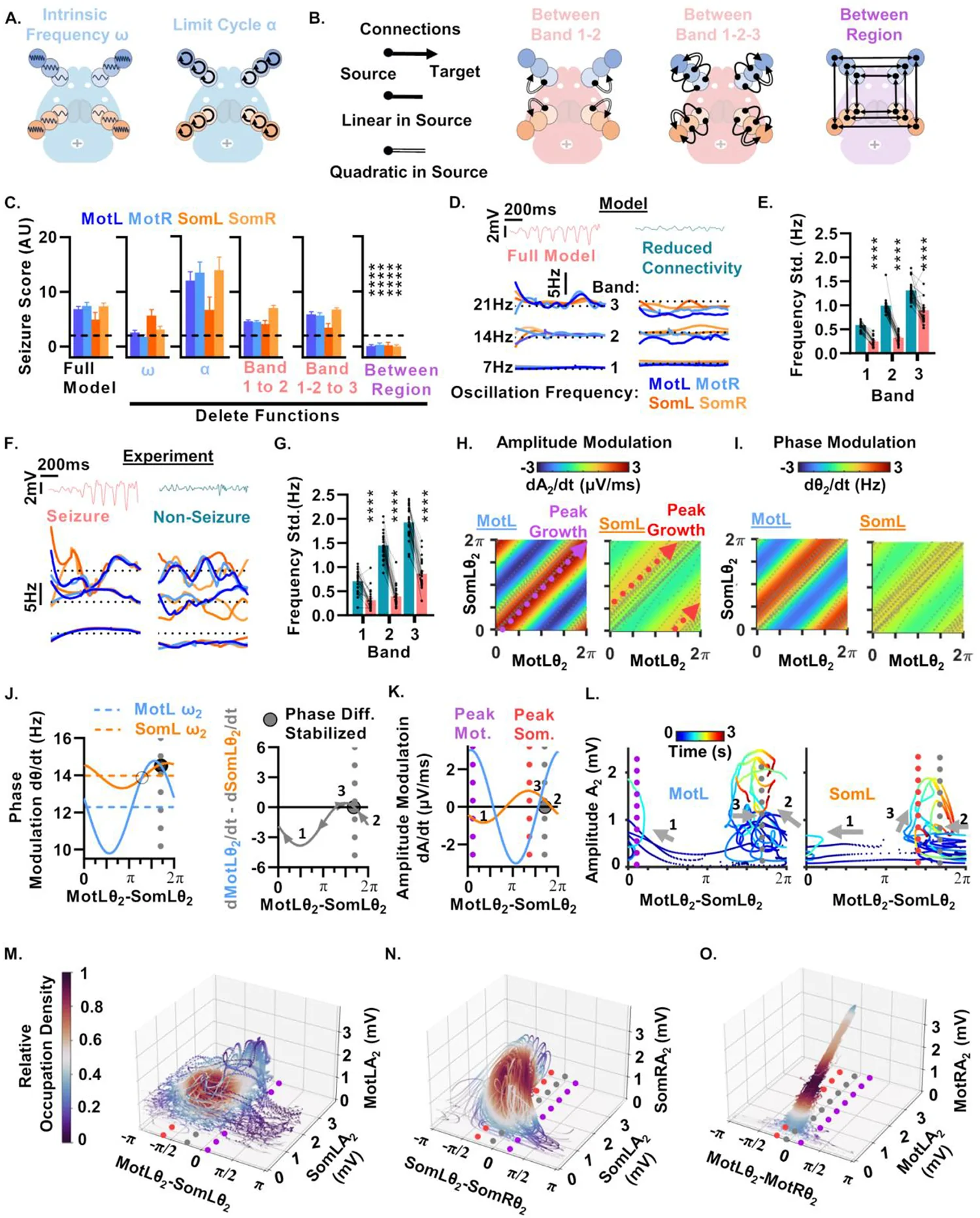
Between-regional coupling drives seizure generation through frequency locking driven amplitude growth (A and B) Schematics of dominant model terms. (A) Band- and region-specific autonomous terms combine analytically to form oscillators with intrinsic frequency ω and amplitude-damping limit cycle α. (B) Coupling terms include between-band-within-region band 1 to 2 coupling (left), band 1 and 2 to 3 coupling (middle), and linear between-regional-within-band coupling (right). See also Fig S4E for additional terms and Supplemental Methods for analytic simplification steps. (C) Effect on seizure score of removing term classes from A and B by setting learned coefficients to 0. Asterisks indicate significant reductions below seizure score = 2 (average amplitude growth < 2 z-score from baseline). (D and E) Representative voltage traces (D, top), oscillation frequency (D, bottom), and (E) between-region oscillation frequency standard deviation (SD) for the full model (left) versus 6× reduced connectivity strength (right); *n* = 25 simulations from 5 mice pre-seizure initial conditions. (F and G) Experimental data as in D and E during seizures versus non-seizures (*n* = 25 seizures, *N* = 5 mice). (H and I) Example between-regional amplitude (H) and phase (I) coupling functions plotted on the torus showing phase and amplitude modulation along an experimentally observed seizure trajectory (gray dashed line). See Fig. S5D–G for other coupling functions. Red and purple dotted lines mark peak amplitude growth trajectories for SomL and MotL respectively. Note correspondence to upper and lower observed phase-difference bounds in Fig. 2M. (J) Analytically simplified phase coupling function showing phase modulation of band 2 by phase difference frequency modulation around respective intrinsic frequencies (ω, dashed lines); stability points marked at intersections (circles). Phase-lag stabilization (gray dashed line, J, right) occurs where the difference between curves cross from positive to negative between regions marked 2 and 3. Region marked 1 causes strong decrease in phase-lag, leading to wraparound to high phase-lags at point 2. Note similarity of stability point to mean phase-lag in experiment (Fig. 2N) and model (Fig. 3F). (K) Amplitude modulation showing peak growth for Som (red dashed line) and Mot (purple dashed line) band 2 as in H. Note similarity to phase-lags of the 95% interval experimentally in Fig. 2M and N. (L) Five example trajectories of band 2 amplitude and phase-difference colored by time. Dynamics cycle around phase stability point marked by gray dashed line. (M-O) Examination of band 2 trajectories along the chaotic attractor. (M) Dynamics as in L for long simulations. (N) Dynamics of SomL-SomR. (O) Dynamics of MotL-MotR. See Fig. S5H for MotR-SomR. 95% ranges from Fig. 2NPR indicated by red and purple lines and mean phase-lag by gray. Statistical analyses were performed by one-sample (C) or paired Student’s *t* test (E and G); *N* = 5 mice, *n* = 25 simulations or seizures per condition. Data represent mean ± SEM.

Of 25 possible function libraries, only 11 contained selected (i.e. non-zero) terms. Each band/region included 43 terms on average. Many identified coefficients were near-identical multiples (see Supplemental Methods), enabling an analytic expression in the complex variable z=x+iy and conversion to polar coordinates z=Ae^iθ^. This left only 2 classes of autonomous terms (Fig. 4A), 2 classes of within-regional between-band interaction functions (Fig. 4B, left and middle), and 6 classes of between-regional interaction functions (one is shown in Fig. 4B, right and five are shown Fig S4E). The terms that most effectively improved model performance (see Fig. S4B) were in libraries 1, 2, 3, and 7, corresponding to the 5 functions shown in Fig.4A and B, while many between-region coupling terms shown in Fig. S4E (accounting for 46% of model terms) provided minor improvements individually, and removing them all increased the overall error metric slightly from 0.19 to 0.21 (Fig. S4F).

The sparse nature of our selected terms, obtained through model selection, yielded a mechanistically interpretable model. For each region and each band i=1,2,3, we denote the phase by θ_i_ and the amplitude by A_i_. Here autonomous dynamics (Fig. 4A) are defined via the dynamic equations dθ_i_/dt = ω_i_ and dA_i_/dt = α_i_A_i_^3^, with α_i_ <= 0. This describes an oscillator with intrinsic frequency ωᵢ (Library 1, Schematic: Fig. 4A, left), whose amplitude Aᵢ decays to zero via a cubic nonlinearity (Library 1, Schematic: Fig. 4A, right). Since in the data-driven model, each individual band in each region is stable in isolation, the generation of large amplitude epileptic oscillations solely relies on between-band or between-region interactions. Each such interaction term involved phases and amplitudes of oscillator pairs or triplets. Functions included quadratic band 1 to 2 between-band, within-region terms (Library 2, Fig. 4B, left); within-region cubic band 1 and 2 to 3 terms (Library 3, Fig. 4B, middle); linear between-region, within-band terms (Library 7, Fig. 4B, right); and several nonlinear between-region, between-band interactions (Libraries 15–21, Fig. S4E). These latter combined between-band, between-region functions contributed little to model accuracy (Fig. S4F). See Supplemental Methods for a mathematical description of all of these interaction terms.

In summary, from 27,216 potential parameters, our model selection identified an accurate and interpretable sparse dynamical model generating the dynamics of absence seizures, with only an average of 43 nonzero parameters per oscillator that are highly organized into interactions between bands and regions with different polynomial orders that can be combined analytically into a small number of interpretable functions.

### Strong between-region coupling and limited frequency dispersion are required for seizure generation.

To identify which classes of function drive seizures, we iteratively set each class’s terms to zero in the learned model. Seizures could only be abolished by removal of between-region within-band coupling (Fig. 4B, right, Fig. 4C, Fig. S4G). This suggests that while other terms contribute to model accuracy, the *large-amplitude seizure oscillations are driven exclusively by between-region coupling*. We then swept the weights of between-region within-band coupling between 0 and 1 and found that a six-fold reduction is required to abolish seizures (Fig. S4H). Doubling all terms within each individual function class did not suppress seizures for any function class (Fig. S4G), suggesting that no individual function class strongly promotes damping of seizures.

The central importance of between-regional coupling suggests that synchronization between regions plays a fundamental role in seizure generation. To test this hypothesis, we increased the spread of intrinsic oscillator frequencies in the model by shifting each one independently (see Supplemental Methods). Such dispersal of intrinsic oscillator frequencies is known theoretically to suppresses synchronization.^27^ See example shifts for band 1 in Fig. S4I. Indeed, seizure scores decreased across all regions with increased frequency spread between regions (Fig. S4J). Thus, as predicted, suppressing synchronization by dispersing intrinsic frequencies between regions prevented seizures.

Also consistent with the hypothesis that synchronization plays a key role in seizure generation, we found that each region and band converged to a common band-specific oscillation frequency (Fig. 4D). Furthermore, reducing between-region connectivity strength sixfold eliminated both seizures (Fig. 4D, top-right) and frequency convergence (Fig. 4D, bottom-right), as quantified by frequency standard deviation between regions (Fig. 4E). Similarly, increasing intrinsic frequency spread (Fig. S4I) disrupted frequency convergence (Fig. S4K, L).

Thus overall, strong between-regional coupling and limited intrinsic frequency spread are two key features leading to seizure generation in the model; if either frequencies are dispersed or coupling is reduced, both seizures and synchronization are abolished, as is expected in theories of synchronization phase transitions in coupled oscillators.^27^

### Frequency locking, as predicted by the model, is confirmed in experimental Ecog data

Our model predicted that between-region frequency spread drops to near zero at seizure onset. We confirmed this prediction in experimental data in which each band transitions from dispersed frequencies across regions to a single common frequency across regions (Fig. 4F, left). In contrast, outside of seizures (Fig. 4F, right), oscillator frequencies varied widely across regions (Fig. 4G). The non-seizure frequency spread (Fig. 4G) resembled that of the reduced-connectivity model (Fig. 4E). In contrast, the degree of frequency dispersion required to block seizures in the model (Fig. S4J) far exceeds the experimental observations in the non-seizure state (>4-fold, Fig. 4L). This suggests that the transition from non-seizure to seizure states in the brain originates from an increase in effective between-regional connectivity strength, rather than a decrease in intrinsic oscillator frequency dispersion.

### Synchronization and phase-lag dependent amplitude growth drive seizure generation

We next tested whether specific bands or couplings were required for seizures. Robust seizures persisted when the model was reduced to only the intrinsic oscillatory terms - intrinsic frequency *ω*, cubic damping *α*, and the seizure-driving between-regional, within-band coupling (Fig. S5A). This shows that between-region coupling is not only necessary but also sufficient to drive seizures in the model. Further reduction of the between-region coupling to only Mot–Som or transcallosal connections reduced but did not fully eliminate seizures (Fig. S5A). In the full model, seizures persisted when any single band or all Mot–Som or transcallosal couplings were removed in isolation (Fig. S5B and C). Thus, no specific band or regional coupling is essential. In essence all within-band, between-regional couplings collectively contribute to seizure generation (Fig. 4C).

To gain insight into the mechanisms of between-regional seizure generation, we next examined the form of these crucial between-region, within-band couplings (Library 7, Fig. 4B, right). Given the strong between-region frequency locking of band 2 in both the model (Fig. 4E) and the data (Fig. 4G), as well as the strong association of band 2 with the SWD-spike (Fig 2C–D) and between-region SWD time lags (Fig. 2K–S), we focus here on between-region couplings within band 2. However, we also show several other between-region, within-band couplings in Fig. S5D–G, as seizure generation does not depend exclusively on band 2 coupling (Fig. S5B). These coupling functions, as above, could be converted to analytic expressions in the complex variable z=x+iy and converted to polar coordinates z=Ae^iθ^ resulting in functions of the following form given a pair of regions and a single band:

Equation 1 $$\frac{dA_{j,B}}{dt}=C\;\text{cos}\left(\theta_{k,B}-\theta_{j,B}-\varphi_{1}\right)$$

Equation 2 $$\frac{d\theta_{j,B}}{dt}=C\frac{1}{A_{j,B}}\text{cos}\left(\theta_{k,B}-\theta_{j,B}-\varphi_{2}\right)$$

Here, A_j,B_ and θ_j,B_ denote the amplitude and phase respectively of target region j in band B and A_K,B_ and θ_k,B_ denote the amplitude and phase of a source region k (see Supplemental Methods). The constants φ_1_ and φ_2_ indicate preferred phase-lags in the specific source-to-target coupling. This form is followed by all between-region, within-band couplings, with transcallosal Mot couplings multiplied by an additional factor A_k,B_ in both equations. Remarkably, these couplings identified from only the real and imaginary components of experimental signals combined to resemble the paradigmatic Kuramoto model of synchronization of phase-coupled oscillators but with additional amplitude variation.^27^

Because seizures are defined by the growth of large amplitude oscillations from a low amplitude state (Fig. S1A–D), we first examined the form of the amplitude coupling functions which drive this amplitude growth. Equation 1 states that if the phase-lag *θ*_k,B_ – *θ*_j,B_ between the target region k and source region j is equal to the amplitude coupling’s preferred phase-lag φ_1_, then the target region amplitude A_j,B_ grows at a maximal rate. In contrast, if the phase-lag is the opposite at *φ*_1_ ± *π* then the amplitude decays at a maximal rate. Fig. 4H plots the band 2 amplitude coupling functions (Equation 1) from SomL to MotL (left) and MotL to SomL (right) as joint functions of the MotL-SomL phase-lag (i.e. on the phase torus). This revealed that the rate of amplitude growth occupies disjoint regions on the phase torus for MotL and SomL respectively. In particular, MotL amplitude growth occurs at a maximal rate when the MotL-SomL phase-lag is near zero (red region, purple trajectory in Fig. 4H, left). In contrast, in this same phase-lag region, SomL amplitude moderately decays (blue-green region in Fig. 4H, right). Conversely, when MotL-SomL phase-lag is slightly more negative than −π/2, SomL amplitude grows at a maximal rate (red region, red trajectory in Fig. 4H, right,), while MotL amplitude moderately decays (blue-green region of Fig. 4H, left). This result provides a hint that MotL and SomL amplitude growth will tend to be anticorrelated. We will see below that this anticorrelated growth corresponds to region specific bursting at a single neuron spiking level. Notably, these maximal growth trajectories correspond to the upper and lower bounds of the between region phase-lags (Fig. 2M and N), and thus between-region SWD-spike time-lags (Fig. 2K) observed experimentally.

Due to the dependence of amplitude growth on between-region phase-differences, we next examined the form of the between-region phase coupling functions. Fig. 4I shows band 2 phase coupling functions (Equation 2) from SomL to MotL (left) and MotL to SomL (right) as joint functions of the MotL-SomL phase lag. These coupling functions are shown at a fixed small amplitude of 200 μV for both regions, allowing us to understand phase modulation in the transition from the non-seizure to seizure state. In isolation, these functions do not explain how the MotL-SomL phase-lag stabilizes near a particular value in the seizure. To elucidate this, we plotted (Fig. 4J, left) the rate of phase advance (i.e. instantaneous oscillation frequency) arising from two sources: the region-specific autonomous intrinsic oscillation frequencies ω (dotted lines) and the sum of the intrinsic frequencies with the additional sinusoidal modulation arising from the between-region phase coupling (solid lines) between MotL and SomL band 2 (Equation 2). We examine the phase advance at fixed 200 μV amplitudes and discuss the amplitude variation effects below. The curves intersect (i.e. have equal oscillation frequency) at two points, with one notably near −π/4 rad (equivalently 3π/4, gray dotted line), corresponding to the mean phase-lag during seizures in experiment and model for MotL-SomL band 2 (Fig. 2M–N and Fig. 3F). The difference of the phase advance curves yields Fig. 4J, right, expressing the rate of change of the phase-lag between MotL and SomL as a function of the phase-lag itself. This plot reveals that this ~ −π/4 rad (= 3π/4) intersection is a stable fixed point at this fixed amplitude, with strongly decreasing phase-lag when at lower lags to the far left (arrow 1), leading to a wrap-around of phase-difference to higher lags to the right of the stable point (arrow 2), after which the phase-difference then continues to decrease towards the stable point. Just to the left of this stable point, the rate of phase-difference change switches directions, causing the phase-difference to now *increase* towards the stable point. Thus, the phase interactions between MotL and SomL (Fig. 4JK), at low fixed amplitudes, stabilizes their phase-lag at −π/4 rad, thereby explaining the mean phase lag observed in both model and experiment (Fig. 2N and Fig. 3F).

### Phase-amplitude coupling drives chaotic dynamics

The phase relationship at the above point would be a stable fixed point if amplitudes were clamped at 200 μV. However, the amplitude coupling functions show that at this phase-lag, there is a slight rate of amplitude growth in MotL *and* SomL band 2 (Fig. 4K, both orange and blue curves are positive at the fixed-point phase-lag –π/4 (= 3π/4) rad marked by the gray dotted line). Furthermore, due to the anti-correlated nature of MotL and SomL amplitude growth as a function of phase lag (Fig. 4H), we see in Fig. 4K that just to the right of the −π/4 rad phase lag (point 2), MotL amplitude grows faster and SomL amplitude moderately decays whereas to the left of this phase lag (point 3), MotL amplitude decays while SomL amplitude grows. Due to the 1/A_j_ scaling of the phase modulation curves, this causes the stable phase lag point to shift to the right towards 0 when SomL band 2 amplitude is larger and to shift to the left towards −π/2 rad when MotL amplitude is larger. Thus, in the combined MotL-SomL phase-amplitude feedback dynamics, the phase lag oscillates about −π/4 rad (gray dashed line) with the amplitudes growing and decaying (arrows 2 and 3) by oscillating between peak Mot (purple dashed line) and Som (red dashed line) growth at phase lags between 0 and - π/2 rad respectively while avoiding areas where both regions will decay (Fig. 4L). Overall, the reason the system grows to a net high amplitude (seizure) state is because in the narrow range over which the phase-lag oscillates (between −π/2 and 0 rad), the rate of change of amplitude is never strongly negative (Fig. 4K) and therefore cannot cause net decay to very low amplitudes. While SomL amplitude dynamics vary relatively smoothly as the phase lag oscillates about −π/4 rad indicated by arrows 2 and 3 (Fig. 4L, right), the MotL dynamics have additional variations in the high amplitude state (Fig. 4L, left), reflecting the additional transcallosal MotL-MotR coupling strength that grows multiplicatively with amplitude by A_k,B_.

In long simulations, the combined dynamics of phase-lag and SomL and MotL amplitude varied chaotically, with phase-lag oscillating, as expected, in the range of −π/2 and 0 rad (red and purple dashed lines respectively) about the mean overall phase-lag (~ −π/4 rad) (gray dashed line, Fig. 4M), as observed in *both* model *and* data (Fig. 2O, Fig. 3F). Brief destabilizations repeatedly returned to this basin. MotR–SomR showed similar behavior (Fig. S5H), while transcallosal Som coupling oscillated near 0 rad (gray dashed line) with repeated folding of the trajectory (Fig. 4N), reflecting the high coherence and 0 phase-lag observed in model and data (Fig. 2R, Fig. S3B). This is consistent with the structure of SomL-SomR couplings (Fig. S5F). Transcallosal Mot coupling varied in amplitude but was tightly locked near 0 rad (gray dashed line, Fig. 4O), again consistent with model and experiment (Fig. 4P, Fig. S3B) and the MotL-MotR coupling functions (Fig. S5G). Notably, the MotL-MotR couplings have phase and amplitude modulation growing with an additional factor of A_k_, explaining the tighter locking observed in model (Fig. S3B) and experiment (Fig. 2P). Altogether these results provide a comprehensive mechanistic understanding of the phase and amplitude relationships described in Fig. 2 and Fig. S2, which together define absence seizures as a chaotic dynamical attractor (Fig. 3).

Finally, while we have focused here on within-band between-region couplings, we analyze the complementary between-band, within-region couplings (see Supplemental Methods for these coupling functions). We show that these couplings only become strong at large amplitudes, thereby causing band 1–2 phase synchronization and SWD waveforms to appear in high amplitude seizure states (Fig. S5I and J), but not outside seizures as was observed in experiment (Fig. 2G–H. Fig. S2I). *Thus, SWD waveforms result from rather than cause the oscillation amplitude growth during seizures*.

Overall, these within-band, between-region coupling functions reveal a mechanism for seizure generation through synchronization-induced amplitude growth. Basically, each coupling function prefers a certain low-amplitude stable fixed point phase-lag between source and target oscillators. Arrival at this preferred phase-lag leads to amplitude growth. Then feedback coupling between amplitude dynamics and phase-lag dynamics induces a slow variation about the low amplitude stable fixed point phase-lag in the high amplitude state. Moreover, this variation appears to be chaotic with continued variations in amplitude and phase on the time scale of seconds, thereby suggesting a mechanistic origin for the overall chaos quantified in Fig. 3H. In summary, synchronization near the preferred phase-lag of pairs of oscillators induces amplitude growth in both oscillators. Below we will employ these model-derived explicit dynamical mechanisms for amplitude growth to uncover corresponding neuronal circuits and single neuron spiking patterns.

### Single neuron spiking in a Som-Mot-PO subnetwork frequency locks to the fundamental seizure frequency at seizure onset

**Figure 5:**
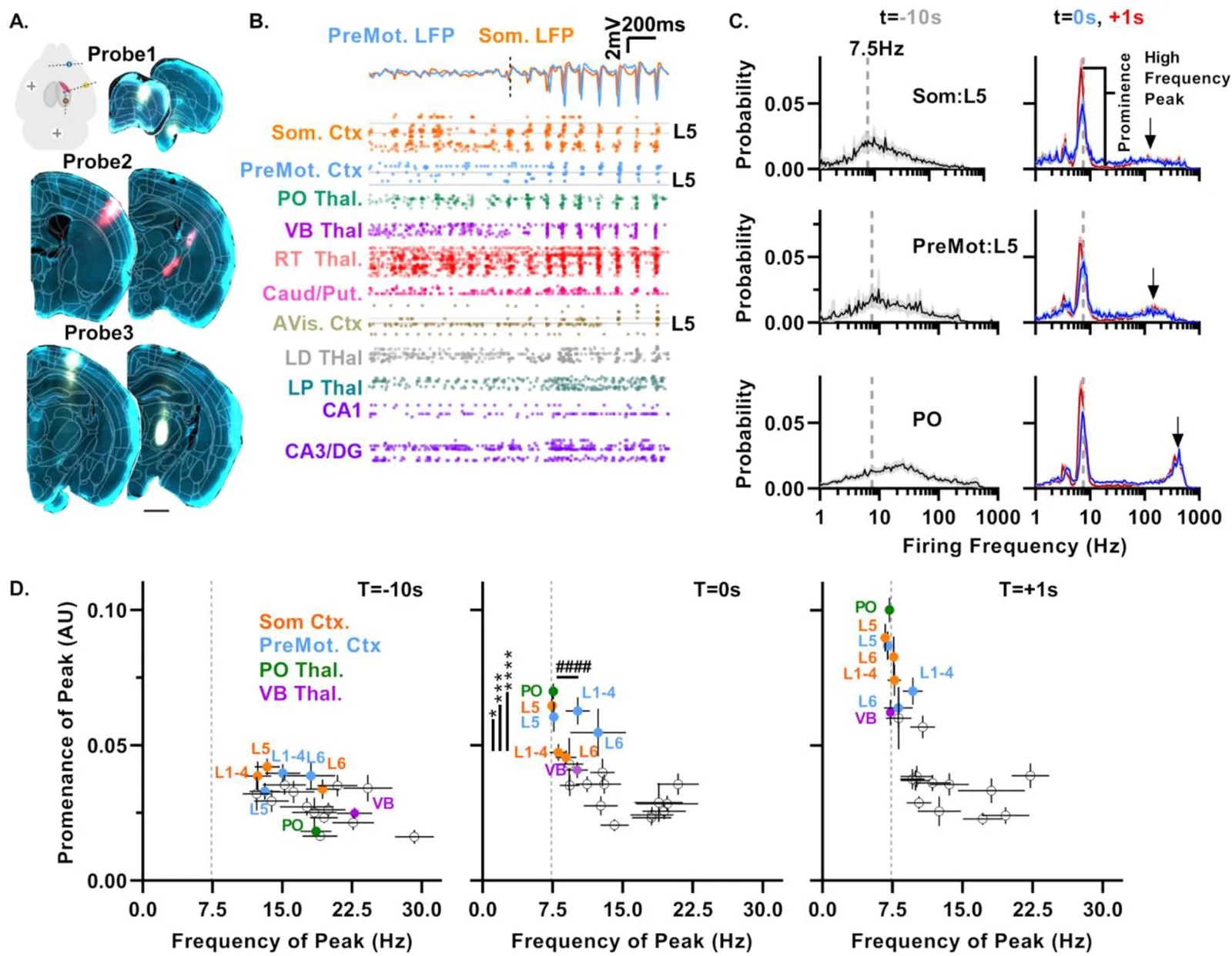
Neuronal population frequency locking identified Som:L5, PreMot:L5, and PO as an early seizure network. (A) Experimental design showing Neuropixels probe and SomL screw electrode placements for simultaneous Ecog and neuronal spiking recording. DiI/DiD-labeled probe tracts mark insertion points and maximum depths; white outlines denote region boundaries from SharpTrack alignment. Scale bar = 1 mm. (B) Simultaneously recorded spike rasters from single units across regions and Ecog in PreMot and Som Face regions during a single seizure. Black dashed line indicates seizure onset. Gray lines indicate L5 boundaries. Units marked by depth within region. (C) Instantaneous firing-rate histograms for Som:L5, PreMot:L5, and PO averaged across seizures (5 per recording, 6–15 recordings, from 4–8 mice), error bars indicate SEM. Time relative to seizure onset: −10, 0, +1 s. The window size of 2 s around these times. Prominence calculated as difference between peak and average left/right of peak 7 frequency bins away. (D) Scatterplots of frequency of peak vs. prominence. Dashed line marks the 7.5 Hz seizure fundamental frequency. At seizure onset (T=0), PO, Som:L5, and PreMot:L5 cluster at this frequency. Asterisks indicate significance for prominence for PO, Som:L5, PreMot:L5 vs Som:L1–4, the next closest to frequency locked population. Welch’s t-test. Hashtags indicate significance for frequency variance vs Som:L1–4 and PreMot:L1–4, both p<0.0001. At T=+1 s, PO, Som:L5, PreMot:L5 show highest peak prominence, with other regions converging to the fundamental.

We next examined whether single neuron spiking reflected Ecog features and model predictions. Using three simultaneous Neuropixels probes in awake mice, along with simultaneously recorded Ecog from surface and contralateral somatosensory electrodes (Fig. 5A), we mapped activity across brain regions during absence seizures. The Neuropixels probes were labelled with dyes DiI/DiD/DiO marking their position in the brain (Fig. 5A pink and yellow regions), and we identified single units through spike sorting (Fig. S6A–B). Together, this allowed alignment of spike rasters to Ecog SWDs in PreMotor and bilateral Som Face/Barrel cortex (Fig. 5B). Cortical units were classified as excitatory or inhibitory by waveform risetime (Fig. S6C). Mean firing rates showed only moderate region-specific changes (Fig. S6D) from non-seizure to seizure states. Notably, Som deep layers showed *decreased* overall firing (Fig. S6D, top-left), consistent with recent findings of no hyperexcitability in Som during or preceding absence seizures.^3–6^ These results suggest seizure organization depends more on spike timing and firing mode (burst vs. non-burst) than on overall firing rate.

Since our model predicted frequency locking, at the level of LFP across regions (Fig. 4DE) and we confirmed this prediction in Ecog data (Fig. 4EG), we next examined if frequency locking at the level of individual neurons would inform which networks drive this activity. We measured region-specific histograms of the reciprocal of interspike intervals (i.e. instantaneous spike frequencies) for all neurons in the region, obtained from 2 second time windows centered at different time points relative to seizure onset (nonseizure at −10s, onset at 0s, and +1s after onset corresponding to gray, blue, red histograms, respectively in Fig. 5C). We used peaks in the population’s spike interval distribution as indicators of frequency clustering, where concentrated spiking at a specific frequency produces an elevated count at that frequency and reduction at neighboring frequencies, quantified by peak prominence (Fig. 5C). We found no frequency locking in the nonseizure state as indicated by low prominence at the peak frequency (gray histograms in Fig. 5C, left, Fig. 5D left), but observed strong locking of firing frequencies to the fundamental seizure frequency near 7.5Hz at onset, which was sustained through the seizure in specific populations (blue and red histograms in Fig. 5C, right, Fig. 5D, middle, right). We also observed smaller isolated peaks at high frequencies > 40Hz (arrows Fig. 5C, right), indicative of bursting, which increased in both thalamus and cortex (Fig. S6F,G). The strongest spike frequency locking at seizure onset, (blue, Fig. 5C, right), occurred in somatosensory cortex layer 5 (Som:L5), premotor cortex L5 (PreMot:L5), and posterior thalamic nucleus (PO) (blue Fig. 5C, right, and Fig. 5D, middle). These regions all exhibited higher peak prominence than the next nearest population to the seizure frequency (Som:L1–4) (Fig. 5D). These populations also exhibited very low variance in the frequency of the peak at onset across seizures 0.34 (PO), 5.1 (SomL:5), 8.3 Hz (PreMot:L5) compared to next highest prominence region: (PreMotL:2–4, 77.2 Hz) and next population closest to the fundamental seizure frequency (SomL:L1–4, 32.9 Hz). This highly consistent frequency locking of PO, Som:L5, and PreMot:L5 indicates these regions are more reliably recruited to a common seizure frequency at onset than other regions (Fig. 5D). These regions also had the highest peak prominence after seizure onset (Fig. 5C, right, red, and Fig. 5D, right). Notably, other regions such as ventrobasal thalamus and the other Som and Mot cortical layers are strongly recruited to the locked state after onset (Fig. 5D, right).

These results support an *early seizure network linking Som:L5 and PreMot:L5 with PO*. Importantly, these three populations are highly interconnected and reflect the Mot and Som early seizure network indicated in Fig. 1C. *PO projects to the apical dendrites of layer 5 pyramidal neurons* in both of these regions and both L5 pyramidal populations are reciprocally connected.^28–30^ The recipient zone for connections from PO to these apical dendrites are located both in layer 1 and layer 5. Intriguingly, layer 1 apical dendrites are also the primary recipient of cortico-cortical feedback inputs from other cortical regions and prior work has found that PO thalamus boosts the effectiveness of input at these distal sites.^31, 32^ This anatomy then suggests that PO inputs could broadly modulate the strength of feedback coupling from Som:L5 to Mot layer 1 and Mot:L5 to Som layer 1 along with transcallosal connections in both regions during seizures.^33^ We next examine the relationship between LFP and single neuron spiking dynamics focusing on these three tightly locked interconnected regions.

### LFP amplitude growth predicted by model coupling functions is associated with single neuron bursting in the target region

We have seen above that LFP in band 2 strongly reflects between-region SWD-spike timing lags (Fig. 2K–R) and shows a strong increase in coherence from non-seizure to seizure states (Fig. 2NPR). Furthermore, we found that phase-lags between pairs of regions determine whether respective LFP amplitudes in each region grow or decay in the model (e.g. Fig. 4H for SomL and MotL, and S5D-G for other pairs of regions). However, single neuron spiking frequency locks near the fundamental band 1 frequency (7.5 Hz) (Fig. 5C), not the higher band 2 frequency (15 Hz). This raises a fundamental question of how band 2 phase-lags and thus ultimately the spike of the SWD relates to individual neuronal spike times underlying the seizure.

**Figure 6:**
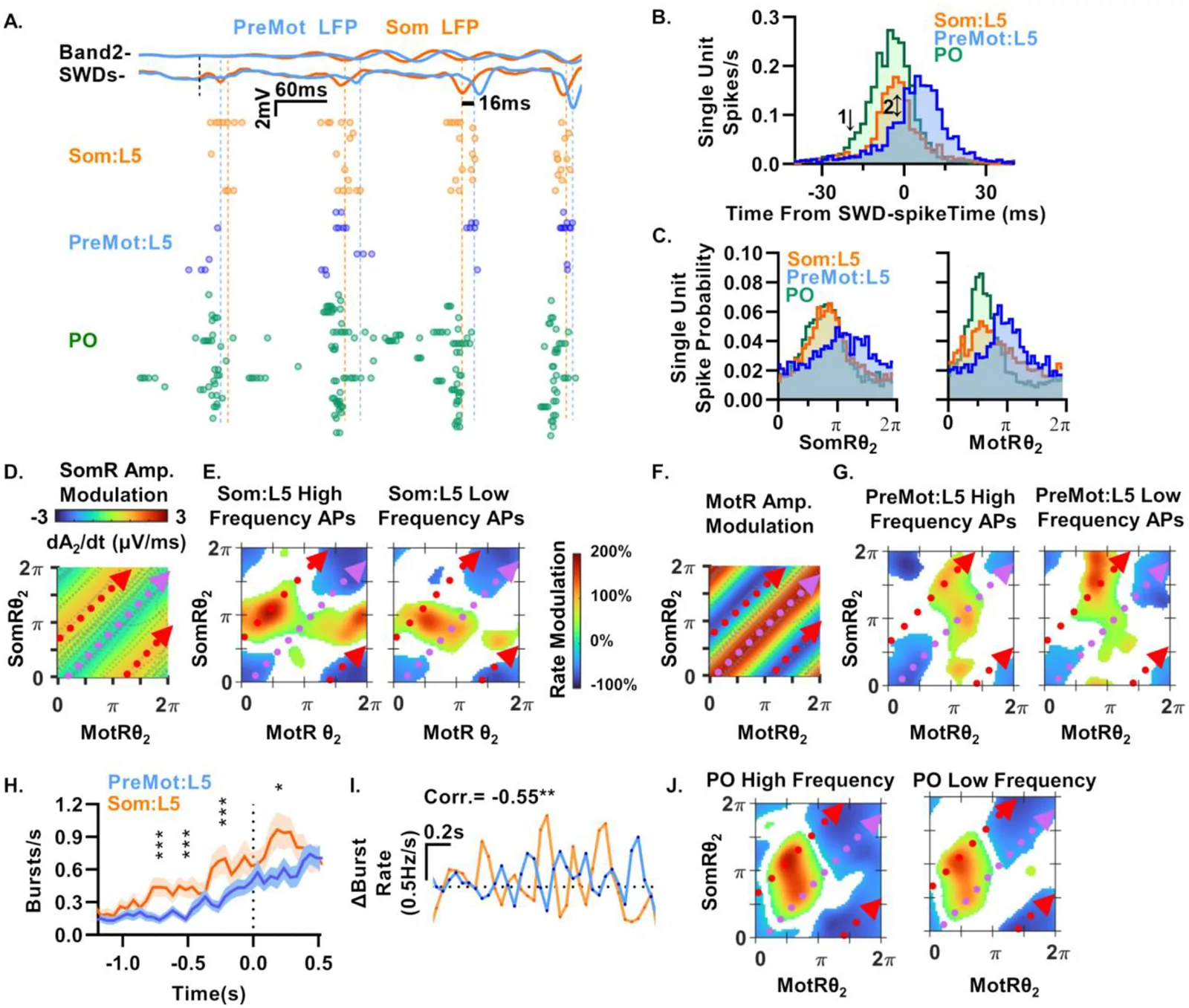
Amplitude coupling functions corresponded to region specific burst and tonic firing mode. (A) Expanded view of raster shown in Fig. 5B showing band 2 alignment (top) with full spectrum Ecog (middle) and unit firing in Som:L5, PreMot:L5, and PO. Orange and blue dashed lines mark trough of band 2, roughly aligning to the peak of the SWD-spike. Units sorted by unit ID. Note the variable separation of spiking clusters across regions on a cycle-by-cycle basis. (B and C) (B) Som SWD-spike triggered peristimulus time histogram. Arrow 1 indicates early elevation of PO firing above Som:L5 or PreMot:L5 (both p<0.0001, Mann-Whitney U test). Arrow 2 indicates PreMot:L5 shoulder overlap with Som:L5 firing peak. (C) Neuron-spike triggered phase histograms for band 2. (D) Amplitude coupling function of SomR, red dashed line marks peak SomR amplitude growth, purple dashed line marks peak MotL Amplitude growth, corresponding to SomR amplitude decay as Fig. 4H. (E) Som:L5 high-frequency firing modulation (left) and low-frequency firing (right) on the phase torus. Note peak amplitude growth trajectory (red line) traverses peak high-frequency firing (red region) followed by silencing (blue region) while amplitude suppressing trajectory (purple) traverses modest high-frequency firing (green region) (E, left) while low-frequency firing is elevated along both trajectories. (F) As in D for MotR modulation. Note opposing area of amplitude growth and decay from D as in Fig. 4H. (G) As in E for MotR high-frequency and low-frequency firing. Note MotR amplitude growth marked by purple trajectory traverses increase and decrease high-frequncy firing (left) while the amplitude suppression trajectory (red) traverses increased high-frequency and increased low-frequency firing without negative modulation. (H) Average number of detected bursts (a >40 Hz interval preceded by a <10Hz interval). Asterisks indicate significant differences between regions. (I) Correlation of change in burst rate from H showing changes in Som:L5 and PreMot:L5 burst rates are anticorrelated. (J) As in E and G for PO high-frequency (left) and low-frequency (right) modulation. Note the increase and decrease along both the Mot and Som amplitude growth trajectories.

To address this, we examined the relationship between band 2 oscillations (Fig. 6A, top) in Som and Mot and the full spectrum unfiltered LFP containing the SWDs (Fig. 6A, middle) relative to single neuron spiking (Fig. 6A bottom). Recall that band 2 phase-lags between Mot and Som oscillate between 0 to −π/2 rad (Fig. 2N), which corresponds to a range of SWD-spike time-lags of ~ 0 to −15 ms (Fig. 2K). Moreover, note that a phase-lag of ~0 corresponds to peak amplitude growth in MotL (Fig. 4H, left) while a phase-lag of ~−π/2 rad corresponds to peak amplitude growth in SomL (Fig. 4H, right). We focused on Som:L5, Mot:L5, and PO neurons because these were the first to show strong single neuron frequency locking at seizure onset (Fig. 5D, middle). The temporal relationship of neuronal spikes to the SWD-spikes varied on a cycle-by-cycle basis (Fig.6A). The peristimulus time histogram (PSTH) of single-neuron spikes in Som:L5, PreMot:L5, and PO relative to the Som SWD-spike, revealed that the Som:L5 single unit spikes occurred preferentially −4 ms before the Som SWD-spike (Fig. 6B, blue) while PreMot:L5 single neurons preferentially fired +4 ms after (Fig. 6B, blue). Notably the time-lag between single neuron spikes closely resembled the time-lag of −8.2 ms between the SWD-spikes in these regions (Fig. 2K), reflecting time-lags found previously.^34^ PO single neuron spikes exhibited the highest peak, occurring at −6 ms before the Som SWD-spike (Fig. 6B, green). Notably, there was considerable overlap between these populations over a range of −15 to +15 ms, with PO exhibiting an earlier increase in spiking around −20 ms before the Som SWD-spike (Fig. 6B arrow 1) compared to Som:L5 or PreMot:L5 (both p<0.0001, Mann-Whitney U test), while Som:L5 and PreMot:L5 did not differ from each other at this timepoint. While the PreMot:L5 peak trailed the Som:L5 peak by 8 ms, there was a substantial shoulder (47% of the peak firing) of the PreMot:L5 distribution aligned with the Som:L5 peak (Fig. 6B arrow 2).

The parallel between PreMot:L5-Som:L5 single neuron spike time lags (Fig. 6B) and Mot-Som SWD-spike time lags (Fig. 2K), together with the correspondence between SWD-spike time and band 2 troughs (Fig. 2N), suggest that the variable timing of single neuron spikes in these regions could be informed by band 2 phases. Single spikes in these regions were indeed modulated by Som:L5 and PreMot:L5 band 2 phases (Fig. 6C), but these phases in isolation did not clarify spiking relationships between regions. However, we unearthed a strong association between single neuronal spikes and LFP phases by examining when spiking occurs as a *joint* function of *both* Mot and Som band 2 phases, i.e. on the band 2 phase torus. This analysis is motivated by our model, since the joint phases of Mot and Som determine seizure amplitude growth in a manner not computable by knowing Mot or Som phases alone (Fig. 4H).

Fig. 6D shows the model SomR amplitude growth rate as a joint function of SomR and MotR phases. For reference, a hypothetical red constant phase-lag trajectory is shown to mark the region of maximal amplitude growth rate of Som as in Fig. 4H, indicated by the red regions at a MotRθ_2_-SomRθ_2_ phase lag of approximately −π/2 rad. Similarly, a hypothetical purple constant phase-lag trajectory is shown to mark the region of maximal Som band 2 amplitude decay rate, indicated by the light blue region at a phase-lag near 0 rad. As explained above (Fig. 4H–M) seizure trajectories (shown as dotted gray lines in Fig. 6D) wander between the hypothetical purple and red trajectories, leading to temporally varying Som and Mot amplitude growth and decay which leads to net amplitude growth in both regions.

We next plotted a 2-D histogram of spiking timing changes in SomR:L5 as a joint function of SomR and MotR phases. In particular for each successive interspike interval (ISI) we classified the ISI as either high-frequency (greater than 40Hz or ISI < 25 ms, Fig. 6E, left) or low-frequency isolated spikes (less than 40Hz or ISI > 25 ms, with neighboring spike frequency <= 7.5 Hz, Fig. 6E, right). We assigned each ISI a joint MotR-SomR phase of the recorded LFP at the time of the second spike of the ISI. We then constructed a normalized rate modulation index (see Supplemental Methods) of where in MotR-SomR phase plane high-frequency ISI’s occurred (Fig. 6E, left) and separately where low-frequency ISI’s occurred (Fig. 6E, right). Green to red regions indicate where the respective ISI’s occurred at a higher rate than chance, for example a +100% modulation being a 2-fold increase in spikes relative to expected. Blue regions indicate where the ISI’s occurred at a lower rate than chance, for example a −50% modulation being a 2-fold decrease relative to expected. White regions indicate no statistically significant elevated or reduced ISI rates relative to chance. Chance levels were determined by computing ISI distributions in shuffled data (see Supplemental Methods).

Fig. 6E, left (Som:L5 high-frequency firing) and Fig. 6E, right (Som:L5 low-frequency firing) thus separately correlate high- and low-frequency single neuron spike pairs in SomR:L5 with joint LFP phases in SomR and MotR. It revealed several regularities. First, the red phase-trajectory line which produces the peak amplitude growth in SomR (Fig. 6D), passes through a substantial red region indicating an excess of high-frequency ISIs (maximum 187% above expected, a 2.87-fold increase), followed by a blue region indicating a significant deficit of high-frequency ISIs (minimum: −66% below expected, a 2.94-fold reduction), relative to the expected values (Fig. 6E, left). This pattern of successive ISI’s is characteristic of a burst. Thus, bursting in SomR:L5 neurons is strongly associated with MotRθ_2_-SomRθ_2_ phase-lags that lead to seizure amplitude growth in SomR. In contrast, the purple trajectory travels through regions with silencing of high-frequency firing (blue regions, minimum 68% below expected, a 3.13-fold reduction) and no substantial increase (small green region, maximum 41% above expected, a 1.41-fold increase Fig. 6E, left). Instead, the purple trajectory experiences a transient increase (111% above expected, a 2.11-fold increase) in low-frequency ISI’s followed by a decrease (63% below expected, a 2.7-fold reduction) (Fig. 6E, right). The combined silencing of high-frequency ISIs but increase of low-frequency ISI’s along the purple trajectory indicates an overall increase then decrease in isolated spikes, and absence of bursts. Thus MotRθ_2_-SomRθ_2_ phase-lags that lead to seizure amplitude decay in SomR are primarily associated with single spikes, not bursts. In summary, SomR:L5 bursts preferentially occur in the MotR-SomR LFP phase plane region that leads to SomR amplitude growth (red trajectory), while isolated single spikes preferentially occur in disjoint regions of the MotR-SomR phase plane that lead to SomR amplitude decay (purple trajectory).

Fig. 6F–G analyzes the relationship between MotR LFP amplitude growth or decay and MotR:L5 isolated spikes or bursts in a manner parallel to Fig. 6D–E. Indeed, we find a completely parallel result: MotR:L5 bursts preferentially occur in the MotR-SomR LFP phase plane region that leads to MotR amplitude growth, while isolated single spikes preferentially occur in disjoint regions of the MotR-SomR phase plane that lead to MotR amplitude decay. We can see this as follows. The purple reference trajectory which leads to MotR amplitude growth near 0 rad phase-lag (red region in Fig. 6F) traverses significantly increased (111% above expected, a 2.11-fold increase) then decreased (68% below expected, a 3.13-fold reduction) high-frequency ISI rates (purple trajectory in Fig. 6G, left) with a modest increase (57% above expected, a 1.57-fold increase) in low-frequency ISIs along this trajectory (green region and purple trajectory, Fig. 6G, right). This pattern is again indicative of bursting. In contrast, the red reference trajectory which leads to MotR amplitude decay near −π/2 rad phase-lag (red trajectory, blue region in Fig. 6F) experiences increased rate of high-frequency ISIs (107% above expected, a 2.07-fold increase) that is *not* followed by a significant decrease in the rate of high-frequency ISIs (red trajectory in Fig. 6G), but is rather accompanied by significantly increased rate (144% above expected, a 2.44-fold increase) of low-frequency ISIs (red trajectory Fig. 6G, right). This pattern is indicative of an overall increase in the rate of isolated spikes without an increase in bursts.

Thus, in both SomR and MotR we have found a strong correlation between bursts, MotR-SomR phase-lags, and SomR/MotR LFP amplitude growth. In particular, in both brain regions, bursts primarily occur at LFP phase-lags that lead to LFP amplitude growth for that region, while isolated spikes primarily occur at LFP phase-lags that lead to LFP amplitude decay. This tight correspondence leads to a striking prediction regarding bursts as follows. First, comparing Fig. 6D and Fig. 6F, we note that MotR amplitude growth and decay regions are disjoint from SomR amplitude growth and decay regions in the MotR-SomR phase plane. Thus, as discussed for amplitude growth in the model (Fig. 4K–M), either SomR amplitude grows and MotR amplitude moderately decays (red trajectory in Fig. 6DF) or MotR grows and SomR moderately decays (purple trajectory in Fig. 6DF). Now given the strong association between bursts and amplitude growth in both regions, our analysis predicts that bursts in the two regions should be anticorrelated in time; in essence when MotR bursts more, SomR should burst less and vice-versa.

We tested this prediction directly at level of bursts without reference to LFP. We examined cortical bursting in SomR:L5 and PreMotR:L5 at seizure onset. We found that SomR:L5 bursting increased 4.4-fold relative to the non-seizure state (0.22 ± 0.07 vs 0.97 ± 0.16 bursts/s p<0.0001, Welch’s t-test) and PreMotR:L5 increased 6.2-fold (0.12 ± 0.04 vs 0.74 ± 0.11 bursts/s, p<0.0001, Welch’s t-test). This increased bursting preceded seizure onset (Fig. 6H) and, as predicted by our model, burst rates in SomR:L5 and MotR:L5 were indeed anti-correlated in time across these two regions (Fig. 6I).

Finally, since PO thalamus is the most strongly frequency locked region at the level of single neuron spikes (Fig. 5D, middle and right), and since PO projects to both Mot:L5 and Som:L5, we further examined the relationship between spikes in PO and the MotR-SomR LFP. In Fig. 6J we plot the rate of PO ISIs relative to chance, as a joint function of SomR and MotR phase, in direct analogy to Fig. 6E for SomR:L5 spikes and Fig. 6G for PreMotR:L5 spikes. Intriguingly, we find that the rate of high-frequency ISIs undergoes a significant increase then decrease (bursting) along both the red and purple reference trajectories (Fig. 6J, left) as well as a similar increase then decrease in low-frequency ISIs (Fig. 6J, right). This indicates that no matter whether SomR amplitude is growing and SomR:L5 is bursting (red trajectory), or MotR amplitude is growing and PreMotR:L5 is bursting (purple trajectory), PO is bursting in both cases.

The strong connection between bursting in a brain region, and LFP amplitude growth, also held in transcallosal coupling from SomL to SomR. For example, the band 2 coupling function from SomL to SomR indicated SomR amplitude growth at 0 rad phase-lag and SomR amplitude decay at π rad phase-lag (Fig. S7B). Correspondingly, the phase trajectories that were associated with SomR LFP amplitude growth from SomL drive, were also associated with increased bursting in both SomR:L5 neurons and PO neurons (Fig. S7C) and phase trajectories associated with SomR LFP amplitude decay, were associated with increased spikes and not bursts in SomR:L5 (Fig. S7C, left and middle). We also examined several other brain areas, cell type, and firing modes associated with the phase tori (Fig. S7D–M). Notably, cortical inhibition followed anticorrelated patterns between Som/Mot regions and opposed to those observed for bursting above. In the thalamic reticular nucleus (TRN, the major inhibitory thalamic nucleus) high-frequency firing exhibited a strong association with the Som amplitude growth trajectory (Fig. S7I). These results suggest the bursting above is further modulated by or dependent on coordination of inhibition in the cortex and thalamus. Non-seizure related regions lateral posterior thalamic nucleus (a higher-order visual system analogue of PO) and hippocampal CA3/DG neurons showed no association with the phase torus (Fig. S7L).

Overall, in pairs of source and target regions (e.g. Som-Mot, Mot-Som, or SomL-SomR) where our model predicts target region LFP amplitude growth as a joint function of source and target band 2 phase-lags, we find the target region exhibits more bursting. Conversely, when our model predicts LFP amplitude decay, the target region exhibits more isolated spikes and less bursts. Finally, when any of these target regions exhibit amplitude growth and bursting, PO also bursts.

### Posterior thalamic nucleus activation enhances premotor to somatosensory functional connectivity

**Figure 7:**
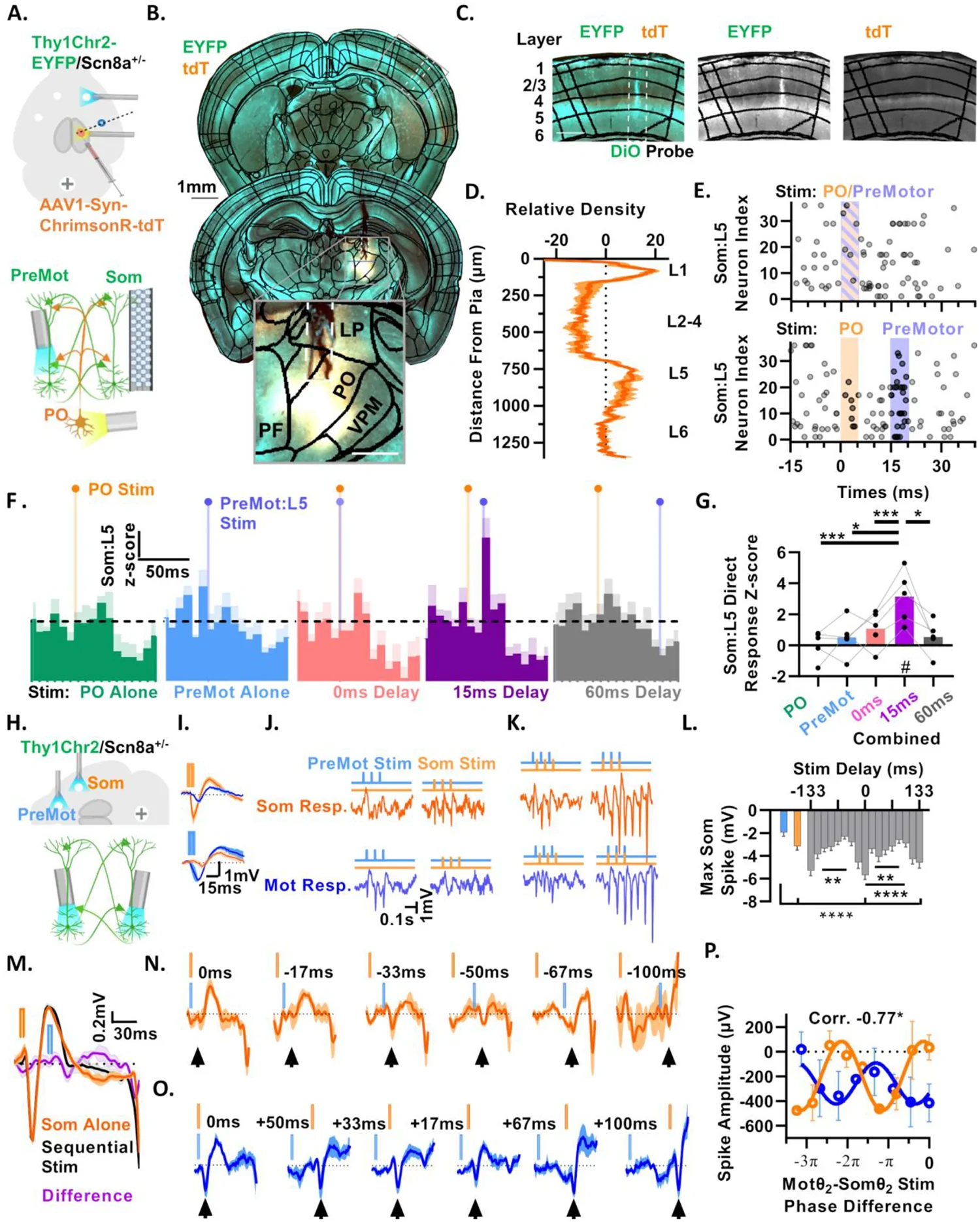
PO thalamus gates the PreMot:L5–Som:L5 pathway to promote absence seizures. (A) Experimental schematic: dual-fiber implants in *Scn8a+/−/Thy1-ChR2* mice for optogenetic stimulation of PreMot:L5 and PO while recording single-unit firing in Som:L5 Face/Barrel cortex. (B) Example histology of chrimsonR-tdT injection in PO and labeled fibers in L1 and L5 of Som cortex. Boundaries defined by SharpTrack. (C) (Left) Expanded view of recording site from (B) with DiO labeled probe (green vertical tract in white box), Thy1-GFP (green) expression in L1/L5, and tdT+ PO fibers (orange). (Middle) Green channel; (right) red channel. (D) Quantification of PO fiber density relative to mean along cortex depth at recording sites. (E) Som:L5 neuron spike raster during combined PO–PreMot stimulation at 0ms (top) or 15 ms (bottom) delay. Raster overlay from 10 trials. (F) Average of z-scored peristimulus time histograms (PSTHs) for PO, PreMot, or combined stimulation at 0, 15, or 60 ms delays for 5 recordings from 4 mice, error bars indicated SEM. Orange (PO) and Blue (PreMot) circles indicate time of stimulation. (G) Quantification of direct stimulus responses at 15–20 ms bin following initial stimulation (# indicate one-way t-test vs 0, asterisk indicate between group comparisons via paired t-test). (H) Experimental schematic: dual fiber implants in *Scn8a*^*+/−*^*/Thy1-ChR2* mice for optogenetic stimulation of PreMot:L5 and Som:L5 cortex. (I) Average with SEM effect of Som:L5 (top) and Mot:L5 (bottom) stimulation in Mot (blue) and Som (Orange) Ecog from 4 mice. (J an K) Stimulation patterns and resulting Ecog LFPs for PreMot and Som stimulation: single site (J) and combined antiphase (K left) or aligned (K right) stimulation. (L) Average minimum spike amplitude across 10 trials under each time-lag condition. Lines show pairwise t-tests. (M) Effect of Som:L5 stimulation alone (orange trace), the sequential stimulation of Som:L5 and then Mot:L5 33 ms later (black trace), and the difference between the two above traces to isolate the effect of Mot:L5 stimulation at a particular time-lag after Som:L5 stim (purple). (N) Average traces of the effect of Mot:L5 stimulation in Som Ecog at different time delays after Som:L5 stimulation after subtraction of Som:L5 stimulation alone as in M. Times indicate Mot-Som stimulation time-lag. Arrows indicate timing of spike. (O) As in N for the effect of Som:L5 stimulation in Mot Ecog. Times indicated Mot-Som stimulation time-lag. Order aligned to equivalent Mot-Som band 2 phase-lag of Som in O. (P) Circles indicate average spike amplitudes in N and O. Solid lines indicate sinewave fit. Asterisk indicates significant anticorrelated spike amplitudes at the given band 2 phase-lags.

Our model suggested that high between-region connectivity drives seizures (Fig. 4CD). Previous work has shown that PO activity can increase the effectiveness of inputs to Som:L5 and PreMot:L5 pyramidal neuron distal apical dendrites.^31^ These dendrites are precisely the site of PreMot:L5 to Som:L5 and Som:L5 to PreMot:L5 and transcallosal Som:L5 to Som:L5 and Mot:L5 to Mot:L5 feedback connectivity.^29, 31^ Moreover, Som:L5 and PreMot:L5 bursting followed their respective connection specific amplitude growth trajectories (Fig.6D–G), while PO bursting increased along both growth trajectories (Fig. 6J) as well as the SomL-SomR growth trajectory (Fig. S7B–C). This suggests PO’s role may be to increase overall cortico-cortical functional connectivity. We thus decided to directly test whether PO activity enhances functional connectivity between these areas in the context of absence seizures. In *Scn8a*^+/−^*/Thy1-ChR2* mice, we implanted an optical fiber in PreMot:L5, injected AAV–ChrimsonR in PO, and placed a second optical fiber above PO (Schematic in Fig. 7A). This setup allowed us to optogenetically stimulate PreMot:L5 pyramidal cells and PO thalamocortical cells independently with two different light wavelengths. Injections were largely confined to PO (Fig. 7B) with tdT labeled projections limited to Som:L1 and 5 (Fig. 7C and D). Som:L5 firing was recorded with Neuropixels during optogenetic stimulation of either PreMot:L5, PO, or both. When stimulating both, we stimulated PO and then PreMot:L5 with a delay of either 0 ms, 15 ms, or 60 ms corresponding to band 2 phase-lags of 0, −π/2, and 2π rad respectively. The choice of delay of 15 ms was motivated by the early increase in PO firing (Fig. 6B arrow 1) which occurred −14 ms before the peak in Som:L5 firing and was simultaneous with the PreMot:L5 shoulder (Fig. 6B arrow 2). These variable delays allowed us to determine how the temporal lag between PO and PreMot input to Som:L5 determines the effectiveness of PreMot input to drive Som:L5 spiking.

We found that stimulating PO 15 ms (but not 0 ms or 60 ms) before stimulating PreMot:L5 induced robust Som:L5 firing during the PreMot:L5 stimulation window (Fig. 7EF). Notably, stimulating PO alone or PreMot:L5 alone induced no subsequent strong direct spiking increase in Som:L5 response (Fig. 7F, left 2 plots). Over longer time-scales after stimulation, a subset of stimulation cases exhibited Som:L5 reduced firing followed by a subsequent increased rebound, triggering an oscillation (Fig. S8B–D). Notably, stimulating PO then PreMot:L5 at 0 ms or 15 ms delay triggered a transient seizure like oscillation at the fundamental frequency of endogenous seizures (Fig. S8E). In contrast, stimulating PO alone or PreMot:L5 alone did not, whereas stimulating PO then PreMot:L5 at 60 ms delay triggered a transient oscillation at a much lower frequency than that of endogenous seizures (Fig. S8E).

In summary, only stimulation of PO then PreMot:L5 at a 15 ms time-lag induced *both* a direct response in Som:L5 *and* a transient seizure like oscillation. This causal effect is consistent with endogenous seizure dynamics wherein PO firing becomes substantial −15 ms before (Fig. 6B arrow 1) a shoulder of significant PreMot:L5 firing (Fig. 6B arrow 2), which is coincident with peak Som:L5 firing.

### Multisite optogenetic stimulation of Som:L5 and PreMotor:L5 cortex cooperated in phase lag-dependent seizure generation

The model predicted LFP seizure amplitudes grow due to phase-lag dependent coupling between regions in an anticorrelated manner. For example, at particular values of the band 2 Motθ_2_-Somθ_2_ phase lag, Som amplitude grows due to Mot input (red region and red trajectory, Fig.4H, right) but Mot amplitude does not grow due to Som input (blue region Fig. 4H, left). However, at opposite values of the band 2 Motθ_2_-Somθ_2_ phase-lag Mot amplitude grows due to Som input (red region and purple trajectory, Fig. 4H, left) but Som amplitude does not grow due to Mot input (light blue region, Fig. 4H, right). Moreover, the timing of the band 2 trough is strongly correlated with that of the SWD-spike (Fig. 2CD), and between-region time-lags of the band 2 trough reflect those of the SWD-spike (Fig. 2K, right and Fig. 2N). Therefore, we decided to test whether SWD-spike amplitude responses in Mot and Som were sensitive to, and indeed anticorrelated with respect to, temporal lags in the stimulation of PreMot:L5 and Som:L5, as suggested by our model. To do so, we implanted *Scn8a*^+/−^*/Thy1-ChR2* mice with optical fibers in PreMot:L5 and Som:L5, allowing us to independently stimulate PreMot:L5 and Som:L5 pyramidal cells while recording Ecog signals in both regions in layer 1 (Fig. 7H). We delivered 3 pulses to each region at the fundamental seizure frequency of 7.5 Hz, at a low enough intensity that a single pulse would not strongly propagate to the other regions. Pulses were delivered to PreMot alone, Som alone, or to both with delays between the first pulses spanning −133 ms to +133 ms.

On average, PreMot:L5 stimulation in isolation produced small local Mot SWD like Ecog responses (−1.1 ± 0.03 mV) peaking at 9.5 ms post stimulation onset, with a much smaller (−0.23 ± 0.16 mV) nonlocal Som Ecog response that peaked at 15.9 ms post stimulation onset (Fig. 7I, top). Som:L5 stimulation in isolation showed the reverse response pattern (Som peak = −1.5 ± 0.33 mV at 5.7 ms, Mot peak = −0.42 ± 0.13 mV at 14.3 ms, Fig. 7I, bottom). Fig. 7J shows single trial examples of the trial average in Fig. 7I, again showing that stimulation of one region alone at low intensity does not induce seizure like oscillations. However, large SWDs were only obtained when stimulating both PreMot:L5 and Som:L5, which depended strongly on the relative timing of stimulations. Recall the fundamental 7.5 Hz seizure period is 133 ms. Stimulating using half this relevant time-lag (−67 or +67 ms did not yield large SWD-spikes (Fig. 7K, left), but stimulating at 0 time-lag did (Fig. 7K, right). Indeed, the response to stimulation depends strongly on the stimulation time-lag from −133 ms to +133 ms as measured by the largest SWD-spike amplitude during stimulation (Fig. 7L and Fig. S8F) and by the seizure score in the post stimulation period (Fig. S8G). Remarkably, stimulation near −17 to 0 ms time-lags (the time-lags corresponding to phase-lags predicted to lead to amplitude growth for Som and Mot band 2 respectively) induced self-sustaining seizures, but stimulation near ± 67 ms did not (Fig. 7K and Fig. S8G).

To test our prediction of anticorrelated regional responses as a function of the time-lag in stimulation, we determined the specific contribution to the response in one region due to input from the other region at different time-lags relative to the first stimulation. To do this, we subtracted the response to stimulation in one region from the response to stimulation of both regions (e.g. response in Som due to both Som:L5 and PreMot:L5 stimulation minus response in Som due to Som:L5 stimulation alone (Fig. 7M), and similarly for responses in Mot). This differential response reflected specific contributions to the response in one region from the other region at defined time-lags and is plotted for several different stimulation time-lags (PreMot:L5 stimulation to Som contribution, Fig. 7N, and Som:L5 stimulation to Mot contribution, Fig. 7O). In both cases the between-region contribution, as measured by the SWD response peak after stimulation from the opposite region (Fig. 7NO, black arrows) displayed a strong dependence on stimulation time-lag. The variation in spike amplitude varied with a timing dependence resembling the frequency of band 2. Fitting a sinewave to these curves revealed a periodic effect with a frequency near band 2 (Mot: 13.4 ± 1.3 Hz, R^2^=0.74 and Som: 12.2 ± 0.6 Hz, R^2^=0.92) (Fig. 7P). The phase offset was õpposite between regions (Mot phase=−0.50 ± 0.52 rad vs 2.04 ± 0.29 rad p=0.0078).

We thus plotted the evoked SWD-spike like amplitude response against the stimulation time-lag in units of the band 2 Mot-Som phase difference (Fig. 7P). This showed the maximal response in Som and Mot due to specific contributions from the other region were indeed anticorrelated: at band 2 phase-lags where Mot responds strongly due to Som input, Som responds weakly due to Mot input, and vice versa (Corr. = −0.77). This is reminiscent of the disjoint regions of amplitude growth in Mot and Som as a function of band 2 phase-lag between Mot and Som (Fig. 4HK).

Overall, these results directly demonstrate a multi-area seizure generation mechanism, in this case emerging from interactions between PreMot:L5 and Som:L5. Disjoint between-region phase-lags lead to either Mot SWD-spike growth or Som SWD-spike growth, specifically due to input from the other region at specific time-lags (Fig 7P), in direct analogy to how our model predicts that disjoint phase-lags lead to band 2 amplitude growth in Mot or Som (Fig. 4HK).

## Discussion

### Interpretable machine learning reveals an emergent mechanism for a low dimensional chaotic attractor state underlying LFP dynamics in absence seizures

Our brain-wide Ecog recordings (Fig. 1A) revealed a multiregional somatosensory and motor seizure network whose LFP amplitude rapidly and simultaneously elevates at seizure onset (Fig. 1C). While individual seizure trajectories rapidly and chaotically diverged in time from each other (Fig. 1DE), robust correlations between regions within a seizure were maintained (Fig. 1GH). To describe the underlying oscillatory seizure dynamics, we examined mean phase-lags, coherences, and amplitude trajectories between three harmonic bands across 4 representative regions (Fig. 2, Fig. S2HIL), resulting in 60 metric properties of the seizure that were impossible to understand in isolation. However, we used interpretable machine-learning to identify a dynamical model that simultaneously reproduced all 60 metric properties to within a precision matching inter-subject variability (Fig. 3, Fig. S3A–E, Fig. S4B). Moreover, our model revealed that the seizure state corresponds to a low 5.48-dimensional (Fig. 3CD) chaotic (Fig. 3H, Fig. S3FG) attractor state (Fig. 3EGH).

The model also revealed a simple between-regional dynamical mechanism for the origins of LFP synchronization, amplitude growth, and chaos. Our data-driven learned model remarkably converged to a coupled Kuramoto oscillator type interaction^27 35–37^ between brain regions with additional amplitude dynamics (Equation 1 and 2, Fig. 4BHIJK, Fig. S5D–J), that can lead to more complex phenomena.^38^ Both in the Kuramoto model and in our amplitude enhanced model, synchronization between regions occurs when between-regional coupling strengths overwhelm between-region heterogeneity in intrinsic oscillation frequencies, leading to multiple regions oscillating at a common locked frequency near seizure onset (Fig. 4DE), a model prediction which we successfully confirmed in the experimental data (Fig. 4FG). We previously showed reduced heterogeneity in voltage-gated sodium channels promotes synchronization and epilepsy by reducing intrinsic firing frequency heterogeneity, consistent with decreased biophysical heterogeneity in human epilepsy.^39–41^ However, here increased coupling strength better explained frequency locking in experimental seizures than did reduced intrinsic frequency spread (Fig. 4D–F, Fig. S4KL).

Near the onset of synchrony, regions start to oscillate at various relative phase-lags. Analysis of the between-region couplings of the model show that these couplings drive LFP amplitude growth in a phase-lag dependent manner (Equation 1). For example, if a pair of regions 1 and 2 oscillate at the coupling from region 2 to region 1’s preferred phase-lag, then the coupling will cause rapid amplitude growth in region 1. However, if they oscillate far from the preferred phase-lag, there will be reduced or no amplitude growth in region 1. Often times, the reverse coupling from region 1 to region 2 has a different preferred phase-lag. The combined amplitude and phase dynamics of the system would then cause the phase-lag between region 1 and region 2 to vary over time between these two phase-lags, leading to alternating periods of amplitude growth or no growth (or even moderate decay) in region 1 and region 2. This type of bidirectional coupling between-region pairs features prominently, for example in MotL-SomL (Fig. 4HK), and MotR-SomR (Fig. 6DF). This leads to a temporally alternating scenario where Mot amplitude grows due to Som input, but Som amplitude does not grow in response to Mot input, and then the opposite where Som amplitude grows due to Mot input, but Mot amplitude does not grow in response to Som input. Moreover, the alternation between these two scenarios is chaotic, because the combined feedback dynamics of amplitudes and phases amongst multiple regions itself is chaotic (Fig. 4M–O, S5H), as quantified by positive Lyapunov exponents (Fig. 3H). Importantly, the interpretable nature of our model allowed us to identify the cell types and firing properties involved: PO potentiation of between-region L5 cortical connectivity that drives phase-lag dependent burst firing.

Our model reveals an emergent mechanism for seizure genesis that arises from phase-lag dependent amplitude growth through many between-regional couplings resulting in a complex chaotic attractor state, rather than seizures originating in and then spreading from a single brain region. Discovering and understanding these between-region dynamic drives for seizure generation would not be feasible without the use of data-driven interpretable machine learning. The chaotic dynamics and non-reducibility of seizure drive to a single region, cell type, or constant firing pattern indicates fundamental constraints on how we can understand, predict, or control seizures. For example, because an emergent mechanism arises from many interacting processes, none of which causes the seizure on its own, reduction of seizure causes to single brain regions, cell types, or proteins in isolation will inevitably produce confounding results. Importantly, our results are at odds with a focal mechanism, cortical or otherwise, and provides an explicit mechanism for an emergent origin of absence seizures as indicated previously.^9, 42^

### The dynamic LFP model predicts the spatiotemporal organization of single neuron spiking

We next moved beyond LFP to a finer scale of analysis at the level of single neuron spiking across cortex, basal ganglia, hippocampus, and thalamus (Fig. 5AB). Remarkably, the dynamic behavior of our LFP model allowed us to reveal striking regularities in the spatiotemporal organization of individual spikes in a manner not possible without the model. First, we found individual neuron spiking moved from a heterogeneous distribution of frequencies in the non-seizure state to a locked common frequency in the seizure state (Fig. 5CD). This analysis was directly motivated by the analogous behavior of LFP, first predicted in the model (Fig. 4DE) and then confirmed in experiment (Fig. 4FG). Recent studies have reported heterogenous changes in the mean single neuron firing rates at absence seizure onset.^3, 4^ Our work explains such heterogenous changes as they are exactly what is required to achieve a common locked spike frequency across neurons in the seizure state: neurons that fire at higher (or lower) spike rates in the non-seizure state must lower (or raise) their firing rates to fire at a common rate in the seizure state. Importantly, analysis of the time dependence of this frequency locking (Fig. 5D) revealed an early seizure network consisting of single neurons in PO, Som:L5, and PreMot:L5, that were the earliest and most strongly frequency locked regions (Fig. 5C and Fig. 5D, middle, right). Consistent with this observation, these three populations have strong anatomical interconnectivity.^28–30^ We therefore focused our subsequent spiking analyses on these three areas, first focusing on the relationship between spiking and LFP.

Single neuron spikes occurred at a wide range of temporal lags with respect to LFP variables like the SWD-spike (Fig. 6AB) and SomR or MotR band 2 phases (Fig. 6AC). On the surface, these observations in isolation did not clarify a correspondence between spike patterning and LFP. However, guided by our model, we found a tight correspondence between band 2 between-region phase-lags, alternating amplitude growth in Som and Mot, and alternating tonic versus burst firing in single neuron spiking. Indeed, a prominent feature of spiking in the seizure state is the prevalence of bursts in addition to tonic single spike firing (Fig. 5C and Fig. S6FG). We analyzed when tonic spiking versus bursting occurred in both Som:L5 and PreMot:L5 as a function of the band 2 phases Somθ_2_ and Motθ_2_ and we found a striking correspondence: when the model predicts LFP amplitude growth in a given region (Fig. 6DF, Fig. S7A), that region exhibits more bursting (Fig. 6EG, left, Fig. S7C left); conversely when the model predicts amplitude decay (Fig. 6DF, Fig. S7B), that region exhibits more tonic firing (Fig. 6EG, right, Fig. S7C, middle). Given that amplitude growth in Mot and Som is anticorrelated over time due to chaotic variation in the Motθ_2_-Somθ_2_ phase-lag (either Mot amplitude grows and Som does not, or vice versa), our analysis then predicts that bursting in Som:L5 and PreMot:L5 should be anticorrelated in time (either Som:L5 bursts and PreMot:L5 does not, or vice versa). We directly confirmed this striking prediction in the data (Fig. 6HI).

This analysis reveals a privileged role for band 2 in relating LFP to SWD-spikes. Indeed, at the level of LFP alone, the trough of band 2 is strongly correlated with the timing of the SWD-spike (Fig. 2CD, Fig. S2A) and between-region band 2 phase-lags captured the corresponding between-region SWD-spike time-lags (Fig. 2K–R, Fig. S2JK). Now connecting LFP to single neurons, the SWD-spike occurred with an approximately 4 ms delay relative to peak single neuron spiking (Fig. 6B). Also given that band 2 phase-lags determine both amplitude growth (Fig. 6DF) and burst versus tonic firing (Fig. 6EG), a natural hypothesis is that band 2 LFP at 15 Hz predominantly reflects the time course of the calcium plateau and repolarization associated with cortical burst firing, while the slower 7.5 Hz interval of single neuron spiking (Fig. 5CD) reflects an inability of neurons to fire again until the second cycle of band 2, mediated for example by inactivation of sodium and calcium channels.^32^ These results are consistent with previous observations that absence seizures appear to arise from the progressive amplification of every second trough of a spindle-like oscillation with a frequency of twice the SWD-spike interval frequency.^12^ Fittingly, the first line anti-absence seizure medication ethosuximide blocks T-type calcium channels which promote cortical and thalamic burst firing, which by our analysis above is associated with LFP amplitude growth.^4, 43^ This suggests disruption of the underlying drive towards the absence seizure attractor is the therapeutic target of the leading anti-absence treatment.

### Higher order thalamus amplifies cortico-cortical functional connection strength during absence seizures

In our LFP model, increased coupling strength between-regions drives seizures (Fig. 4C, Fig. S5G). Also, our analysis of the higher-order thalamic nucleus PO, part of the early seizure network with Som:L5 and PreMot:L5 (Fig. 5D) revealed that on each seizure cycle, PO spiking increases before Som:L5 and PreMot:L5 (Fig. 6B) and PO bursts with both of them as a function of the band 2 Motθ_2_-Somθ_2_ phase-lag (Fig. 6EGJ). PO has been implicated in absence epilepsy previously with some of the earliest pre-seizure activity changes.^44^ Moreover, previous work has found that PO projects to both cortical layers 1 and 5 of Som and PreMot, and potentiates feedback coupling between layer 5 pyramidal neurons in these two regions by depolarizing their apical dendrites, lowering the threshold for layer 1 inputs to trigger somatic spikes.^31^ Somatic inputs, when paired with a strong coincident or slightly prior distal input, elicit amplified dendritic depolarization and voltage gated sodium and calcium channel dependent burst firing.^45–47^ This distal input could in principle be provided by PO inputs early in the SWD-spike oscillation cycle (Fig. 6B) which would then prime PreMot:L5 and Som:L5 apical dendrites to promote burst firing when long range input from other regions arrive, either from the PreMot:L5/Som:L5 connections or from transcallosal inputs to these regions.

We tested this hypothesis experimentally with multisite optogenetics, observing that stimulation of PO 15 ms before stimulating PreMot:L5 causes a large increase in Som:L5 firing when PreMot:L5 is stimulated (Fig. 7E–G) and triggers oscillations at the fundamental seizure frequency (Fig. S8E). In contrast, stimulation of PreMot:L5 or PO in isolation does not. Thus, we conclude the strong coupling between regions in the model that drives seizures is mediated by PO-induced amplification of cortico-cortical L5 functional connectivity across the motor and somatosensory systems. Additionally, these same mice show post-seizure onset myelination increases resulting from myelin plasticity, suggesting a contribution of a structural connectivity increase.^48^

Notably, neurons in PreMot and Som not only receive projections from PO but also send projections back to PO. This suggests future investigation of the effect of top-down cortical control of PO could further refine this mechanism. This top-down control could be mediated through direct cortico-PO projections, or indirectly via projections to the basal ganglia or thalamic inhibitory nucleus RT.^49^ Correspondingly, burst frequency corticothalamic fiber stimulation drives RT dependent post-inhibitory rebound bursting and oscillation frequency modulation of thalamocortical neurons.^7^ Our prior work showed *Scn8a* knockdown in RT can cause absence seizures and disrupts RT-RT inhibition.^24^ Furthermore, RT-RT inhibition powerfully suppresses thalamic synchrony.^42^ This suggests absence seizure oscillations emerge as PO thalamus amplifies between-regional feedback connectivity, thereby generating alternating somatosensory and motor system burst firing within a chaotic attractor, in a manner that RT is unable to shut off when *Scn8a* is knocked down. Future work should examine further cell-type specificity and explore additional regulators of this process such as dendritic inhibition and intra-thalamic circuits.^11, 50^

### Multisite optogenetic stimulation reveals a direct model prediction of phase-lag-dependent seizure growth

At the level of LFP amplitudes, our model revealed a simple mechanism for seizure growth: at certain phase-lags between SomL and MotL, input from SomL to MotL causes amplitude growth in MotL, while input from MotL to SomL does not cause amplitude growth in SomL. At other phase-lags, the opposite happens (no amplitude growth in MotL but amplitude growth in SomL) (Fig. 4HJK). We tested this prediction at the level of driving source spiking with optogenetic stimulation of Som:L5 and PreMot:L5 while recording Ecog in both regions (Fig. 7H). Remarkably, as predicted by our model, we found a strong dependence of the measured LFP amplitude in both regions on the time-lag between stimulation of each region (Fig. 7KL and Fig. S8H). Furthermore, when isolating the specific contribution of input from one region to the amplitude of the first SWD-spike in the other region (Fig. 7M), we found a strong anticorrelated response as a function of the stimulation phase-lag (Fig. 7N), as predicted by our model. At certain stimulation phase-lags between Som:L5 and PreMot:L5, input from Som:L5 to PreMot:L5 leads to a maximal response in Mot, while input from PreMot:L5 to Som:L5 leads to a minimal response in Som (Fig. 7P). At other phase-lags, the opposite happens (minimal response in Mot and maximal response in Som).

In summary, our model-guided experiments revealed three related anticorrelated properties of Mot and Som as a function of (stimulation) phase-lags between them: model LFP amplitude growth versus no growth (Fig. 4HK), bursting versus tonic firing (Fig. 6I), and strong versus weak functional connectivity (Fig. 7P). These three properties covary together in one region and are opposite in the other regions. For example, when one region will have LFP amplitude growth, high burst rates, and strong functionally enhanced input connectivity, the other will have the opposite in all 3.

### A common circuit mechanism with general anesthesia

A defining clinical feature of absence seizures is a sudden loss of consciousness that lasts for a typical duration of tens of seconds, followed by an equally sudden restoration of consciousness. Remarkably, there are no overt effects on cognition or awareness just before or just after the seizure. This rapid, specific, and direct transition from consciousness to unconsciousness and back at the beginning and end of the seizure, suggests that the absence seizure network may be a central substrate of consciousness.

Our results indicate that during seizures, this network undergoes disrupted long-range cortico-cortical and cortico-thalamic communication. Specifically, we identify the posterior thalamic nucleus (PO) as modulating feedback connectivity strength between motor and somatosensory cortices. Notably, corticothalamic loops of the kind we identified, particularly those involving higher-order processing rather than first-order sensory processing, have been proposed as substrates of consciousness.^51–54^ This proposal aligns with recent findings that PO regulates cortico-cortical communication by depolarizing apical dendrites of cortical L5 pyramidal neurons and that this process is disrupted under anesthesia.^31^ Such disruption would lead to attenuated signal flow between Mot and Som systems. The overlap between absence seizure networks and anesthesia sensitive circuits suggests a shared mechanism of loss of consciousness: disrupted between-region L5 pyramidal coupling linked to PO dysregulation of feedback connectivity.

Our results may also connect to one framework for thinking about consciousness: the Global Neuronal Workspace (GNW) Theory.^55^ This framework emphasizes the integration of local information processing within a shared larger-scale network through a global broadcasting principle that could include wide-spread communication via distributed networks like motor and somatosensory areas. Our results show that seizures reflect PO-induced hyper-functional connectivity between motor and somatosensory areas, which in turn drives brain dynamics to a pathological low-dimensional chaotic attractor state that would impede global broadcast signals necessary for consciousness in the GNW theory. Similarly, in anesthesia, attenuated cortico-cortical functional connectivity could also impede such global broadcast signaling. Overall, highly regulated cortico-cortical functional connectivity that is neither too large, as in seizures, nor too small, as in anesthesia, may be essential for both global broadcasting and consciousness, as well as possible effective transitioning between brain states underlying consciousness.^56^

### Limitations

Our model, like all models, is an abstraction of more complex dynamics and advances understanding at a specific level of abstraction. We separated signals into discrete bands and the identified model used nonlinear cross-frequency coupling to handle interactions between frequency bands, a result similar to those from nonlinear optics.^57^ Other embeddings like PCA, UMAP, or autoencoders also decompose signals but without constraints on interpretability and are less readily compatible with symbolic equation discovery.^58^ But still, obtaining biological interpretations of individual bands should also be done cautiously. For this reason, we often returned to the SWD-spike as a focal point of analysis. Our model, trained on LFPs, reflects a combination of spiking, synaptic activity, and subthreshold voltage changes.^59^ Also, our model focused strictly on identifying the causes of absence seizure generation. Importantly, it does not model the termination of seizures. We propose the model coefficients represent a snapshot of the more slowly varying parameters that support seizure generation. These slow variations include arousal, attention, sensory input, and neuromodulation, all of which have been shown to impact seizure prevalence.^6, 60–62^ However, each of these processes are highly complex and beyond the scope of this study. Thus, our model is a simplification of underlying influences affecting neuronal dynamics. Nevertheless, by focusing on learning the model from data specifically around the time of seizure onset, we obtained a highly accurate model that captured 60 seizure metrics 3 s into the future seizure from a non-seizure initial state, and yielded many novel experimentally confirmed predictions about the dynamical causes of absence seizures.

## Supplementary Material

1
