# Supplementary material for: Interpretable Machine Learning Identifies an Emergent Absence Seizure Mechanism
Source: bioRxiv. 2026 Sep 16:2025.09.23.678032. Preprint. [Version 2] doi: 10.1101/2025.09.23.678032 (PMC13596142; doi:10.1101/2025.09.23.678032)
Supplement: Supplementary file 1 [file NIHPP2025.09.23.678032V2-supplement-1.pdf]

bioRxiv preprint doi: https://doi.org/10.1101/2025.09.23.678032; this version posted September 16, 2026. The copyright holder for this preprint (which was not certified by peer review) is the author/funder, who has granted bioRxiv a license to display the preprint in perpetuity. It is made available under a CC-BY 4.0 International license.

Supplemental Figures

bioRxiv preprint doi: https://doi.org/10.1101/2025.09.23.678032; this version posted September 16, 2026. The copyright holder for this preprint (which was not certified by peer review) is the author/funder, who has granted bioRxiv a license to display the preprint in perpetuity. It is made available under a CC-BY 4.0 International license.

Supplemental Figure1

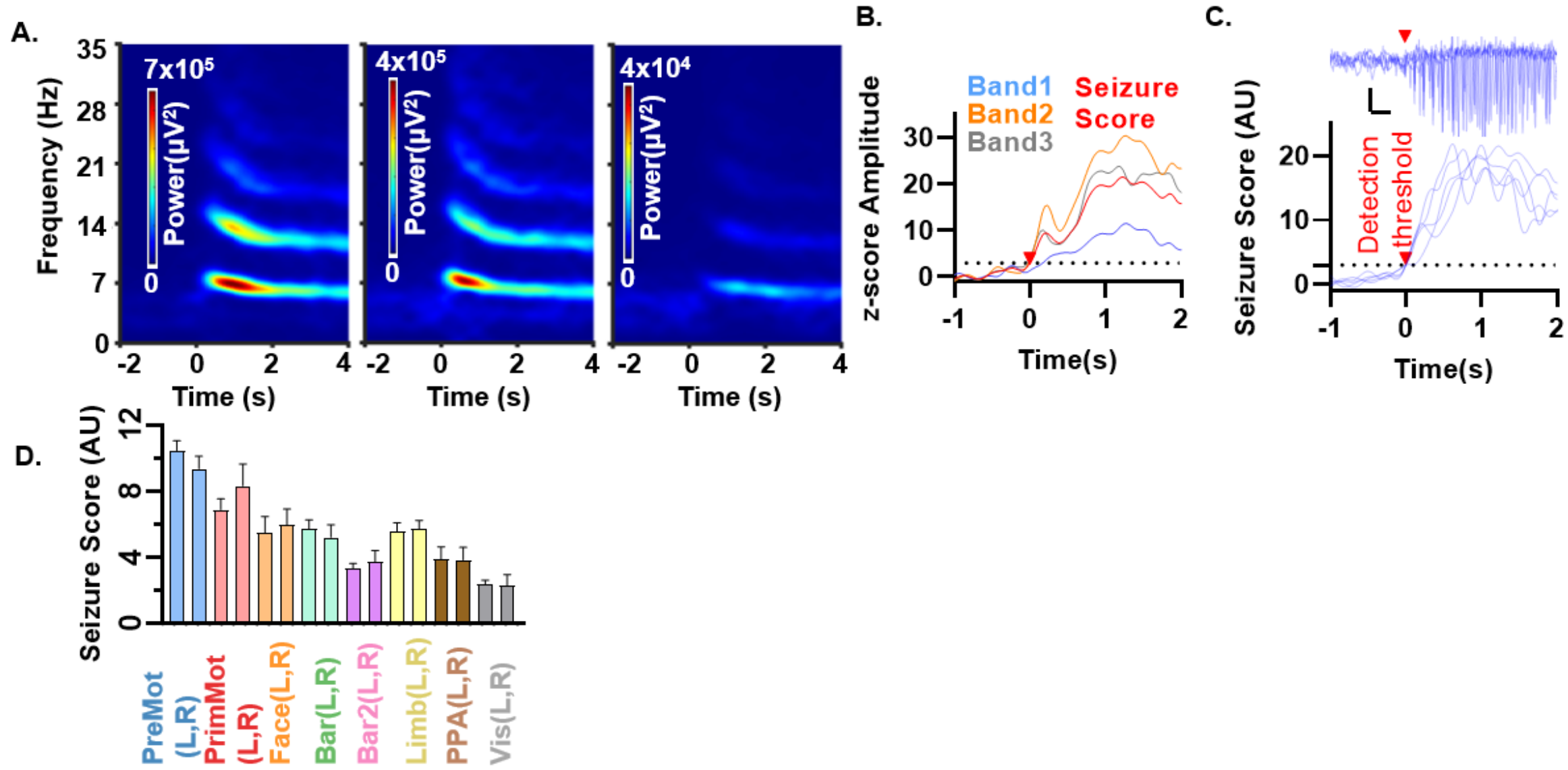

Figure S1: Seizure amplitude dynamics quantified absence seizure power

(A) Time-frequency spectrograms of Ecog signals for different brain regions in Premotor Left (PreMotL), Face Left (FaceL), and Visual Left (VisL) showing increases in power in bands that are harmonics of the fundamental frequency.

(B) z-score amplitude for an example PreMot band 1, 2, and 3 and the seizure score (the average of the z-score of the amplitude envelope of each of the 3 bands). Note band 1 exhibits the smallest change in amplitude.

(C) Five PreMotL seizures (top) aligned to crossing seizure score of 3 (red arrow), the detection threshold of seizures. Scale bar 200 ms and 2 mV. Onset times defined as last time point of crossing seizure score of 1 prior to passing the detection threshold.

(D) Average and SEM seizure score for 3 s as in C after onset at the leading site across all recorded regions.

bioRxiv preprint doi: https://doi.org/10.1101/2025.09.23.678032; this version posted September 16, 2026. The copyright holder for this preprint (which was not certified by peer review) is the author/funder, who has granted bioRxiv a license to display the preprint in perpetuity. It is made available under a CC-BY 4.0 International license.

Supplemental Figure2

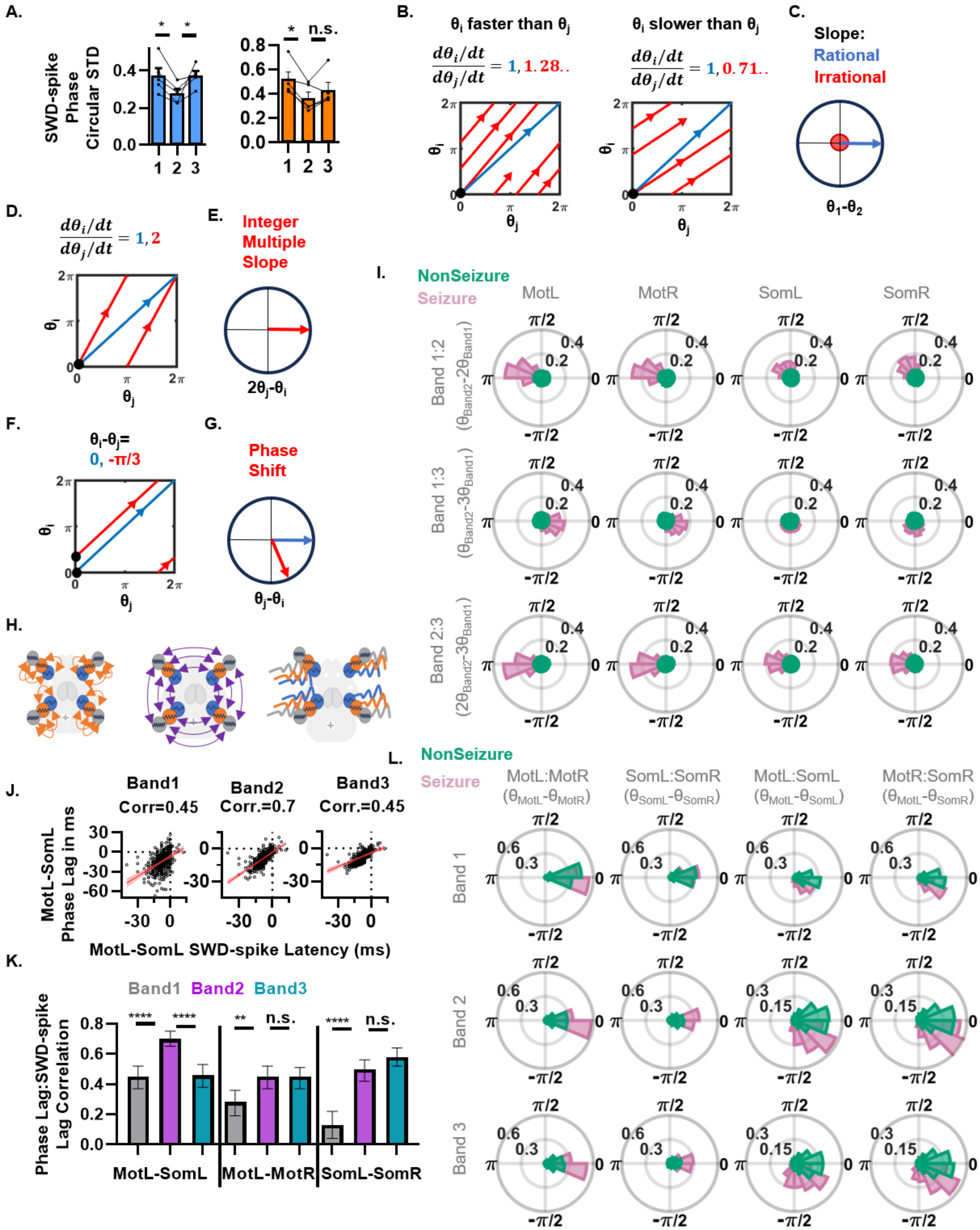

bioRxiv preprint doi: https://doi.org/10.1101/2025.09.23.678032; this version posted September 16, 2026. The copyright holder for this preprint (which was not certified by peer review) is the author/funder, who has granted bioRxiv a license to display the preprint in perpetuity. It is made available under a CC-BY 4.0 International license.

Figure S2.

(A) Circular standard deviation of the phase of the SWD-spike showing SWD-spikes are more precisely locked to band 2 than band 1 in Mot (blue) and Som (orange). Paired t-test.

(B-F) Schematics showing several theoretical phase relationships on toroidal manifolds. (B) Effects of different phase velocity ratios on theoretical trajectories. 1:1 ratio phase velocities (blue) return to same trajectory while irrational frequencies with $\theta_i > \theta_j$ (red trajectory B, left) and $\theta_i < \theta_j$ (red trajectory B, right) drift filling the torus. (C) Phase difference histogram quantifies mean phase-lag (m) and phase coherence (r) of the corresponding relationships as in B with no mean phase-lag and no phase coherence when slopes are irrational (red) and strong coherence (r) and phase-lag (m) when differences are locked at a ratio of 1 with zero phase-lag (blue). (D) Rational slopes on the torus (here 2, red) also return to the same trajectory similar to slope 1 trajectories (blue). (E) Phase difference histogram quantifies phase-lag and coherence when the slower phase is multiplied by the ratio of the two velocities. (F) Oscillation phase-lags on the torus of integer slope trajectories alter the intercept ($-\pi/3$ red vs 0 rad blue) but not slope of the trajectory. (G) Phase histogram quantifies change in phase-lag as a shift in the angle on the histogram.

(H) Schematic of the between-band (left) and between-region (middle) relationships and amplitude trajectories (right) used in defining the state space of absence seizures.

(I) Phase-difference histograms across all between-band relationships in (H). Each of the 12 histograms has a seizure specific coherence and phase-lag that varies substantially across bands and regions.

(J) Scatter plots of individual SWD-spike time-lags between MotL and SomL and the observed phase-lag converted to ms as in main text. Note high correlation with between band 2 phase-lags and SWD-spike lags.

(K) Correlations as in J across different regions. Note band 2 is higher than band 1 across all connections and higher than band 3 in the MotL-SomL.

(L) Phase-difference histograms across all between-regional relationships in (H). Each of the 12 histograms has a seizure specific coherence and phase-lag that varies substantially across bands and regions.

bioRxiv preprint doi: https://doi.org/10.1101/2025.09.23.678032; this version posted September 16, 2026. The copyright holder for this preprint (which was not certified by peer review) is the author/funder, who has granted bioRxiv a license to display the preprint in perpetuity. It is made available under a CC-BY 4.0 International license.

1218 Supplemental Figure 3

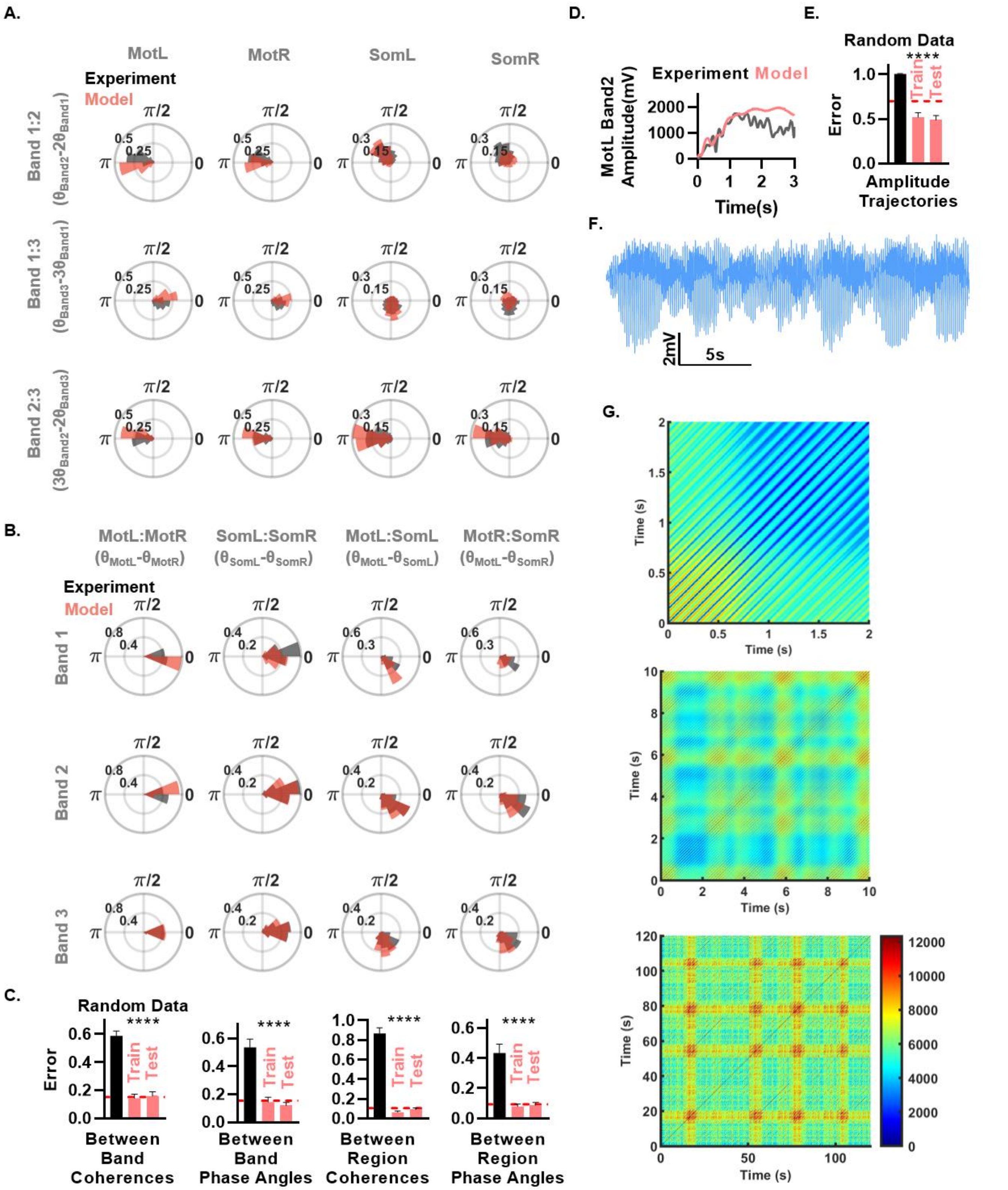

bioRxiv preprint doi: https://doi.org/10.1101/2025.09.23.678032; this version posted September 16, 2026. The copyright holder for this preprint (which was not certified by peer review) is the author/funder, who has granted bioRxiv a license to display the preprint in perpetuity. It is made available under a CC-BY 4.0 International license.

Figure S3: The model recapitulated evolution to the seizure state space.

(A and B) Phase-histograms of experiment and model from the same initial conditions recapitulate phase-lags and coherence across bands (A) and regions (B).

(C) Model error on coherence and phase-lag attractor space metrics. Red dashed line indicates the error metric computed between different experimental mice as if they were the results of simulation. Asterisks indicate significance relative to time shuffled data, all Train and Test $p<0.0001$. Train data corresponds to error of simulated data when the 400 ms before seizure onset to 800 ms after seizure onset were included in regression steps and used for performance on state-space error metrics used in library selection steps. Test data indicate novel seizures not included in regression or in library selection.

(D) Example MotL band 2 amplitude trajectory showing initial tracking of the trajectory over ~1s.

(E) Model error in tracking amplitude trajectories. Asterisks indicate significance relative to time shuffled data. Test and train as above, both $p<0.0001$ vs time shuffled data. Red dashed line as above.

(F) MotL voltage trace over first 25 s of a 1200 s long simulation. Note variation in amplitude over seconds long time scales.

(G) Recurrence plots of a simulation during the last 120 s of the above simulation. Color indicates Euclidian distance between states of the entire system at indicated time-lags. Scale bar in µV. Note the diagonal unity line at 0 time-lag indicates the 0 µV distance from a state to itself. Note the blue parallel diagonal lines at temporal lags occurring at multiples of the SWD interval (~133 ms), indicate that successive SWDs are similar, but not identical as amplitudes and phases subtly and chaotically vary from cycle to cycle across bands and regions. (Middle) Distances between state pairs over a longer range of temporal lags indicates aperiodic dynamics, with the system often coming near to previous states, but never identically back to a previous state. Note the strong blue diagonals at short temporal lags, even during periods of low similarity (yellow) indicating SWDs occurring in these states. (Bottom) Longer view of the last 120 s shows strong and transient aperiodic departures from previous states (red regions).

bioRxiv preprint doi: https://doi.org/10.1101/2025.09.23.678032; this version posted September 16, 2026. The copyright holder for this preprint (which was not certified by peer review) is the author/funder, who has granted bioRxiv a license to display the preprint in perpetuity. It is made available under a CC-BY 4.0 International license.

1245

Supplemental Figure 4

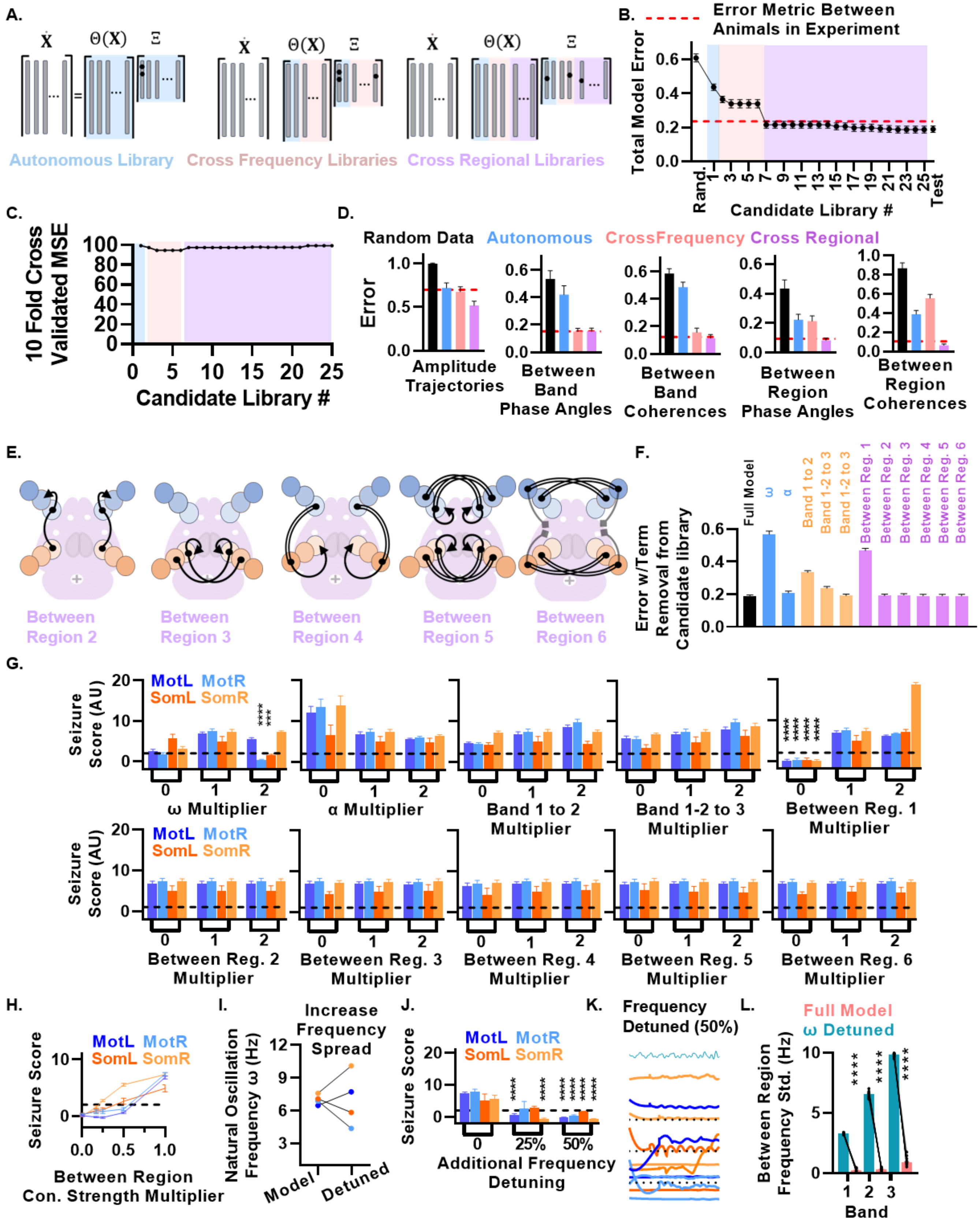

bioRxiv preprint doi: https://doi.org/10.1101/2025.09.23.678032; this version posted September 16, 2026. The copyright holder for this preprint (which was not certified by peer review) is the author/funder, who has granted bioRxiv a license to display the preprint in perpetuity. It is made available under a CC-BY 4.0 International license.

Figure S4: The model selection approach enables interpretable model performance metrics and model function.

(A) Schematic representation of the modified SINDy (Sparse Identification of Nonlinear Dynamics) approach for identifying dynamical models from experimental data using candidate function library ($\Theta(\mathbf{X})$) built from various expansion steps to identify term coefficients ($\boldsymbol{\Xi}$) that best describe the system's dynamics ($\dot{\mathbf{X}}$). Broad classes of $\Theta(\mathbf{X})$ at the level of autonomous functions, between-band functions, and between-regional functions shown in respective colors highlighting the appending of more complex library terms to those selected at lower complexity levels.

(B) Total error metric (Supplementary Equation 9) for each tested library as defined in table S1. Red dashed line indicates the same error metric computed for experimental data between mice. Test indicates performance on held-out seizure data not included in regression or library selection steps.

(C) Cross-validated mean square error for single step regression across libraries showing no basis for model selection between libraries which show improvement in (B).

(D) Model error for amplitude trajectory, and mean phase angles and consistency between-band, and between-regions at different broad levels of candidate library model complexity showing improvement in specific metrics corresponding to library type (Autonomous libraries, between-band libraries, and between-region libraries). Red dashed line indicates same metrics computed between animals.

(E) Schematics of coupling functions not shown in Fig. 4B.

(F) The effect of removal of each function class on total model error when that function class is removed from the final candidate library. Note that model error increases most with removal of ω (from library 1), between-band 1-2 coupling (library 2), and between-region (library) coupling. Note the negligible effect of between-region function classes 2-6 (libraries 15, 17, 20-22), function schematics in (E).

(G) Effect of multiplying each function class term coefficients by 0 (0), doubling them (2), and the learned model (1). Note only removal of between-regional coupling abolishes seizures. Also note that modification of other between-region function classes has a negligible impact on seizure score. Dashed line indicates seizure score of 2, i.e. amplitude growth below 2 z-score from starting condition.

(H) Sweep weights of between-region within band coupling reveal total abolition of seizures at a 6-fold reduction.

(I) Intrinsic frequencies ω in model for band 1 and 50% maximal detuning i.e. with oscillators detuned 50%, 25%, -25%, and -50% of the learned ω. Bands 2 and 3 set at corresponding multiples of their band 1 frequency.

(J) Seizure score vs maximum frequency detuning. Asterisks indicate significant reduction below seizure score of 2, i.e. amplitude growth below 2 z-score from starting condition. Comparison via a 1 sample t-test. ****$p<0.0001$.

(K) Representative voltage traces and oscillation frequency in each region for model with detuning as in I.

(L) Standard deviation of frequency between regions as in Fig. 4E and G. n=25 simulations with initial conditions from 5 mice. Asterisks indicate significance via two tailed t-test. ****$p<0.0001$

bioRxiv preprint doi: https://doi.org/10.1101/2025.09.23.678032; this version posted September 16, 2026. The copyright holder for this preprint (which was not certified by peer review) is the author/funder, who has granted bioRxiv a license to display the preprint in perpetuity. It is made available under a CC-BY 4.0 International license.

Supplemental Figure 5

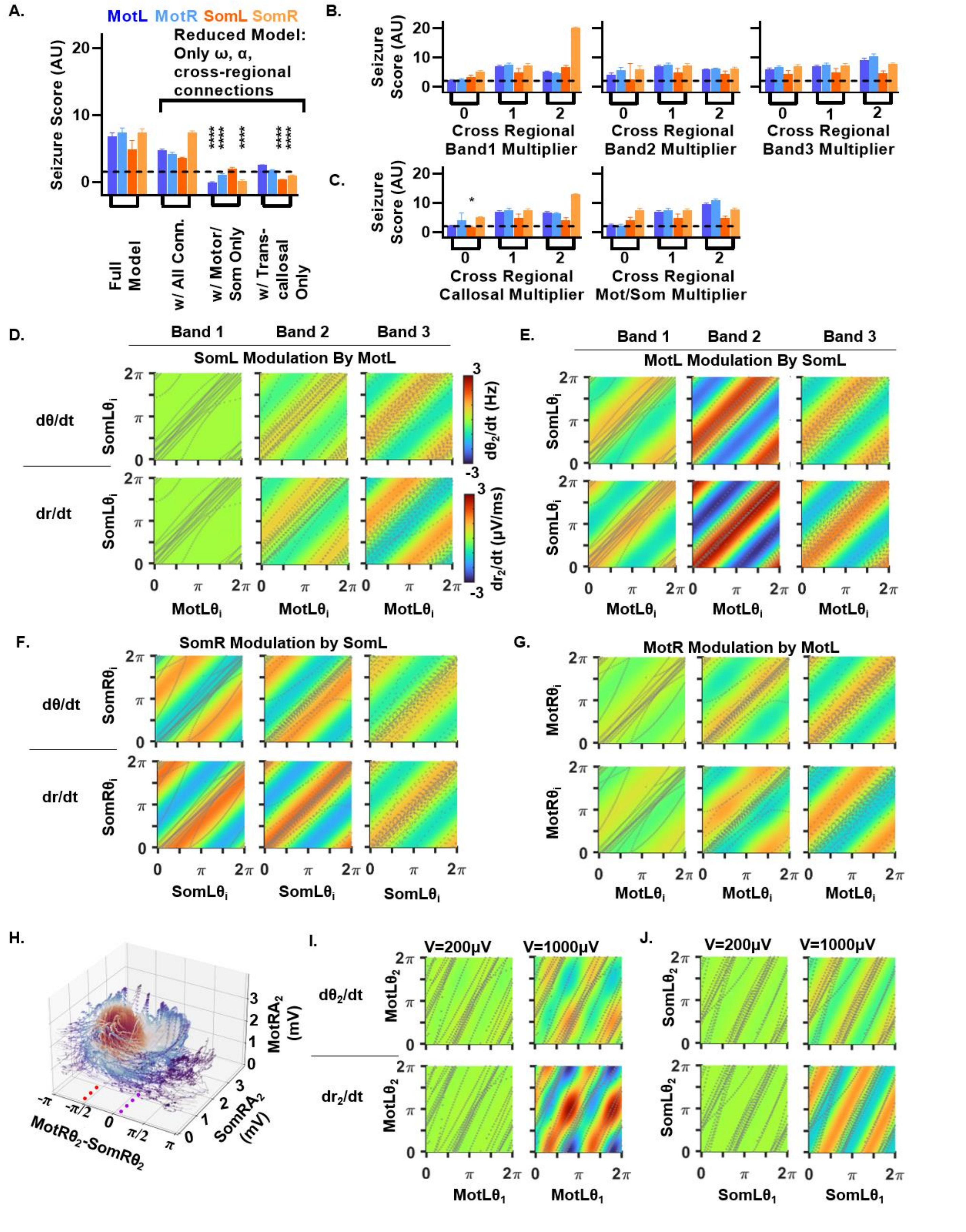

bioRxiv preprint doi: https://doi.org/10.1101/2025.09.23.678032; this version posted September 16, 2026. The copyright holder for this preprint (which was not certified by peer review) is the author/funder, who has granted bioRxiv a license to display the preprint in perpetuity. It is made available under a CC-BY 4.0 International license.

Figure S5: Between-region coupling functions enabled interpretation of drives behind dynamics.
(A) Effect of reducing the model to include only the autonomous and stable oscillator terms (intrinsic frequencies ω and limit cycle α) and the seizure driving between-region coupling shown schematically in Fig. 4B. Note robust seizure generation comparable to full model. Further reducing the model by removing either Mot to Som and Som to Mot or by removing all transcallosal coupling reduces but does not abolish seizures. Asterisks indicate significant reduction below seizure score of 2, i.e. amplitude growth below 2 z-score from starting condition. Comparison via a 1 sample t-test. ****$p<0.0001$
(B) In the full model, removing or doubling the seizure-driving coupling terms in each band individually does not abolish seizures in isolation.
(C) In the full model, removing or doubling either Mot to Som and Som to Mot or by removing all transcallosal coupling impairs but does not abolish seizures as in (A).
(D) Phase and amplitude coupling functions for band 1, 2, and 3 for SomL modulation by MotL. Note the lack of Mot to Som coupling in band 1 while band 2 and 3 are stronger and both exhibit a relative phase offset in amplitude modulation toward $-\pi/2$ rad. Gray dots indicate a typical seizure trajectory.
(E) Phase and amplitude coupling functions for band 1, 2, and 3 for MotL modulation by SomL. Note the strong coupling in band 1 in contrast to lack of coupling in band 1 in D. Also note that all coupling functions in E follow a similar pattern and amplitude modulation is nearly symmetrical through 0 phase offset across all bands.
(F) Phase and amplitude coupling functions for band 1, 2, and 3 for SomR modulation by SomL. Note the strong coupling in band 1 in contrast to D. Also note that all coupling functions in F follow a similar pattern and amplitude modulation is nearly symmetrical through 0 phase offset across all bands.
(G) Phase and amplitude coupling functions for band 1, 2, and 3 for MotR modulation by MotL.
(H) Examination of band 2 trajectories along the chaotic attractor during long simulations. Dynamics of MotR-SomR coupling. Compare to similar coupling in Fig. 4M.
(I and J) Coupling functions for band 1 to 2 in MotL (I) and SomL (J). Note the amplitude dependence of the coupling functions lead to negligible modulation at 200 µV but modulation comparable to between-regional connections at 1000 µV, indicating why band 1-2 phase relationships are only observed during seizure.

bioRxiv preprint doi: https://doi.org/10.1101/2025.09.23.678032; this version posted September 16, 2026. The copyright holder for this preprint (which was not certified by peer review) is the author/funder, who has granted bioRxiv a license to display the preprint in perpetuity. It is made available under a CC-BY 4.0 International license.

Supplemental Figure 6

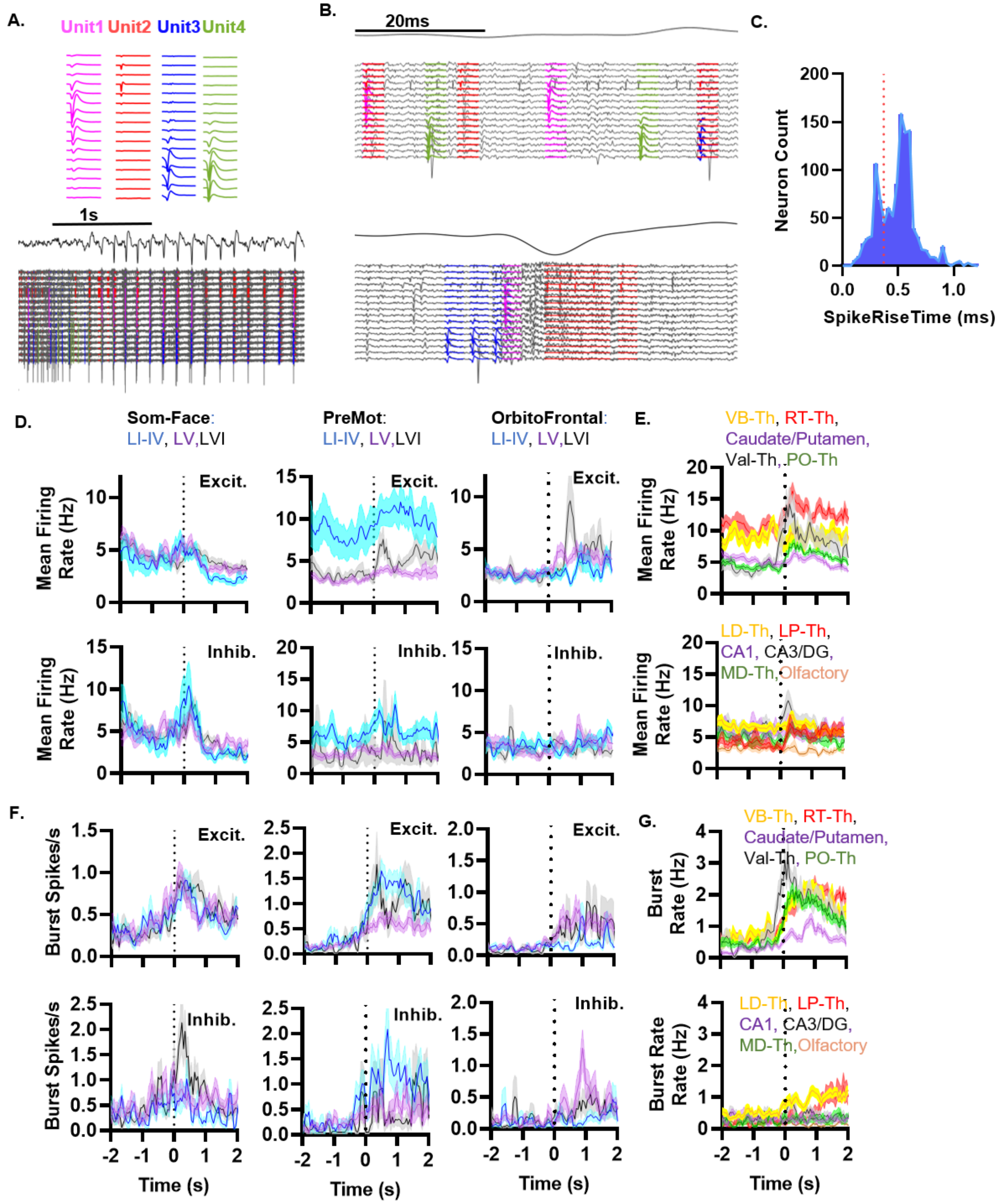

Figure S6: Spike Sorting and basic spike rate parameters across recorded populations. (A) Example average waveforms of 4 identified single units in PO on the same recording channels (top) and example

bioRxiv preprint doi: https://doi.org/10.1101/2025.09.23.678032; this version posted September 16, 2026. The copyright holder for this preprint (which was not certified by peer review) is the author/funder, who has granted bioRxiv a license to display the preprint in perpetuity. It is made available under a CC-BY 4.0 International license.

seizure Ecog trace (middle) and example recording channels with units marked by color. Note dispersed firing just before seizure and spikes aligned to SWD-spike with near total silence between SWDs.
(B) Expanded view of spiking outside of seizure in A. Expanded view of spiking during the SWD-spike and relative lack of spiking outside the SWD-spike (bottom). Also note while there is period of no detected units spanning 3.7 ms during the spike, there is prominent detection of multiple high-frequency spikes (i.e. bursting) of stable waveform during the SWD.
(C) Histogram of single unit spike widths in PreMot and Som cortex used to differentiate excitatory (right of the dashed line) from inhibitory (left of the dashed line) units.
(D and E) Mean firing rates across populations leading up to and during absence seizures. Note the lack of large changes in mean firing within any population with the largest percent change being a 2.8 fold increase in Val thalamus (E). Note the *decreased* firing in Som:L5 and L6 during seizures.
(F and G) Count of bursts/s across regions. Bursts defined as >40Hz interval preceded by <10Hz interval. Note the >4-fold increases in many regions. Note also the delayed onset or absence of burst firing in orbitofrontal cortex and LD, LP, MD thalamus as well as CA1, CA3/DG and olfactory areas.

bioRxiv preprint doi: https://doi.org/10.1101/2025.09.23.678032; this version posted September 16, 2026. The copyright holder for this preprint (which was not certified by peer review) is the author/funder, who has granted bioRxiv a license to display the preprint in perpetuity. It is made available under a CC-BY 4.0 International license.

Supplemental Figure 7

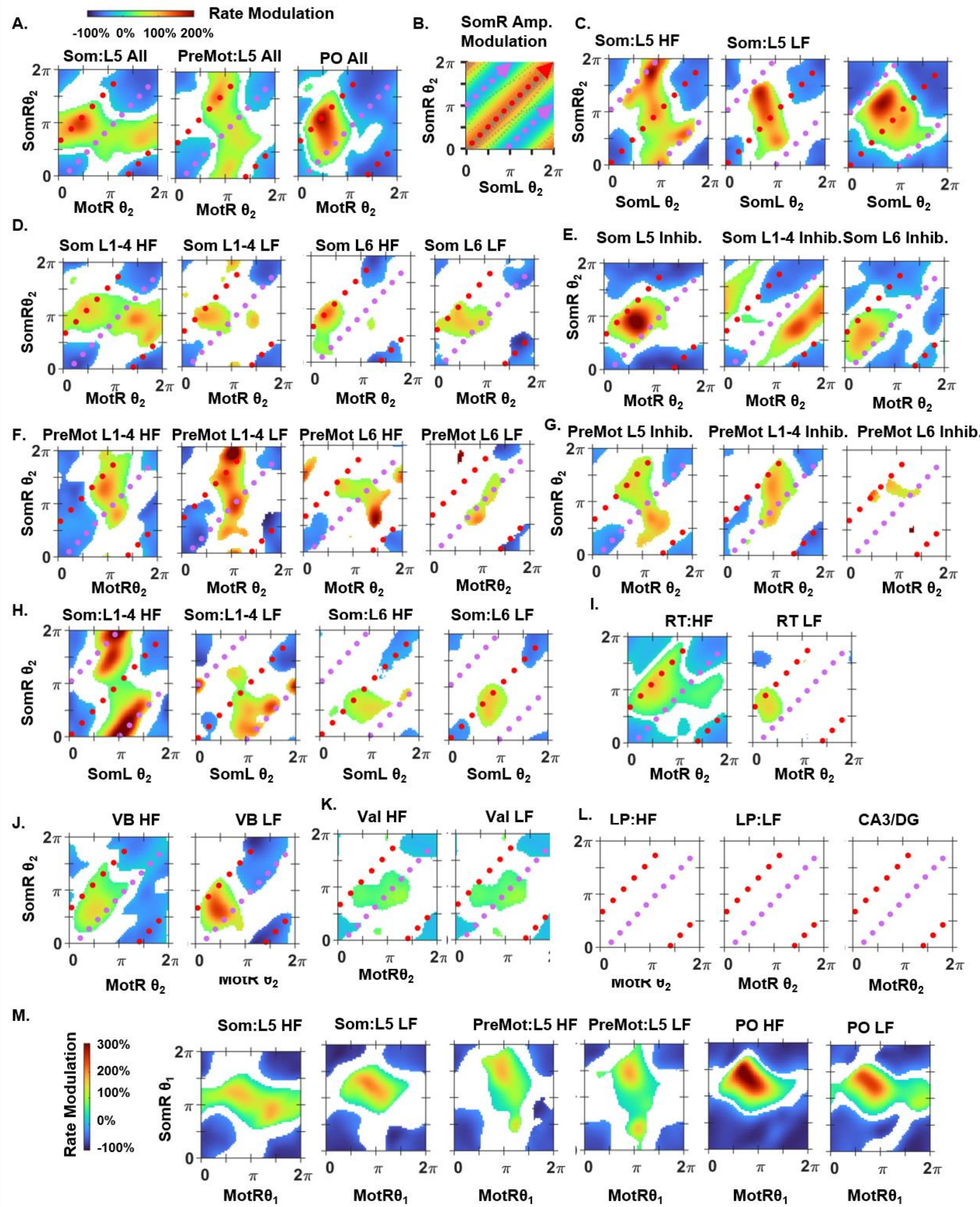

bioRxiv preprint doi: https://doi.org/10.1101/2025.09.23.678032; this version posted September 16, 2026. The copyright holder for this preprint (which was not certified by peer review) is the author/funder, who has granted bioRxiv a license to display the preprint in perpetuity. It is made available under a CC-BY 4.0 International license.

Figure S7: Model coupling functions inform firing pattern across different populations

(A) Rate modulation of all spiking (burst and tonic firing) in populations from Fig. 6.

(B) Coupling function for SomL modulation of amplitude in SomR band 2. Peak amplitude growth marked by red and decay by purple trajectories.

(C) Firing along trajectories in (B). Som:L5 high-frequency (HF) firing (left) follows a similar pattern similar pattern as shown in Fig. 6 with increases (red region) and decreases (blue region) along the amplitude growth trajectory (red trajectory) for the SomL to SomR connection in the model and an increase in HF firing without accompanying silencing along the amplitude decay trajectory (purple trajectory). Low-frequency (LF) firing (middle) increased and decreased along the growth trajectory and was not modulated along the decay trajectory (purple trajectory). PO HF firing (right) showed a similar pattern as PO in Fig. 6 with rate modulation increasing (red region) and then decreasing (blue region) along the growth trajectory while the decay trajectory (purple trajectory) was not strongly modulated.

(D) Som:L1-4 and Som:L6 followed similar but weaker pattern of modulation as Som:L5 observed in Fig. 6E.

(E) Som Inhibitory units followed distinct patterns from excitatory neurons across layers. Som:L5 inhibition (left) was strongly positively modulated (red region), occupying the region between trajectories and showed silencing (blue regions) along all trajectories. Som:L6 (right) followed a weaker but similar pattern to Som:L5. Som:L1-4 (middle) inhibitory firing followed a distinct pattern from both Som:L1-4 excitatory firing (D, left) and inhibitory firing in Som:L5 and L6. Note the Som:L1-4 inhibitory unit silencing along both trajectories and strong activation outside of the seizure state space.

(F) PreMot:L1-4 and L6 followed more distinct patterns from PreMot:L5 observed in Fig. 6G. Notably, L1-4 exhibited burst behavior along both trajectories while L6 was weakly modulated.

(G) PreMot:L5 inhibition (left) peaked along Som growth trajectories and was silenced during Mot Growth trajectories while PreMot:L1-4 firing increased and then was silenced along both trajectories. PreMot:L6 was weakly modulated.

(H) Som:L1-4 HF firing was weakly activated (green region) and then silenced (blue region) along the SomL-SomR (coupling function shown in B) growth trajectory (red trajectory) and LF firing was strongly activated along the amplitude decay trajectory (purple trajectory). Som:L6 HF and LF was moderately activated and silenced along the growth trajectory.

(I) The thalamic reticular nucleus (RT) exhibited increased HF firing followed by silencing along the Som growth trajectory (red trajectory) and modest HF firing along the Mot growth trajectory (purple trajectory. Note however RT contains neurons associated with diverse brain systems including motor, sensory and limbic systems complicating interpretation.

(F and K) Ventrobasal thalamus (VB) showed burst firing between trajectories, similar to Som:L5 and Som:L6 inhibition suggesting a role in feedforward inhibition. Note overall weaker association with the torus than PO (Fig. 6J). (K) Ventral anterolateral thalamus (Val) showed bursting along the Mot growth trajectory.

(L) There are regions with firing uninformed by the phase torus. Lateral posterior (LP) thalamic nucleus, a higher order vision related nucleus, and CA3/DG exhibited no modulation on the band 2 Mot-Som phase torus.

(M) Band 1 Mot-Som phase torus exhibits modulation of Som:L5, Mot:L5 and PO firing. Modulation of Som:L5 indicates it predominantly represents coupling with band 2 due to the presence of multiple peaks, as predicted by the lack of band 1 coupling from Mot to Som (Fig. S5D).

bioRxiv preprint doi: https://doi.org/10.1101/2025.09.23.678032; this version posted September 16, 2026. The copyright holder for this preprint (which was not certified by peer review) is the author/funder, who has granted bioRxiv a license to display the preprint in perpetuity. It is made available under a CC-BY 4.0 International license.

Supplemental Figure 8

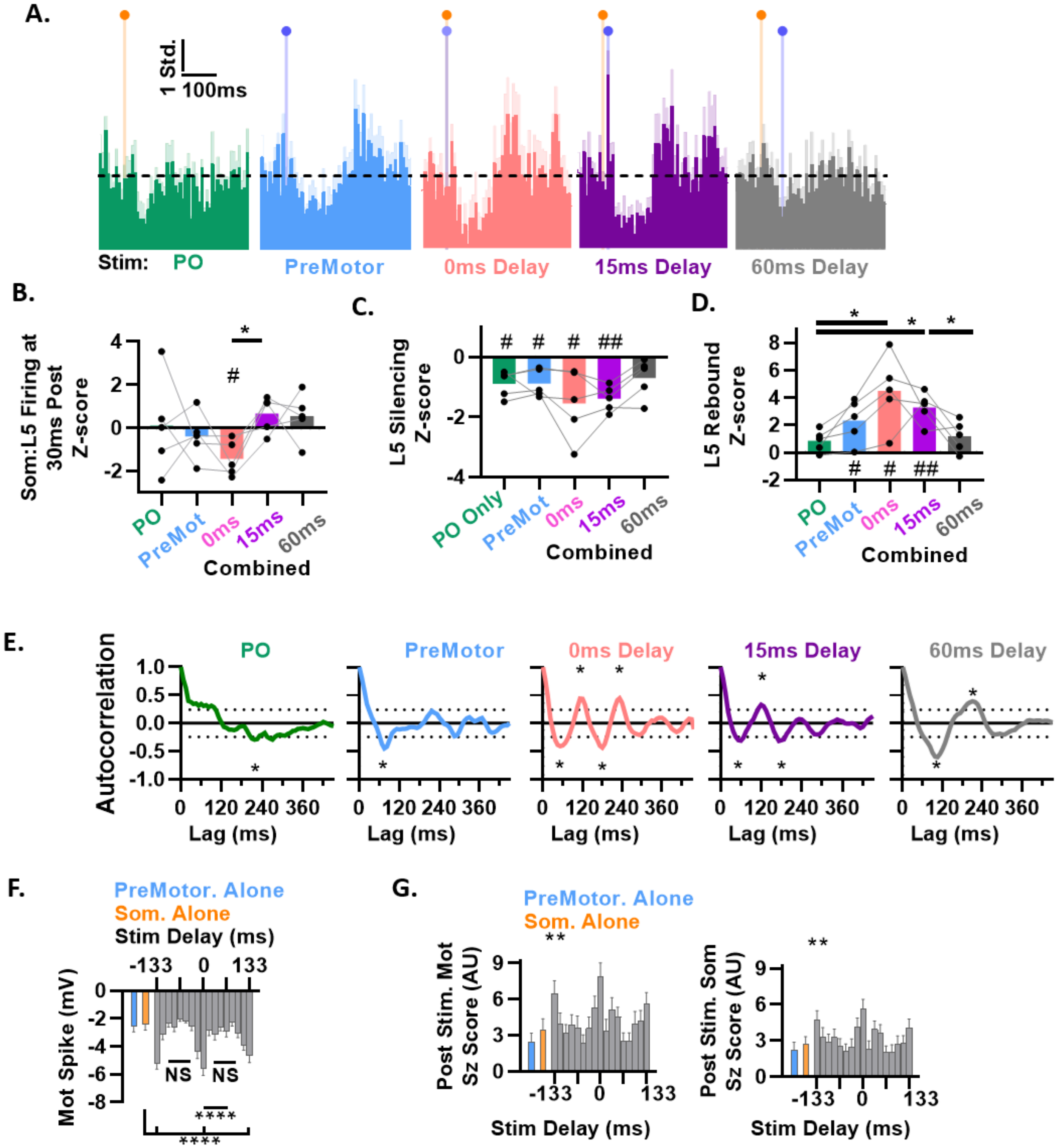

Figure S8: Combined optogenetic stimulations induce seizure rhythms

(A) Extended time view of PSTH plots from Fig. 7F.

bioRxiv preprint doi: https://doi.org/10.1101/2025.09.23.678032; this version posted September 16, 2026. The copyright holder for this preprint (which was not certified by peer review) is the author/funder, who has granted bioRxiv a license to display the preprint in perpetuity. It is made available under a CC-BY 4.0 International license.

(B) Firing of Som:L5 neurons at 30 ms after initial stimulation onset. Note robust silencing with the 0 ms delay stim, consistent with 0 ms delays promoting spiking suppression after the (nonsignificant) peak occurring at 15 ms in Fig. 7G. In contrast, the 15 ms delay, which caused a large increase in firing at the 15 ms delay, is significantly higher than the 0 ms delay at 30 ms post initial stim of PO. PO alone, PreMot alone, or 60 ms delay do not cause silencing at this early timepoint. # indicate one-way t-test vs 0, asterisk indicate between group comparisons via paired t-test).
(C) All stimulations except 60 ms delay produce Som:L5 silencing (average at 45:90 ms post initial stimulation). # indicate one-way t-test vs 0, asterisk indicate between group comparisons via paired t-test).
(D) PreMot alone, 0 ms delay, and 15 ms delay produced a rebound increase in Som:L5 firing. 0 ms and 15 ms were elevated relative to PO or 60 ms stimulation while 0 ms was additionally elevated relative to PreMot. # indicate one-way t-test vs 0, asterisk indicate between group comparisons via paired t-test).
(E) Autocorrelation of the average PSTH following the silencing period. 95% confidence bounds for the were computed using Bartlett's formula under a white-noise null. Only the 0 and 15 ms delay produced seizure interval oscillations (~120 ms). The 60 ms delay produced a slow oscillation near 2x the seizure interval. Asterisk indicate >two consecutive points above the 95% confidence bounds.
(F) Average minimum spike amplitude across 10 trials under each phase-lag condition for Mot as in Fig. 7L. Lines show pairwise t-tests.
(G) Seizure score in the 1 second after stimulation end. Asterisks indicate significance by ANOVA. **p<0.01.

Supplemental Methods

Animals

Heterozygous loss of function mutation in *Scn8a* (*Scn8a*+/-) mice were purchased from Jackson laboratory (C3HeB/FeJ-*Scn8a*med/J, Kohrman et al., 1996, Stock#: 003798) and maintained on the C3HeB/FeJ background. For optogenetic experiments, homozygous *Thy1-mhChr2-YFP* (B6.Cg-Tg(Thy1-COP4/EYFP)18Gfng/J, Stock# 007612) were purchased from Jackson laboratory and crossed with *Scn8a*+/- mice to generate *Scn8a*+/-; *Thy1-mhChR2-YFP*+/- mice. Mice were genotyped at postnatal day 21 through the Transnetyx corporation. Mice were maintained on a 12 hr light/dark cycle and male and female mice were used in all experiments. Experiments were approved by the Stanford Administrative Panel on Laboratory Animal Care (APLAC, Protocol #12363) and in accordance with the National Institute of Health guidelines.

Stereotaxic surgeries

For all surgeries mice were p56-90. Mice were anesthetized with an isoflurane oxygen mixture at 4% for induction of anesthesia and 1-2% for maintenance. Subcutaneous Rimadyl (5mg/kg) was administered after induction and for three days following surgery. Mice in Fig. 1 were implanted with 16 gold electrodes (1.27mm, Mill-Max Manufacturing Corp) in contact with the dura in the locations shown in Fig 1A. Coordinates relative to Bregma for electrodes following the numbering in Fig. 1A in mm are; (1,2): 2.68 AP, ±1 ML, (3,4): 1.68 AP, ±2 ML, (5,6): 0.3 AP, ±3.5 ML, (7,8): -0.82 AP, ±3.5 ML, (9,10): -1.82 AP, ±3.5 ML, (11,12): -0.82 AP, ±1.5 ML, (13,14): -1.82 AP, ±1.5ML, (15,16): -2.8 AP, ±2.5 ML. All Ecog signals throughout the manuscript were referenced to a stainless steel Ecog screw (J.I Morris Company FF00CE125) implanted in the skull above the

bioRxiv preprint doi: https://doi.org/10.1101/2025.09.23.678032; this version posted September 16, 2026. The copyright holder for this preprint (which was not certified by peer review) is the author/funder, who has granted bioRxiv a license to display the preprint in perpetuity. It is made available under a CC-BY 4.0 International license.

cerebellum. Mice were also implanted with a custom designed head bar (eMachineShop). Headcaps were constructed using dental cement (Metabond).

For Ecog and Neuropixels recordings with two site optogenetic stimulation of premotor cortex and PO with combined Neuropixels recording from somatosensory cortex *Scn8a+/-; Thy1-mhChR2-YFP*+/- mice were used. The red shifted opsin ChrimsonR (pAAV-Syn-ChrimsonR-tdT (AAV1), 1x10^{13} vg/ml) was delivered to PO as described previously at coordinates (-2.06AP, +1.25ML,-3.2DV).[63] During optogenetic experiments an optic fiber was connected to the optic fiber implanted on the mouse. Blue and yellow laser light were delivered by OEM Laster Systems 473 nm and Cobolt Mambo 594 nm lasers. Pulses were 5 ms wide and laser power titrated to generate small local LFP responses in the target region. Pilot experiments found ChrimsonR virus generated seizures even at very low light stimulation intensity, so we diluted 10x in saline. Injection volume was 400 nL delivered at a rate of 50 nL/min. An optical fiber (200 µm, 0.48-NA, 1.25 mm-diameter stainless steel ferrule, Doric Lenses) was then implanted 500 µm above the injection site and a separate fiber in L5 of premotor cortex (coordinates +2.68AP,+1.5ML,-0.5DV). Gold electrodes as above were placed adjacent to the optical fiber in contact with the dura at (2.68AP, +1.0 ML) and two additional electrodes were placed on the contralateral side (coordinates: +2.68AP, -1ML) and (coordinates -0.0 AP, -4ML). Future Neuropixels recording site was marked (coordinates: 0 AP, 4ML). Mice were allowed to express the virus for a minimum of 3 weeks before recording. For Ecog and dual site optogenetics stimulation of premotor and somatosensory cortex *Scn8a+/-; Thy1-mhChR2-YFP*+/- were used. Two optical fibers as above were implanted at coordinates (+2.68, 1.5 and 0, 3.5) with adjacent gold pins at (+2.68AP,1ML and 0AP, 4.0ML). Optogenetic stimulations were excluded from analysis if preceded by a spontaneous seizure.

For acute Neuropixels recordings, an initial surgery implanting a headbar and reference screw were placed as in the Ecog surgeries. The surface of the skull was marked for future craniotomy sites at sites 1: 2.68 AP, 1.5ML; 2: -0.78AP, 4ML; and 3 -2.06AP,1.5ML and small ridge were constructed to create a well for recording. A stainless-steel screw for Ecog recording and additional headcap stability was implanted in the contralateral somatosensory cortex was implanted at -0.78A/D, -4ML. Following one week of recovery, mice were trained for wheel running as in Ecog mice. On the day of recordings, mice were briefly anesthetized, and 3 craniotomies were performed at the marked locations, creating ~0.5 mm diameter craniotomies. Craniotomies were filled with DOWSIL silicon gel (DOW) and covered by KWIK-SIL silicone elastomer (World Precision Instruments).

Electrocorticogram and Neuropixels recordings

For 16 channel Ecog recording, animals were allowed to recover for 1 week and then were acclimated to head fixed wheel running over the course of 1 week. Ecog recordings were performed with head fixed freely running mice in a dark and sound insulated chamber for 1 hour of Ecog recording. For mice with viral injections, three weeks were allowed for virus expression prior to recording. The Ecog electrodes and ground/reference were connected to an Intan headstage (RHD 2132) and OpenEphys acquisition board and sampled at 30 kHz. Data were recorded at 30 kHz using the OpenEphys software and recording system and down sampled to 500 Hz for analysis.[64]

bioRxiv preprint doi: https://doi.org/10.1101/2025.09.23.678032; this version posted September 16, 2026. The copyright holder for this preprint (which was not certified by peer review) is the author/funder, who has granted bioRxiv a license to display the preprint in perpetuity. It is made available under a CC-BY 4.0 International license.

Neuropixels 1.0 probes were coated in DiD, DiI, or DiO dyes prior to insertion. 12-16 hours after craniotomy surgeries, mice were head fixed and the silicon elastomer was removed. Craniotomy sites were washed with saline and surface moisture was wicked away. 3 probes were inserted at an angle of 18 degrees (site 1), 45 degrees (site2), and 0 degrees (site 3) from vertical. Craniotomies were then filled with silicone oil. Probes were intermittently advanced using Sutter MP-285 manipulators at a rate of ~10 µm per second with over the course of 5 minutes. Insertion continued until 384 µm was traversed along the insertion axes. Zero was marked as the measure of the surface of the dura where the 384th electrode contacted conductive tissue at the surface of the brain. Probe ground and reference were shorted and referenced to the cerebellar Ecog screw. Acquisition utilized a National Instruments PXIe-1071 Chassis and PXIe_1000 base station card and recordings were performed with OpenEphys software. Voltages were sampled at 30 kHz and filtered between 300 Hz to 10 kHz for spike band and voltages were sampled at 2500 Hz for LFP band information.

Probes were allowed to rest for 15 minutes prior to the start of acquisition and 3 bouts of recordings of 15 minutes were acquired. Seizures were selected as above for Ecog recordings from the final 15 minute recording bout and additional seizures were selected from the previous bout if there were insufficient number of seizures in the third recording bout. Following recording, probes were removed and craniotomies sealed with Kwik-Sil or probes were repositioned for a second bout of recording in different probe placement locations. A subset of mice were then recorded again with probe repositioning or 24 hr later with an alternate probe dye-coloring at each location. Mice were used in a maximum of two recordings, whether sequential on the same day or after 24 hours.

Neuropixels probe tract reconstruction

Mice were euthanized with intraperitoneal injection of Fatal+ (Pentobarbital, Vortech Pharmaceutical). We then intracardially perfused 10 ml PBS followed by 10 ml PBS with 4% paraformaldehyde (PFA). Brains were stored overnight in PBS with 4% PFA, washed and stored in PBS until slicing. Serial coronal sections (50 µm) were made on a vibratome (Leica VT1000s). All sections containing probe tracks were mounted in DAPI-Fluoromount-G (Southern Biotech 0100-20). Whole section images were taken spanning all probe tracks on a Zeiss Axio Imager M2 microscope. SHARP-Track (Allen mouse brain common coordinate framework) software was used to reconstruct the 3 dimensional probe locations from the surface of the brain to the tip of each electrode. Regions traversed were assigned to the Allen mouse brain common coordinate framework. Histological boundaries and physiological markers were then used to assign channels to different brain regions. Several functionally similar or difficult to distinguish divisions based on physiological markers and histology are grouped as common structures. The number of animals, distinct probe insertions into a region (Hits). Regions with fewer than 10 neurons were excluded as a hit.

Traversed regions included: (ventrobasal thalamus VB = 4 mice, 8 Hits, 147 neurons); (Reticular thalamic nucleus RT = 4 mice, 7 hits, 389 neurons); (posterior thalamic nucleus PO = 4 mice, 6 hits, 291 neurons), (Mediodorsal thalamus MD = 3 mice, 3 hits, 162 neurons), (ventral lateral thalamus Val = 4 mice, 5 hits, 178 neurons); (lateral dorsal thalamus LD = 4 mice, 6 hits, 339 neurons); (lateral posterior thalamus LP = 3 mice, 4

bioRxiv preprint doi: https://doi.org/10.1101/2025.09.23.678032; this version posted September 16, 2026. The copyright holder for this preprint (which was not certified by peer review) is the author/funder, who has granted bioRxiv a license to display the preprint in perpetuity. It is made available under a CC-BY 4.0 International license.

hits, 90 neurons); (Basal ganglia = 7 mice, 10 hits, 243 neurons); (Som:L1-4 = 7 mice, 10 hits, 121 neurons); (Som:L5 = 7 mice 11 hits, 154 neurons), (Som:L6 = 8 mice, 11 hits, 154 neurons), (CA1 = 5 mice, 7 hits, 68 neurons); (CA3/DG = 4 mice, 7 hits, 60 neurons); (PreMot:L1-4 = 7 mice, 10 hits, 115 neurons); (PreMot:L5 = 7 mice, 9 hits, 115 neurons); (PreMot:L6 = 4 mice 5 hits, 162 neurons); (Orbitofrontal cortex:L1:4 = 5 mice, 7 hits, 352 neurons); (Orbitofrontal cortex:L5 = 5 mice, 7 hits, 278 neurons); (L6 Orbitofrontal cortex:L6 = 3 mice, 4 hits, 277 neurons); (Olfactory areas = 4 mice, 6 hits, 221 neurons).

Ecog spectrogram and filter construction

We computed the spectrogram using Matlab's spectrogram function with an interval of 500 samples, 95% overlap, and an FFT length of 10*500 samples. We constructed ideal passband FIR filters with Matlab's firls function with bands centered at 7 Hz, 14Hz, and 21 Hz with a spread of ± 4 Hz, a transition width of 2 Hz, and order 500 samples resulting in filter amplitudes shown in Fig. 2A. Filters were applied using Matlab's filtfilt function.

Seizure score and Cox proportional hazard model

Seizure score was calculated using custom MATLAB software. We first computed the amplitude for each band and region using the Hilbert transform. A baseline period outside of seizures was used to compute the standard deviation of amplitude for each of the 3 bands. A moving gaussian filter of width 250 ms was applied to remove sharp transients and then seizure score was defined as the average of the 3 band amplitude z-scores. Seizures were then automatically detected as an increase of seizure score over 3 for a period longer than 3 s. Seizure onset time for each electrode was defined as elevation above band averaged z-score of 1 with a subsequent cross of 3 for at least 3 seconds without dropping back below 1 prior to crossing 3. Seizure offset time was defined as the last electrode to fall below a seizure score of 2. Cox proportional hazard model was performed in MATLAB using the coxphfit function on onset times from 30 seizures per mouse from 5 mice.

Measures of Ecog phase and frequency

Phase-coherence was calculated as

$$r = \left| \frac{1}{N} \sum_{n=1}^{N} e^{i\left(\theta_i^n - \theta_j^n\right)} \right| \qquad \text{Equation 3}$$

Where θ_i^n and θ_j^n denote the phase in regions i and j at time point n. For calculation of instantaneous oscillation frequency, the phase angles computed from the Hilbert transform were unwrapped and a 4th order Savitzky-Golay filter was applied with a window size of 1.33, 0.66, and 0.45 s for bands 1, 2, and 3 respectively to cover ~10 oscillation cycles and the rate of change was calculated via the finite difference.[65]

Conversion calculations between SWD-spike time-lags and oscillation phase-lags

To compare the relationship between SWD-spike time-lags and the band specific phase-lags we calculated the mean lag and the 95% range (corresponding to the range of 2 standard deviations). We began by first converting time values of SWD-spike time-lags

bioRxiv preprint doi: https://doi.org/10.1101/2025.09.23.678032; this version posted September 16, 2026. The copyright holder for this preprint (which was not certified by peer review) is the author/funder, who has granted bioRxiv a license to display the preprint in perpetuity. It is made available under a CC-BY 4.0 International license.

between regions to the corresponding predicted phase-lags as follows. The mean MotL-SomL SWD-spike time-lag was -8.2 ± 0.9 ms, indicating SomL spikes typically occur before MotL spikes (example traces Fig. 2K, left, gray circles Fig. 2K, right) with 95% of lags between -19.6 and +3.3 ms (Fig. 2K purple and red circles respectively). We compared this lag to the relative phase-lags of MotL and SomL band 2 (MotLθ_2-SomLθ_2, schematic and example traces, Fig. 2L). Since band 2's frequency is 15Hz (a period of 67 ms), SWD-spike lags of -8.2 ms divided by the period of 67 ms times 2π rad, should correspond to a phase-lag of -0.77 ± 0.1 rad or approximately -π/4 rad (about 1/8th the band 2 period). Similarly, the 95% range of SWD-spike temporal lags from -19.6 ms to +3.3 ms, corresponds to band 2 phase-lags ranging from -1.84 to +0.31 rad, or equivalently from slightly less than -π/2 rad to slightly more than 0 rad. As predicted, the directly measured MotLθ_2-SomLθ_2 phase-lag exhibited a mean of about -π/4 rad (-0.80 ± 0.10 rad) during seizures, with 95% interval between -1.86 ± 0.29 rad and 0.27 ± 0.15 rad (gray, red, and purple trajectories and corresponding circles in Fig. 2M and N). The ~ 0 rad phase-lag in band 2 for MotLθ_2-MotRθ_2 (-0.07 ± 0.05 rad, Fig. 2P schematic and histogram) and SomLθ_2-SomRθ_2 (0.07 ± 0.11 rad, Fig. 2R schematic and histogram). The MotL-MotR SWD-spike time-lag 95% range (from -7.0 to +5.6 ms purple and red circles, Fig. 2P) predicted a MotLθ_2-MotRθ_2 range of -0.66 to 0.53 rad, paralleling the observed phase-lag (-0.42 ± 0.11 and +0.27 ± 0.05 rad, Fig. 2Q purple and red circles). The 95% range for SWD-spike time-lags in SomL-SomR was -9.3 and +10.1 ms (Fig. 2R, purple and red circles), predicting a phase-lag range of -0.87 and 0.95 rad, paralleling the observed SomLθ_2-SomRθ_2 range (-0.74 ± 0.13 to 0.89 ± 0.23 rad, Fig. 2S, purple and red circles).

Spike sorting and Neuropixels data quantification

Spike sorting was performed as outlined previously.[63] Individual units were sorted using Kilosort2 (https://github. com/MouseLand/Kilosort). Unit clusters were manually curated using Phy2 (https:// github.com/cortex-lab/phy). Single units with interspike interval violations >5% percent were excluded from the analysis. Clusters with >20% contamination percentage with neighbor clusters were excluded. Mean firing rate was computed in one second long bins in a 250 ms long sliding window as the number of spikes in a population divided by the number of neurons. Inter-spike intervals distributions were computed for each neuron in a 2 second long window. The distribution of ISIs in the population was then computed for each seizure at times T=-10 s before seizure, T=0, and T=1s after seizure onset. ISIs were converted to frequency as 1/ISI. Probability-normalized histograms were computed using MATLAB's histcounts function. Bin edges spanned from 1 to 1054 Hz in natural log spaced bins of widths of 0.06 (117 bins). The resulting histograms represent the probability distribution of instantaneous firing frequencies of individual neurons in the above time windows. Frequency clustering was computed by finding the peak of the distribution and computing the peak prominence as the difference between the probability peak and 7 frequency bins away (average of each side of the peak). Distributions for individual seizures with less than 20 occupied bins were excluded. To compute firing on phase tori, for each seizure, spike times in the peri-onset window (-0.5 to +0.5 s relative to seizure onset) and phases were extracted from simultaneously recorded local field potentials in the different brain regions (Somatosensory cortex, PreMotor cortex, and screw electrode). We binned the phase tori

bioRxiv preprint doi: https://doi.org/10.1101/2025.09.23.678032; this version posted September 16, 2026. The copyright holder for this preprint (which was not certified by peer review) is the author/funder, who has granted bioRxiv a license to display the preprint in perpetuity. It is made available under a CC-BY 4.0 International license.

in a 60 by 60 grid and computed a 2D histogram of spikes on this grid. To control that some phase combinations occur more rarely along the torus but may highly promote spiking within individual regions we computed the distribution of oscillation phases sampled at all time points within the same analysis window (−0.5 to +0.5 s relative to seizure onset), providing an occupancy reference that controls phase availability independent of spike timing. For each region and phase band, phase distributions were concatenated across seizures and neurons, and normalized probability histograms were constructed.

The expected spike counts per bin were computed by scaling the occupancy map to the total number of observed spikes. Both the observed and expected maps were circularly smoothed (2D Gaussian; σ = 4 bins; wrap-around on both axes). We then calculated a per-bin modulation index (observed number of spikes - expected number of spikes) /expected number of spikes). Statistical significance was assessed by a multinomial shuffle test (1,000 surrogates of spike counts drawn from the occupancy probabilities), yielding two-sided p-values and 95% null confidence intervals; multiple comparisons were controlled with Benjamini–Hochberg FDR at q=0.05. The heat map displays the modulation index.

SINDy method

We sought to identify models in the form:

$$\frac{\mathrm{d}}{\mathrm{d}t}\mathbf{x}(t) = \mathbf{f}\big(\mathbf{x}(t)\big) \qquad \text{Equation 4}$$

The vector $\mathbf{x}(t) \in \mathbb{R}^n$ denotes the system state at time t which consist of the real (x) and imaginary (y) components of the analytic signals $z_{R,B}=x_{R,B}+iy_{R,B}$ as defined in Fig. 2 and where R=1,2,3,4 represents regions MotL, MotR, SomL, and SomR respectively and B=1,2,3 represents frequency bands 1,2, and 3 respectively. Given 4 regions times 3 bands times 2 components (real and imaginary), the dimensionality n of our dynamical system is n=4*3*2=24.The data are sampled at m time points $t_1,t_2,\ldots,t_m$ and represented by an m by n matrix **X** with each row $\mathbf{x}^T$ being an n dimensional system state at a given time:

$$\mathbf{X} = \begin{bmatrix} \mathbf{x}^T(t_1) \\ \mathbf{x}^T(t_2) \\ \vdots \\ \mathbf{x}^T(t_m) \end{bmatrix} = \begin{bmatrix} x_{1,1}(t_1) & y_{1,1}(t_1) & x_{1,2}(t_1) & \ldots & y_{4,3}(t_1) \\ x_{1,1}(t_2) & y_{1,1}(t_2) & x_{1,2}(t_2) & \ldots & y_{4,3}(t_2) \\ \vdots & \vdots & \vdots & \ddots & \vdots \\ x_{1,1}(t_m) & y_{1,1}(t_m) & x_{1,2}(t_m) & \ldots & y_{4,3}(t_m) \end{bmatrix}$$

Derivatives were approximated by the central difference method as $(\mathbf{x}^T(t_{i+1})-\mathbf{x}^T(t_{i-1}))/(2\Delta t)$ and represented by the matrix

$$\dot{\mathbf{X}} = \begin{bmatrix} \dot{\mathbf{x}}^T(t_1) \\ \dot{\mathbf{x}}^T(t_2) \\ \vdots \\ \dot{\mathbf{x}}^T(t_{m-1}) \end{bmatrix} = \begin{bmatrix} \dot{x}_{1,1}(t_1) & \dot{y}_{1,1}(t_1) & \dot{x}_{1,2}(t_1) & \ldots & \dot{y}_{4,3}(t_1) \\ \dot{x}_{1,1}(t_2) & \dot{y}_{1,1}(t_2) & \dot{x}_{1,2}(t_2) & \ldots & \dot{y}_{4,3}(t_2) \\ \vdots & \vdots & \vdots & \ddots & \vdots \\ \dot{x}_{1,1}(t_{m-1}) & \dot{y}_{1,1}(t_{m-1}) & \dot{x}_{1,2}(t_{m-1}) & \ldots & \dot{y}_{4,3}(t_{m-1}) \end{bmatrix}$$

Next we constructed a candidate function library Θ(**X**) consisting of functions of the columns of **X.**

bioRxiv preprint doi: https://doi.org/10.1101/2025.09.23.678032; this version posted September 16, 2026. The copyright holder for this preprint (which was not certified by peer review) is the author/funder, who has granted bioRxiv a license to display the preprint in perpetuity. It is made available under a CC-BY 4.0 International license.

$$\Theta(\mathbf{X}) = \begin{bmatrix} | & | & | & | \\ \mathbf{X}^{(1)} & \mathbf{X}^{(2)} & \mathbf{X}^{(3)} & \mathbf{X}^{(4)} \\ | & | & | & | \end{bmatrix}$$

Where $\mathbf{X}^{(1)}$ denotes linear terms of $x_{R,B}$ and $y_{R,B}$,

$$\mathbf{X}^{(1)} = \begin{bmatrix} \mathbf{x}^T(t_1) \\ \mathbf{x}^T(t_2) \\ \vdots \\ \mathbf{x}^T(t_m) \end{bmatrix} = \begin{bmatrix} x_{1,1}(t_1) & y_{1,1}(t_1) & x_{1,2}(t_1) & \cdots & y_{4,3}(t_1) \\ x_{1,1}(t_2) & y_{1,1}(t_2) & x_{1,2}(t_2) & \cdots & y_{4,3}(t_2) \\ \vdots & \vdots & \vdots & \ddots & \vdots \\ x_{1,1}(t_m) & y_{1,1}(t_m) & x_{1,2}(t_m) & \cdots & y_{4,3}(t_m) \end{bmatrix}$$

$\mathbf{X}^{(2)}$ denotes products of $x_{R,B}$ and $y_{R,B}$ in each possible combination in second order.

$$\mathbf{X}^{(2)} = \begin{bmatrix} \mathbf{x}^T(t_1) \\ \mathbf{x}^T(t_2) \\ \vdots \\ \mathbf{x}^T(t_m) \end{bmatrix} = \begin{bmatrix} x_{1,1}(t_1)^2 & x_{1,1}(t_1)y_{1,1}(t_1) & y_{1,1}(t_1)^2 & \cdots & x_{1,1}(t_1)x_{1,2}(t_1) & \cdots & y_{4,3}(t_1)^2 \\ x_{1,1}(t_2)^2 & x_{1,1}(t_2)y_{1,1}(t_2) & y_{1,1}(t_2)^2 & \cdots & x_{1,1}(t_2)x_{1,2}(t_2) & \cdots & y_{4,3}(t_2)^2 \\ \vdots & \vdots & \vdots & \ddots & \vdots & \ddots & \vdots \\ x_{1,1}(t_m)^2 & x_{1,1}(t_m)y_{1,1}(t_m) & y_{1,1}(t_m)^2 & \cdots & x_{1,1}(t_m)x_{1,2}(t_m) & \cdots & y_{4,3}(t_m)^2 \end{bmatrix}$$

And $\mathbf{X}^{(3)}$ and $\mathbf{X}^{(4)}$ denote similar matrices in 3rd and 4th order respectively.

We then set up a sparse regression problem to determine sparse vectors of coefficients $\Xi = [\xi_1\ \xi_2\ \dots\ \xi_n]$ that determines active terms in the model:

$$\dot{\mathbf{X}} = \Theta(\mathbf{X})\Xi \qquad \text{Equation 5}$$

Each column of Ξ denoted by ξ_k is then a vector of coefficients determining the active terms in $\dot{\mathbf{x}}_k = \mathbf{f}_k(\mathbf{x})$. Governing equations are then represented as

$$\dot{\mathbf{x}}_k = \mathbf{f}_k(\mathbf{x}) = \Theta(\boldsymbol{x}^T)\boldsymbol{\xi}_k$$

Where $\Theta(\mathbf{x}^T)$ is a vector of symbolic functions of $\mathbf{x}$.

Regression and model error

For each library an independent elastic net regression was performed to solve for $\boldsymbol{\xi}_k$ for each real and imaginary component of each region and band separately. Regressions used MATLAB's LASSO function with a λ of 0.04 and an α of 0.9, determined in exploratory steps for parameter ranges which identified stable models. Regression used 10 fold cross-validation. Regressions were performed on the concatenated data of 25 seizures per mouse from 5 mice. Seizure data were from 400 ms before seizure onset to 800 ms after seizure onset (600 samples) for each seizure. 25 simulations (with initial conditions from 5 per mouse) were performed from experimental starting conditions 400 ms before seizure onset. Simulations used MATLAB's ode45 function.

Five measures of model accuracy were used in SINDy library optimization. Total model error was calculated as the average of 5 error measures computed from 25 simulations.

Amplitude correlation error AC_{error} was computed as

$$AC_{error} = \frac{1}{N}\sum_{i=1}^{N}(1 - \rho_i) \qquad \text{Equation 6}$$

bioRxiv preprint doi: https://doi.org/10.1101/2025.09.23.678032; this version posted September 16, 2026. The copyright holder for this preprint (which was not certified by peer review) is the author/funder, who has granted bioRxiv a license to display the preprint in perpetuity. It is made available under a CC-BY 4.0 International license.

where ρ_i is the Pearson correlation coefficient between the experimental and model oscillation amplitude time series for oscillator *i*, and the sum runs over all N = 12 oscillators (4 regions × 3 frequency bands, schematic Fig. S2H, right).

For mean phase-lag between model and experimental distributions we first computed the phase differences $\varphi = (a\theta_i - b\theta_j)$, where i and j are oscillator indices and a and b are band ratios between angles θ_i and θ_j as in Fig. 2 and Fig. S3AB. These were computed for experiment (φ_{Exp}) and model (φ_{Model}), with the distributions for all compared relationships shown in Fig S3AB. Mean phase-lag angle error Lag_{error} was then computed separately for between-band $\text{Lag}_{error(between-band)}$ and between-region $\text{Lag}_{error(between-region)}$ in each case as

$$\text{Lag}_{error} = \frac{1}{M}\sum_{k=1}^{M} \frac{\min\left(\left|\varphi_{Exp,k} - \varphi_{Model,k}\right|, 2\pi - \left|\varphi_{Exp,k} - \varphi_{Model,k}\right|\right)}{\pi} \qquad \text{Equation 7}$$

where k indexes each of the M phase-lag relationships for the between-band (Fig. S3A, schematic Fig. S2H, left) or between-region (Fig. S3B, schematic Fig. S2H, middle) relationships. Each term is normalized to π, since the absolute circular difference spans half the circle, giving an error between 0 and 1.

Phase coherence error Coh_{error} was then similarly computed separately for between-band $Coh_{error\ (between-band)}$ and between region as $Coh_{error\ (between-region)}$

$$Coh_{error} = \frac{1}{M}\sum_{k=1}^{M}\left|r_{Exp,k} - r_{Model,k}\right| \qquad \text{Equation 8}$$

where r is the coherence computed using equation 3 and k indexes each of the M coherence relationships for the between-band (Fig. S3A, schematic Fig. S2H, left) or between-region (Fig. S3B, schematic Fig. S2H, middle) relationships.

Total error was then defined as the average of between these errors as

$$\text{Total error} = \frac{1}{5}\begin{pmatrix} AC_{error} + Lag_{error\ (between-band)} + Coh_{error\ (betwen-band)} \\ +\text{Lag}_{error(between-region)} + Coh_{error(between-region)} \end{pmatrix} \qquad \text{Equation 9}$$

For comparisons to between-mouse variability, the errors computed between mice were computed as above replacing model for traces from each alternate mouse.

Lyapunov spectrum

The Lyapunov spectrum was computed using a tangent-space method with repeated QR re-orthonormalization, following Benettin et al. (1980) and Shimada & Nagashima

bioRxiv preprint doi: https://doi.org/10.1101/2025.09.23.678032; this version posted September 16, 2026. The copyright holder for this preprint (which was not certified by peer review) is the author/funder, who has granted bioRxiv a license to display the preprint in perpetuity. It is made available under a CC-BY 4.0 International license.

(1979).[66, 67] The state equations were integrated jointly with the variational equation for the tangent propagator $\mathbf{\Phi}(t) \in \mathbb{R}^{n\times n}$,

$$\frac{\mathrm{d}}{\mathrm{dt}}\mathbf{\Phi}(t) = \mathbf{J}\big(\mathbf{x}(t)\big)\mathbf{\Phi}(t), \quad \mathbf{\Phi}(t_0) = \mathbf{I}_{\boldsymbol{n}},$$

where $\mathbf{J} = \partial\mathbf{f}/\,\partial\mathbf{x}$ is the Jacobian of the vector field and $\mathbf{I}_{\boldsymbol{n}}$ is the identity matrix. Both $\mathbf{f}$ and $\mathbf{J}$ were derived symbolically (MATLAB Symbolic Math Toolbox).

The augmented $(n + n^2)$-dimensional system was integrated with MATLAB's ode45 with a relative tolerance of 10^{-9} and absolute tolerance of 10^{-12}. To prevent the tangent vectors from collapsing onto the direction of maximal expansion, the integration was performed in chunks of length $\tau = 0.4\ \mathrm{ms}$. At the end of each chunk the tangent matrix was re-orthonormalized via QR decomposition, $\mathbf{\Phi}(t) = \mathbf{QR}$, with the columns of $\mathbf{Q}$ carried forward as the new orthonormal tangent basis. The signs of the diagonal of $\mathbf{R}$ were fixed to be positive before taking logarithms, so that sign flips introduced by the QR factorization do not contaminate the accumulated sums.

The Lyapunov exponents were then obtained as the time averages of the logarithmic expansion rates,

$$\lambda_i = \frac{1}{T}\sum_{k=1}^{K} \ln R_{ii}^{(k)}$$

where $R_{ii}^{(k)}$ is the i-th diagonal element of the upper-triangular factor at the k-th renormalization step and T is the total accumulation time (2000 s). An initial transient of 8 s was discarded before accumulation began, so that the trajectory had relaxed onto the attractor. The initial conditions were drawn independently for each real and imaginary component from a uniform distribution between -150 to 150 µV.

The sum of all exponents (-26.427 ± 1.412 /s, mean standard deviation) was used as a check for dissipative dynamics, as negative sums signify dissipative systems indicative of an attractor. There were two positive exponents (0.255 /s ± 0.010 and 0.073 /s ± 0.004, mean and standard deviation respectively) indicative of chaos, and the third exponent was close to 0 (0.001 /s ± 0.002, mean and standard deviation respectively) indicative of the neutral flow direction. To estimate the attractor's fractal dimension, we computed the Kaplan–Yorke dimension D_{KY}. The exponents were sorted in descending order, and their cumulative sum was calculated (5.480 ± 0.117, mean and standard deviation respectively). D_{KY} was computed as

$$D_{KY} = k + \sum_{i=1}^{k} \frac{\lambda_i}{|\lambda_{k+1}|}$$

Where *k* is the largest integer where the sum of the *k* largest exponents are >= 0.

SINDy library optimization

While $\Theta(\mathbf{X})$ represents a broad set of possible terms and interactions, the vast majority of the coefficients are expected to be 0. In the SINDy method, the candidate function library

bioRxiv preprint doi: https://doi.org/10.1101/2025.09.23.678032; this version posted September 16, 2026. The copyright holder for this preprint (which was not certified by peer review) is the author/funder, who has granted bioRxiv a license to display the preprint in perpetuity. It is made available under a CC-BY 4.0 International license.

must be specified a priori, with a limitation that term selection may become poorly conditioned when the candidate library becomes too large or when the redundancy is too high amongst predictors. In the original formulation of SINDy, it was found that thresholding least squares regression enables pruning of the candidate library in an iterative fashion to promote sparse model identification.[26] This was later found to be related to utilizing L0 norm regularization for term removal.[68] This pruning procedure is based on the value of a thresholding parameter to promote term removal and this was found to result in improved model selection relative to promoting sparsity through using the least absolute shrinkage and selection operator (LASSO) regression.

In our study, we observed that terms varied over several orders of magnitude in their coefficient amplitudes, such that constant thresholding resulted in removal of terms which, though of small magnitude, greatly improved simulated model performance. Levels of thresholding that preserved these terms thus also resulted in other classes of candidate terms receiving no pruning during the application of the iterated thresholding least squares procedure. While normalizing these terms can mitigate this problem, there is little basis for identifying how these thresholds would then be set between different variables. We thus sought to provide a basis for expanding and removing terms from the candidate function library to identify subsets of the library containing terms which are beneficial for model accuracy. To promote parsimony, we sought a nested level of complexity where at each step, increasingly complex subsets of $\Theta(\mathbf{X})$ were included. Library expansions which did not contain accuracy increasing terms (defined as reducing error by >0.01) were removed. Accuracy for library selection was measured at the level of autonomous only function libraries simply as AC_{error}, at the level of between-band within-region functions as $AC_{error} + Lag_{error\ (between-band)} + Coh_{error\ (betwen-band)}$, and at the level of between-band and between-region as $AC_{error} + Lag_{error\ (between-band)} + Coh_{error\ (betwen-band)} + \mathrm{Lag}_{error(between-region)} + Coh_{error(between-region)}$.

Here we briefly describe the ordering of candidate libraries with formal definitions below. We define autonomous terms as including only products consisting of the real and imaginary components of the target signal without including between-band or between-region terms in the library, between-band terms defined as those which include terms of the same region but of other frequency bands, and finally the highest complexity being terms which include between-region terms which are then ordered by complexity, with within-band terms being less complex than between-band terms. Note, these broad classes of libraries were further subdivided during library expansion with increasing complexity of increasing product order containing terms from a single signal followed by product orders consisting of products of two signals (mixed products). The candidate library performance for all library additions is shown in Fig. S4B and the definitions of each addition, their order, number of terms selected/total terms possible to be selected are provided in Table S1. Additionally, models identified with simpler libraries may also contain terms which approximate the roles of more accurate but more complex functions arising in later library expansions, which after library expansion then renders the simpler function obsolete or detrimental to model performance. This suggested that iterative pruning of these functions may also serve to constrain the candidate library size during expansion and to directly compare model performance between competing predictors. Thus, at each stage upon selection of terms from an expanded library, each identified

bioRxiv preprint doi: https://doi.org/10.1101/2025.09.23.678032; this version posted September 16, 2026. The copyright holder for this preprint (which was not certified by peer review) is the author/funder, who has granted bioRxiv a license to display the preprint in perpetuity. It is made available under a CC-BY 4.0 International license.

class of previously selected terms were tested for removal, keeping only terms which degraded model performance by >0.01 with removal.

SINDy library nomenclature

We begin by outlining a notation with which to describe classes of candidate function libraries, which are combined as outlined below to expand the candidate function library in levels of increasing complexity. Each library is defined by the subset of columns (candidate functions) of $\Theta(\mathbf{X})$ that it admits. A general candidate function is a monomial $\prod_p u_{i_p,j_p}$ of degree 1–4, where each factor $u_{i_p,j_p} \in \{x_{i_p,j_p}, y_{i_p,j_p}\}$, i_p indexes region and j_p indexes band. Throughout, (R, B) denotes the region R and band B of the *target* signal, i.e., the pair for which the regression $\dot{x}_{R,B} = \Theta(\mathbf{X})\,\boldsymbol{\xi}$ (and likewise for $\dot{y}_{R,B}$) is being solved, and (i, j), (k, l) denote the region and band indices of the candidate factors.

Band library subset $\Theta(\mathbf{X}_B)$: candidate functions all of whose factors come from band B,

$$\Theta(\mathbf{X}_B) = \left\{\prod_p u_{i_p,j_p} \;\middle|\; j_p = B\ \forall p\right\}$$

Region library subset $\Theta(\mathbf{X}_R)$: candidate functions all of whose factors come from region R,

$$\Theta(\mathbf{X}_R) = \left\{\prod_p u_{i_p,j_p} \;\middle|\; i_p = R\ \forall p\right\}$$

Linear term library subset $\Theta(\mathbf{X}_{\text{Lin}})$: candidate functions of degree 1 (the columns of $\mathbf{X}^{(1)}$),

$$\Theta(\mathbf{X}_{\text{Lin}}) = \{x_{i,j},\ y_{i,j}\}$$

Quadratic term library subset $\Theta(\mathbf{X}_{\text{Quad}})$: candidate functions of degree 2 (the columns of $\mathbf{X}^{(2)}$),

$$\Theta(\mathbf{X}_{\text{Quad}}) = \{x_{i,j}\, x_{k,l},\ \ x_{i,j}\, y_{k,l},\ \ y_{i,j}\, y_{k,l}\}$$

Cubic term library subset $\Theta(\mathbf{X}_{\text{Cub}})$: candidate functions of degree 3 (the columns of $\mathbf{X}^{(3)}$), e.g., $x_{i,j}^2\, x_{k,l}$, $x_{i,j}^2\, y_{k,l}$, etc.

Quartic term library subset $\Theta(\mathbf{X}_{\text{Quart}})$: candidate functions of degree 4 (the columns of $\mathbf{X}^{(4)}$), e.g., $x_{i,j}^3\, x_{k,l}$, $x_{i,j}^2\, x_{k,l}\, y_{k,l}$, etc.

Autonomous function library subset $\Theta(\mathbf{X}_{\text{Aut}})$: candidate functions all of whose factors come from the target signal itself,

$$\Theta(\mathbf{X}_{\text{Aut}}) = \left\{\prod_p u_{i_p,j_p} \;\middle|\; i_p = R \text{ and } j_p = B\ \forall p\right\}$$

Cross-band Intra-region function library subset $\Theta(\mathbf{X}_{\text{CrossBIntraR}})$: candidate functions whose factors come from the target region but products from a single band other than the target band,

bioRxiv preprint doi: https://doi.org/10.1101/2025.09.23.678032; this version posted September 16, 2026. The copyright holder for this preprint (which was not certified by peer review) is the author/funder, who has granted bioRxiv a license to display the preprint in perpetuity. It is made available under a CC-BY 4.0 International license.

$$\Theta(\mathbf{X}_{\text{CrossBIntraR}}) = \left\{\prod_p u_{i_p,j_p} \;\middle|\; i_p = R\ \forall p,\ j_p = j_1\ \forall p,\ j_1 \neq B\right\}$$

Mixed-product cross-band intra-region library subset $\Theta(\mathbf{X}_{\text{MixedProdCrossBIntraR}})$: products within the target region combining at least one factor from the target band with at least one factor from a different band,

$$\Theta(\mathbf{X}_{\text{MixedProdCrossBIntraR}}) = \left\{\prod_p u_{i_p,j_p} \;\middle|\; i_p = R\ \forall p;\ \exists\, p{:}\, j_p = B;\ \exists\, q{:}\, j_q \neq B\right\}$$

Mixed-product cross-region library subset $\Theta(\mathbf{X}_{\text{MixedProdCrossR}})$: products combining factors from at least two different regions,

$$\Theta(\mathbf{X}_{\text{MixedProdCrossR}}) = \left\{\prod_p u_{i_p,j_p} \;\middle|\; \exists\, p, q{:}\, i_p \neq i_q\right\}$$

Cross-regional intra-band library subset $\Theta(\mathbf{X}_{\text{CrossRIntraB}})$: candidate functions whose factors come from regions other than the target region but from the target band,

$$\Theta(\mathbf{X}_{\text{CrossRIntraB}}) = \left\{\prod_p u_{i_p,j_p} \;\middle|\; i_p \neq R \text{ and } j_p = B\ \forall p\right\}$$

Cross-regional cross-band library subset $\Theta(\mathbf{X}_{\text{CrossRCrossB}})$: candidate functions whose factors come from regions other than the target region and from bands other than the target band,

$$\Theta(\mathbf{X}_{\text{CrossRCrossB}}) = \left\{\prod_p u_{i_p,j_p} \;\middle|\; i_p \neq R \text{ and } j_p \neq B\ \forall p\right\}$$

Band-pair restriction subset $\Theta\left(\mathbf{X}_{k/l}\right)$: mixed products whose factors come from the specific pair of bands $\{k, l\}$, $k \neq l$,

$$\Theta\left(\mathbf{X}_{k/l}\right) = \left\{\prod_p u_{i_p,j_p} \;\middle|\; \{j_p\} = \{k, l\}\right\}$$

Region-pair restriction subset $\Theta\left(\mathbf{X}_{G_1/G_2}\right)$: analogously, mixed products whose factors come from the specific pair of region groupings $\{G_1, G_2\}$ (e.g., Mot/Som or Left/Right, where Transcallosal Mot = $\{1,2\}$, Transcallosal Som = $\{3,4\}$, Mot/Som Left = $\{1,3\}$, and Mot/Som Right = $\{2,4\}$.

With these sets we can then express various combinations using the following notation: $A \cup B$ represents the candidate functions in A, in B, or in both; $A \cap B$ represents the candidate functions in both A and B; $A \backslash B$ represents the candidate functions in A but not in B; and $\overline{A}$ indicates the candidate functions of $\Theta(\mathbf{X})$ not in A.

Iterative candidate library expansion and pruning procedure

Next we performed an iterative expansion and pruning procedure where subsets of the above **X** matrices were included in the candidate library as described below where the restricted library is referred to as $\Theta(\mathbf{X}')$. Upon addition of a subset of functions **A** to the current $\Theta(\mathbf{X}')$ matrix, $(\Theta(\mathbf{X}') \cup \Theta(\mathbf{A}))$, a regression was performed and the resultant model was used in simulation and performance was calculated as above. An iterative pruning procedure was then applied to **A** to identify a subset $(\Theta(\mathbf{A}_{\text{Subset}}))$ which maintained or

bioRxiv preprint doi: https://doi.org/10.1101/2025.09.23.678032; this version posted September 16, 2026. The copyright holder for this preprint (which was not certified by peer review) is the author/funder, who has granted bioRxiv a license to display the preprint in perpetuity. It is made available under a CC-BY 4.0 International license.

improved the performance relative to $\Theta(\mathbf{X}') \cup \Theta(\mathbf{A})$. If after pruning, model performance was improved relative to $\Theta(\mathbf{X}')$, $\Theta(\mathbf{X}')$ was redefined as $\Theta(\mathbf{X}') \cup \Theta(\mathbf{A}_{\text{Subset}})$. The pruning procedure for **A** started with product order ($\mathbf{X}_{\text{Lin}}$, $\mathbf{X}_{\text{Quad}}$, $\mathbf{X}_{\text{Cub}}$, and $\mathbf{X}_{\text{Quart}}$), then frequency band of the source of term $\mathbf{X}_{R,B}$ where B=1,2,or 3 and then region source $\mathbf{X}_{R,B}$ R=MotL, MotR, SomL, SomR, and then finally the frequency band and region of the target. Additionally, at the level of each expansion, each addition was tested for amplitude normalized variations of the functions between 0-4th order and the best performing normalization was selected. We performed iterative expansions in the order shown in Table S1.

Library Expansion	(Library Number),Description	Average Number of Selected Terms per oscillator (number in x+y)/Number Possible in Library
$\Theta(\mathbf{X}_{\text{Aut}})$	(1), Autonomous functions	6/28
$\Theta(\mathbf{X}_{\text{CrossBIntraR}})$	(2), Between-band-within-region products excluding the target band in the product.	2/56
$\Theta(\mathbf{X}_{\text{MixedProdCrossB}}) \cap \Theta(\mathbf{X}_{\text{Quad}}) \cup \Theta(\mathbf{X}_{\text{MixedProdCrossB}}) \cap \Theta(\mathbf{X}_{\text{Cub}})$	(3), Quadratic and cubic mixed products between-band-within-region of the source and target region.	6.7/96
$\Theta(\mathbf{X}_{\text{MixedProdCrossBIntraR}}) \cap \Theta(\mathbf{X}_{\text{Quart}}) \cap \Theta(\mathbf{X}_{1/2})$	(4), Quartic mixed products of band 1 and 2 between-band-within-region of the source and target region	0/50
$\Theta(\mathbf{X}_{\text{MixedProdCrossBIntraR}}) \cap \Theta(\mathbf{X}_{\text{Quart}}) \cap \Theta(\mathbf{X}_{1/3})$	(5), Quartic mixed products of band 1 and 3 between-band-within-region of the source and target region	0/50
$\Theta(\mathbf{X}_{\text{MixedProdCrossBIntraR}}) \cap \Theta(\mathbf{X}_{\text{Quart}}) \cap \Theta(\mathbf{X}_{2/3})$	(6), Quartic mixed products of band 2 and 3 between-band-within-region of the source and target region	0/50
$\Theta(\mathbf{X}_{\text{CrossRIntraB}}) \cap \Theta(\mathbf{X}_{\text{Lin}})$	(7), Linear between-region-within-band functions	8/8
$\Theta(\mathbf{X}_{\text{CrossRIntraB}}) \cap \Theta(\mathbf{X}_{\text{Quad}})$	(8), Quadratic between-region-within-band functions	0/12

bioRxiv preprint doi: https://doi.org/10.1101/2025.09.23.678032; this version posted September 16, 2026. The copyright holder for this preprint (which was not certified by peer review) is the author/funder, who has granted bioRxiv a license to display the preprint in perpetuity. It is made available under a CC-BY 4.0 International license.

$\Theta(\mathbf{X}_{CrossRIntraB}) \cap \Theta(\mathbf{X}_{Cub})$	(9), Cubic between-region-within-band functions	0/16
$\Theta(\mathbf{X}_{CrossRIntraB}) \cap \Theta(\mathbf{X}_{Quart})$	(10), Quartic between-region-within-band functions	0/20
$\Theta(\mathbf{X}_{CrossRCrossB}) \cap \Theta(\mathbf{X}_{Lin})$	(11), Linear between-region-between-band functions	0/16
$\Theta(\mathbf{X}_{CrossRCrossB}) \cap \Theta(\mathbf{X}_{Quad})$	(12), Quadratic between-region-between-band functions	0/24
$\Theta(\mathbf{X}_{CrossRCrossB}) \cap \Theta(\mathbf{X}_{Cub})$	(13), Cubic between-region-between-band functions	0/32
$\Theta(\mathbf{X}_{CrossRCrossB}) \cap \Theta(\mathbf{X}_{Quart})$	(14), Quartic between-region-between-band functions	0/40
$\Theta(\mathbf{X}_{MixedProdCrossR}) \cap \Theta(\mathbf{X}_{Mot/Som}) \cap \Theta(\mathbf{X}_{CrossRIntraB})$	(15), Between-region-within-band mixed product Mot/Som functions	1.33/48
$\Theta(\mathbf{X}_{MixedProdCrossR}) \cap \Theta(\mathbf{X}_{L/R}) \cap \Theta(\mathbf{X}_{CrossRIntraB})$	(16), Between-region-within-band mixed product Left/Right functions	0/48
$\Theta(\mathbf{X}_{MixedProdCrossR}) \cap \Theta(\mathbf{X}_{1/2}) \cap \Theta(\mathbf{X}_{CrossRCrossB}) \cap \Theta(\mathbf{X}_{Quad})$	(17), Between-region-between-band mixed product quadratic between band 1 and 2	1.33/32
$\Theta(\mathbf{X}_{MixedProdCrossR}) \cap \Theta(\mathbf{X}_{1/3}) \cap \Theta(\mathbf{X}_{CrossRCrossB}) \cap \Theta(\mathbf{X}_{Quad})$	(18), Between-region-between-band mixed product quadratic between band 1 and 3	0/32
$\Theta(\mathbf{X}_{MixedProdCrossR}) \cap \Theta(\mathbf{X}_{2/3}) \cap \Theta(\mathbf{X}_{CrossRCrossB}) \cap \Theta(\mathbf{X}_{Quad})$	(19), Between-region-between-band mixed product quadratic between band 2 and 3	0/32
$\Theta(\mathbf{X}_{MixedProdCrossR}) \cap \Theta(\mathbf{X}_{1/3}) \cap \Theta(\mathbf{X}_{CrossRCrossB}) \cap \Theta(\mathbf{X}_{Cub})$	(20), Between-region-between-band mixed product cubic between band 1 and 3	2/48
$\Theta(\mathbf{X}_{MixedProdCrossR}) \cap \Theta(\mathbf{X}_{1/2}) \cap \Theta(\mathbf{X}_{CrossRCrossB}) \cap \Theta(\mathbf{X}_{Cub})$	(21), Between-region-between-band mixed product cubic between band 1 and 2	4/48

bioRxiv preprint doi: https://doi.org/10.1101/2025.09.23.678032; this version posted September 16, 2026. The copyright holder for this preprint (which was not certified by peer review) is the author/funder, who has granted bioRxiv a license to display the preprint in perpetuity. It is made available under a CC-BY 4.0 International license.

$\Theta(\mathbf{X}_{\text{MixedProdCrossR}}) \cap \Theta(\mathbf{X}_{2/3}) \cap \Theta(\mathbf{X}_{\text{CrossRCrossB}}) \cap \Theta(\mathbf{X}_{\text{Cub}})$	(22), Between-region-between-band mixed product cubic between 2 and 3	12/48
$\Theta(\mathbf{X}_{\text{MixedProdCrossR}}) \cap \Theta(\mathbf{X}_{1/2}) \cap \Theta(\mathbf{X}_{\text{CrossRCrossB}}) \cap \Theta(\mathbf{X}_{\text{Quart}})$	(23), Between-region-between-band mixed product quartic between 1 and 2	0/100
$\Theta(\mathbf{X}_{\text{MixedProdCrossR}}) \cap \Theta(\mathbf{X}_{1/3}) \cap \Theta(\mathbf{X}_{\text{CrossRCrossB}}) \cap \Theta(\mathbf{X}_{\text{Quart}})$	(24), Between-region-between-band mixed product quartic between band 1 and 3	0/100
$\Theta(\mathbf{X}_{\text{MixedProdCrossR}}) \cap \Theta(\mathbf{X}_{2/3}) \cap \Theta(\mathbf{X}_{\text{CrossRCrossB}}) \cap \Theta(\mathbf{X}_{\text{Quart}})$	(25), Between-region-between-band mixed product quartic between band 2 and 3	0/100

Supp. Table 1. SINDy library expansion order.

SINDy: Autonomous library

Below we show in detail an example of several steps of the above procedure. As an initial library, we first included only autonomous terms, $\Theta(\mathbf{X}') = \Theta(\mathbf{X}_{\text{Aut}})$ and performed the iterative pruning procedure.

$$\Theta(\mathbf{X}_{\text{RemoveLin}}) = \Theta(\mathbf{X}') \backslash \Theta(\mathbf{X}_{\text{Lin}})$$

$$\Theta\left(\mathbf{X}_{\text{RemoveQuad}}\right) = \Theta(\mathbf{X}') \backslash \Theta\left(\mathbf{X}_{\text{Quad}}\right)$$

$$\Theta(\mathbf{X}_{\text{RemoveCub}}) = \Theta(\mathbf{X}') \backslash \Theta(\mathbf{X}_{\text{Cub}})$$

$$\Theta\left(\mathbf{X}_{\text{RemoveQuart}}\right) = \Theta(\mathbf{X}') \backslash \Theta\left(\mathbf{X}_{\text{Quart}}\right)$$

Of these subsets, $\Theta(\mathbf{X}_{\text{RemoveLin}})$ and $\Theta(\mathbf{X}_{\text{RemoveCub}})$ degraded model performance relative to $\Theta(\mathbf{X}')$, while $\Theta(\mathbf{X}_{\text{RemoveQuad}})$ and $\Theta(\mathbf{X}_{\text{RemoveQuart}})$ did not, so $\Theta(\mathbf{X}_{\text{Combined}})$ was defined as

$$\Theta(\mathbf{X}_{\text{Combined}}) = \Theta(\mathbf{X}') \backslash \left(\Theta\left(\mathbf{X}_{\text{Quad}}\right) \cup \Theta\left(\mathbf{X}_{\text{Quart}}\right)\right)$$

We then iteratively removed subsets of the remaining terms ($\Theta(\mathbf{X}_{\text{Lin}})$ and $\Theta(\mathbf{X}_{\text{Cub}})$) by band:

$$\Theta(\mathbf{X}_{\text{RemoveBand1Linear}}) = \Theta(\mathbf{X}_{\text{Combined}}) \backslash \left(\Theta(\mathbf{X}_{\text{Lin}}) \cap \Theta(\mathbf{X}_1)\right)$$

$$\Theta(\mathbf{X}_{\text{RemoveBand2Linear}}) = \Theta(\mathbf{X}_{\text{Combined}}) \backslash \left(\Theta(\mathbf{X}_{\text{Lin}}) \cap \Theta(\mathbf{X}_2)\right)$$

$$\vdots$$

$$\Theta(\mathbf{X}_{\text{RemoveBand3Cubic}}) = \Theta(\mathbf{X}_{\text{Combined}}) \backslash \left(\Theta(\mathbf{X}_{\text{Cub}}) \cap \Theta(\mathbf{X}_3)\right)$$

This resulted in the preservation of all sub-band terms for these product orders, leading to a redefinition of

bioRxiv preprint doi: https://doi.org/10.1101/2025.09.23.678032; this version posted September 16, 2026. The copyright holder for this preprint (which was not certified by peer review) is the author/funder, who has granted bioRxiv a license to display the preprint in perpetuity. It is made available under a CC-BY 4.0 International license.

$$\Theta(\mathbf{X}') \rightarrow \Theta(\mathbf{X}') \backslash \left(\left(\Theta(\mathbf{X}_{\mathrm{Aut}}) \cap \Theta\left(\mathbf{X}_{\mathrm{Quad}}\right) \right) \cup \left(\Theta(\mathbf{X}_{\mathrm{Aut}}) \cap \Theta\left(\mathbf{X}_{\mathrm{Quart}}\right) \right) \right)$$

Each x and y each had 14 autonomous terms possible for inclusion in the model and 6 were selected for each x and y.

We noted at this stage that there are regularities in the magnitudes of coefficients which suggest they may be combined to form common coefficients. Below we show this procedure for the selected linear and cubic terms in the model at this step. The selected terms followed the following pattern:

$$\frac{\mathrm{d}x_{R,B}}{\mathrm{dt}} = \alpha x_{R,B} - \beta y_{R,B}$$

$$\frac{\mathrm{d}y_{R,B}}{\mathrm{dt}} = \gamma x_{R,B} + \delta y_{R,B}$$

Where α, β, γ, and δ are the corresponding coefficients in ξ_k. Coefficients α and δ were found to be near identical and β and γ were found to be near identical. We rename these common coefficients μ (α and δ) and ω (β and γ) due to their dynamical roles in intrinsic growth (μ) and intrinsic oscillation frequency (ω):

$$\frac{\mathrm{d}x_{R,B}}{\mathrm{dt}} = \mu x_{R,B} - \omega y_{R,B}$$

$$\frac{\mathrm{d}y_{R,B}}{\mathrm{dt}} = \omega x_{R,B} + \mu y_{R,B}$$

This pattern of coefficients allows us to combine terms into an equation in the complex variable z:

$$\frac{\mathrm{d}z_{R,B}}{\mathrm{dt}} = \mu x_{R,B} - \omega y_{R,B} + \mathrm{i}(\omega x_{R,B} + \mu y_{R,B})$$

$$\frac{\mathrm{d}z_{R,B}}{\mathrm{dt}} = (\mu + \mathrm{i}\omega) z_{R,B}$$

We then changed coordinates from *z* to amplitude A and phase θ:

$$\mathrm{e}^{\mathrm{i}\theta_{R,B}} \left(\frac{\mathrm{d}A_{R,B}}{\mathrm{dt}} + \mathrm{i}A_{R,B} \frac{\mathrm{d}\theta_{R,B}}{\mathrm{dt}} \right) = (\mu + \mathrm{i}\omega) A_{R,B} \mathrm{e}^{\mathrm{i}\theta_{R,B}}$$

$$\left(\frac{\mathrm{d}A_{R,B}}{\mathrm{dt}} + \mathrm{i}A_{R,B} \frac{\mathrm{d}\theta_{R,B}}{\mathrm{dt}} \right) = (\mu + \mathrm{i}\omega) A_{R,B}$$

$$\frac{\mathrm{d}A_{R,B}}{\mathrm{dt}} = \mu A_{R,B}$$

$$\frac{\mathrm{d}\theta_{R,B}}{\mathrm{dt}} = \omega$$

Region	$dx_{(R,B)}/dt=\omega y_{(R,B)}$	$dy_{(R,B)}/dt = \omega x_{(R,B)}$	**Simplified Function** and Remainder	Intrinsic Frequency

bioRxiv preprint doi: https://doi.org/10.1101/2025.09.23.678032; this version posted September 16, 2026. The copyright holder for this preprint (which was not certified by peer review) is the author/funder, who has granted bioRxiv a license to display the preprint in perpetuity. It is made available under a CC-BY 4.0 International license.

	dx(R,B)	$\gamma * y_{R,B}$	$\beta * x_{R,B}$	$dz_{(R,B)}/dt=$	
MotL Band 1	$(_{1,1})$	-18.33	18.35	**(18.33 i) $z_{1,1}$** + (0.013i)Re($z_{1,1}$)	6.3 Hz
MotR Band 1	$(_{2,1})$	-19.38	19.38	**(19.38 i) $z_{2,1}$** + (0.01i)Re($z_{2,1}$)	6.7 Hz
SomL Band1	$(_{3,1})$	-20.56	20.52	**(20.56 i) $z_{3,1}$** - (0.04i)Re($z_{3,1}$)	7.1 Hz
SomR Band 1	$(_{4,1})$	-21.75	21.75	**(21.8 i) $z_{4,1}$** + (0.00i)Re($z_{4,1}$)	7.54 Hz
MotL Band 2	$(_{1,2})$	-35.49	35.89	**(35.49 i) $z_{1,2}$** + (0.39i)Re($z_{1,2}$)	12.3 Hz
MotR Band 2	$(_{2,2})$	-37.44	38.08	**(37.74 i) $z_{2,2}$** +(0.63i)Re($z_{2,2}$)	13.1 Hz
SomL Band 2	$(_{3,2})$	-40.37	40.36	**(40.6 i) $z_{3,2}$** -(0.00i)Re($z_{3,2}$)	14.1 Hz
SomR Band 2	$(_{4,2})$	-40.83	40.85	**(40.83 i) $z_{4,2}$** + (0.02i)Re($z_{4,2}$)	14.1 Hz
MotL Band 3	$(_{1,3})$	-51.02	50.99	**(51.02 i) $z_{1,3}$** - (0.03i)Re($z_{1,3}$)	17.7 Hz
MotR Band 3	$(_{2,3})$	-54.12	54.10	**(54.12 i) $z_{2,3}$** - (0.02i)Re($z_{2,3}$)	18.7 Hz
SomL Band 3	$(_{3,3})$	-56.29	56.35	**(56.29 i) $z_{3,3}$** + (0.07i)Re($z_{3,3}$)	19.5 Hz
SomR Band 3	$(_{4,3})$	-56.45	56.47	**(56.45 i) $z_{4,3}$** + (0.02i)Re($z_{4,3}$)	19.5 Hz

Supp. Table 2. Linear autonomous coefficients in the model. Algebraic simplification constructs intrinsic frequency (ωi)z. Bold indicates the simplified function in terms of z.

Notably, while the μ terms are selected at the introduction of the library, they were later pruned after the introduction of cross regional coupling library 7 (between-region coupling within band). We show the final values of ω in the final model and the remainder after simplification. The following coefficients are divided by a normalizing scale factor of 230 to put them on a regular scale across terms. Truncation of the remainders did not affect total error (0.19 vs. 0.19).

We also observed for cubic autonomous terms several common coefficient amplitude terms. Here we combine directly to α and β, named for their roles in cubic nonlinear oscillator behavior.

Cubic terms:

$$\frac{\mathrm{d}x_{R,B}}{\mathrm{dt}} = -\alpha x_{R,B}x_{R,B}x_{R,B} - \alpha x_{R,B}y_{R,B}y_{R,B} + \beta x_{R,B}x_{R,B}y_{R,B} + \beta y_{R,B}y_{R,B}y_{R,B}$$

$$\frac{\mathrm{d}y_{R,B}}{\mathrm{dt}} = -\beta x_{R,B}x_{R,B}x_{R,B} - \beta x_{R,B}y_{R,B}y_{R,B} - \alpha x_{R,B}x_{R,B}y_{R,B} - \alpha y_{R,B}y_{R,B}y_{R,B}$$

$$\frac{\mathrm{d}z_{R,B}}{\mathrm{dt}} = -\alpha x_{R,B}x_{R,B}x_{R,B} - \alpha x_{R,B}y_{R,B}y_{R,B} + \beta x_{R,B}x_{R,B}y_{R,B} + \beta y_{R,B}y_{R,B}y_{R,B} + \mathrm{i}(-\beta x_{R,B}x_{R,B}x_{R,B} - \beta x_{R,B}y_{R,B}y_{R,B} - \alpha x_{R,B}x_{R,B}y_{R,B} - \alpha y_{R,B}y_{R,B}y_{R,B})$$

$$\frac{\mathrm{d}z_{R,B}}{\mathrm{dt}} = (-\alpha - \mathrm{i}\beta)z_{R,B}\left|z_{R,B}\right|^2$$

bioRxiv preprint doi: https://doi.org/10.1101/2025.09.23.678032; this version posted September 16, 2026. The copyright holder for this preprint (which was not certified by peer review) is the author/funder, who has granted bioRxiv a license to display the preprint in perpetuity. It is made available under a CC-BY 4.0 International license.

$$e^{i\theta_{R,B}}\left(\frac{dA_{R,B}}{dt} + iA_{R,B}\frac{d\theta_{R,B}}{dt}\right) = (-\alpha - i\beta)A_{R,B}^3 e^{i\theta_{R,B}}$$

$$\left(\frac{dA_{R,B}}{dt} + iA_{R,B}\frac{d\theta_{R,B}}{dt}\right) = (-\alpha - i\beta)A_{R,B}^3$$

$$\frac{dA_{R,B}}{dt} = -\,\alpha A_{R,B}^3$$

$$\frac{d\theta_{R,B}}{dt} = -\beta A_{R,B}^2$$

Notably at this stage, the combination of the linear and cubic functions reveals a common structure where the normal form for the Hopf bifurcation is identified with bifurcation parameter μ, limit cycle term α, natural oscillation frequency term ω, and an amplitude dependent frequency term β. Of the 28 possible coefficients for each oscillator (14 for each x and y) there were 12 terms selected at this stage (6 for each x and y), which when common amplitude coefficients are combined reduce to a 4-parameter model for each oscillator:

$$\frac{dA_{R,B}}{dt} = \mu A_{R,B} - \alpha A_{R,B}^3$$

$$\frac{d\theta_{R,B}}{dt} = \omega - \beta A_{R,B}^2$$

While a 4 term model was identified at this stage, μ and β were later culled upon addition of linear cross regional coupling and mixed products cross frequency libraries respectively reducing the number to 6 terms selected per oscillator (3 for each x and y) which reduce to the two autonomous parameters shown in Fig. 4A. Additionally, while the coefficients for α are conserved robustly up to the level of within-band cross-regional coupling, they exhibit a decrease in amplitude in the final model and exhibit higher remainders after factoring out the common terms for α. The components going into the final model are as follows. The following coefficients are divided by a normalizing scale factor of 7543457397 to put them on a regular scale across terms. Truncation of the remainders did not affect total error (0.19 vs. 0.19).

Region	$dx_{(R,B)}/dt = \alpha\, x_{R,B}\, x_{R,B}\, x_{R,B} + \alpha\, y_{R,B}\, y_{R,B}\, x_{R,B}$			$dy_{(R,B)}/dt = \alpha\, y_{R,B}\, x_{R,B}\, x_{R,B} + \alpha\, y_{R,B}\, y_{R,B} y_{R,B}$	
	(R,B)	$\alpha\, x_{R,B}\, x_{R,B}\, x_{R,B}$	$\alpha\, y_{R,B}\, y_{R,B}\, x_{R,B}$	$\alpha\, y_{R,B}\, x_{R,B}\, x_{R,B}$	$\alpha\, y_{R,B}\, y_{R,B} y_{R,B}$
MotL Band 1	$(_{1,1})$	-2.56	-2.53	-2.73	-2.64
MotR Band 1	$(_{2,1})$	-0.30	-0.13	-0.13	-0.23
SomL Band1	$(_{3,1})$	0.34	-1.28	1.03	-0.25
SomR Band 1	$(_{4,1})$	-0.30	-2.78	-0.28	-1.05
MotL Band 2	$(_{1,2})$	-12.11	-11.89	-11.24	-11.52

bioRxiv preprint doi: https://doi.org/10.1101/2025.09.23.678032; this version posted September 16, 2026. The copyright holder for this preprint (which was not certified by peer review) is the author/funder, who has granted bioRxiv a license to display the preprint in perpetuity. It is made available under a CC-BY 4.0 International license.

MotR Band 2	($_{2,2}$)	-2.63	-2.36	-2.44	-2.63
SomL Band 2	($_{3,2}$)	-1.75	-1.57	-1.73	-1.93
SomR Band 2	($_{4,2}$)	-2.44	-2.39	-2.21	-2.49
MotL Band 3	($_{1,3}$)	-9.69	-8.63	-10.17	-10.14
MotR Band 3	($_{2,3}$)	-4.80	-3.75	-5.12	-5.00
SomL Band 3	($_{3,3}$)	-9.36	-9.05	-8.49	-9.04
SomR Band 3	($_{4,3}$)	-5.59	-5.25	-4.69	-5.16
Region		**Simplified Function** and remainder dz($_{R,B}$)/dt=			
MotL Band 1	($_{1,1}$)	**- 2.61 $z_{R,B}$ |$z_{R,B}$|^2**+0.06$z_{R,B}$ ^3 + 0.01 $z_{R,B}$ ($z_{R,B}$ *)^2 - 0.02($z_{R,B}$ *)^3			
MotR Band 1	($_{2,1}$)	**- 0.23 $z_{R,B}$ |$z_{R,B}$ |^2** -0.03 $z_{R,B}$ ^3 - 0.03 $z_{R,B}$ ($z_{R,B}$ *)^2 - 0.01 ($z_{R,B}$ *)^3			
SomL Band1	($_{3,1}$)	**0.00 $z_{R,B}$ |$z_{R,B}$ |^2** -0.07$z_{R,B}$ ^3 + 0.04 $z_{R,B}$ ($z_{R,B}$ *)^2 +0.36 ($z_{R,B}$ *)^3			
SomR Band 1	($_{4,1}$)	**-0.89$z_{R,B}$ |$z_{R,B}$ |^2** -0.41 $z_{R,B}$^3 - 0.03$z_{R,B}$ ($z_{R,B}$ *)^2 +0.21 ($z_{R,B}$ *)^3			
MotL Band 2	($_{1,2}$)	**-11.75 $z_{R,B}$ |$z_{R,B}$ |^2**-0.01$z_{R,B}$ ^3 - 0.3 $z_{R,B}$ ($z_{R,B}$ *)^2 -0.06 ($z_{R,B}$ *)^3			
MotR Band 2	($_{2,2}$)	**- 2.57$z_{R,B}$ |$z_{R,B}$ |^2**-0.01 $z_{R,B}$ ^3 +0.12 $z_{R,B}$ ($z_{R,B}$ *)^2 - 0.06 ($z_{R,B}$ *)^3			
SomL Band 2	($_{3,2}$)	**- 1.79 $z_{R,B}$ |$z_{R,B}$ |^2** +0.00 $z_{R,B}$ ^3 + 0.09 $z_{R,B}$ ($z_{R,B}$ *)^2 - 0.05 ($z_{R,B}$ *)^3			
SomR Band 2	($_{4,2}$)	**- 2.42 $z_{R,B}$ abs($z_{R,B}$)^2**+0.027 $z_{R,B}$ ^3 + 0.00 $z_{R,B}$ ($z_{R,B}$ *)^2 - 0.04 ($z_{R,B}$ *)^3			
MotL Band 3	($_{1,3}$)	**- 9.79 $z_{R,B}$ |$z_{R,B}$ |^2**-0.14 $z_{R,B}$ ^3 + 0.36 $z_{R,B}$ ($z_{R,B}$ *)^2 - 0.13 ($z_{R,B}$ *)^3			
MotR Band 3	($_{2,3}$)	**- 4.78 $z_{R,B}$ |$z_{R,B}$ |^2**-0.15 $z_{R,B}$ ^3 + 0.25 $z_{R,B}$ ($z_{R,B}$ *)^2 - 0.11 ($z_{R,B}$ *)^3			
SomL Band 3	($_{3,3}$)	**- 9.09 $z_{R,B}$ |$z_{R,B}$ |^2**+0.03 $z_{R,B}$ ^3 - 0.19 $z_{R,B}$ ($z_{R,B}$ *)^2 - 0.11 ($z_{R,B}$ *)^3			
SomR Band 3	($_{4,3}$)	**- 5.28 $z_{R,B}$ |$z_{R,B}$ |^2**+0.02$z_{R,B}$ ^3 - 0.02 $z_{R,B}$ ($z_{R,B}$ *)^2 - 0.10 ($z_{R,B}$ *)^3			

Supp. Table 3. Cubic autonomous coefficients in the model. Algebraic simplification constructs cubic limit cycle term α $z_{R,B}$ |$z_{R,B}$ |^2. Bold indicates the simplified function in z.

SINDy: Between-band coupling libraries

The next expansion was cross frequency coupling terms to the candidate function library ($\Theta(\mathbf{X}') \cup \Theta(\mathbf{X}_{CrossBIntraR})$). This expansion includes 28 functions for each x and y.

We then sought sub-regions within $\Theta(\mathbf{X}_{CrossBIntraR})$ which contributed to the improved model performance of $\Theta(\mathbf{X}') \cup \Theta(\mathbf{X}_{CrossBIntraR})$ relative to $\Theta(\mathbf{X}')$.

We defined subregions first by the product order within the expansion for terms which are linear, quadratic, cubic, or quartic and cross frequency:

$$\Theta(\mathbf{X}_{LinCrossB}) = \Theta(\mathbf{X}_{CrossIntraR}) \cap \Theta(\mathbf{X}_{Lin})$$

bioRxiv preprint doi: https://doi.org/10.1101/2025.09.23.678032; this version posted September 16, 2026. The copyright holder for this preprint (which was not certified by peer review) is the author/funder, who has granted bioRxiv a license to display the preprint in perpetuity. It is made available under a CC-BY 4.0 International license.

$$\Theta(\mathbf{X}_{QuadCrossB}) = \Theta(\mathbf{X}_{CrossIntraR}) \cap \Theta(\mathbf{X}_{Quad})$$

…

We then test the removal of each of the above subsets from $\Theta(\mathbf{X}') \cup \Theta(\mathbf{X}_{CrossB})$:

$$\Theta(\mathbf{X}_{RemoveLin}) = \Theta(\mathbf{X}') \cup \Theta(\mathbf{X}_{CrossBIntraR}) \backslash \Theta(\mathbf{X}_{LinCrossB})$$

$$\Theta(\mathbf{X}_{RemoveQuad}) = \Theta(\mathbf{X}') \cup \Theta(\mathbf{X}_{CrossBIntraR}) \backslash \Theta(\mathbf{X}_{QuadCrossB})$$

$$\Theta(\mathbf{X}_{RemoveCub}) = \Theta(\mathbf{X}') \cup \Theta(\mathbf{X}_{CrossBIntraR}) \backslash \Theta(\mathbf{X}_{CubCrossB})$$

$$\Theta(\mathbf{X}_{RemoveQuart}) = \Theta(\mathbf{X}') \cup \Theta(\mathbf{X}_{CrossBIntraR}) \backslash \Theta(\mathbf{X}_{QuartCrossB})$$

Of the subsets, $\Theta(\mathbf{X}_{RemoveQuad})$ and $\Theta(\mathbf{X}_{RemoveCub})$ degraded model performance relative to $\Theta(\mathbf{X}')$ while $\Theta(\mathbf{X}_{RemoveLin})$ *and* $\Theta(\mathbf{X}_{RemoveQuart})$ did not so $\Theta(\mathbf{X}_{CombinedOrder})$ was defined:

$$\Theta(\mathbf{X}_{CombinedOrder}) = \Theta(\mathbf{X}') \cup \Theta(\mathbf{X}_{QuadCrossB}) \cup \Theta(\mathbf{X}_{CubCrossB})$$

We then removed subsets of $\Theta(\mathbf{X}_{QuadCrossB})$ *and* $\Theta(\mathbf{X}_{CubCrossB})$ by band

$$\Theta(\mathbf{X}_{RemoveBand1}) = \Theta(\mathbf{X}_{CombinedOrder}) \backslash (\Theta(\mathbf{X}_{QuadCrossB}) \cap \Theta(\mathbf{X}_1))$$

$$\Theta(\mathbf{X}_{RemoveBand2}) = \Theta(\mathbf{X}_{CombinedOrder}) \backslash (\Theta(\mathbf{X}_{QuadCrossB}) \cap \Theta(\mathbf{X}_2))$$

…

Which resulted in the sub regions of $\mathbf{X}_{CrossBIntraR}$ included in the candidate function being:

$$\Theta(\mathbf{X}') \rightarrow \Theta(\mathbf{X}') \cup (\Theta(\mathbf{X}_{CrossBIntraR}) \cap \Theta(\mathbf{X}_{Quad}) \cap \Theta(\mathbf{X}_2)) \cup (\Theta(\mathbf{X}_{CrossBIntraR}) \cap \Theta(\mathbf{X}_{Cub}) \cap \Theta(\mathbf{X}_3))$$

Examination of the functions built for cross frequency interactions at this step identified a common structure which is limited to quadratic cross frequency functions in band 2 terms and cubic cross frequency functions in band 3 terms while all linear and quartic functions were removed. The selected functions exhibited a high degree of regularity as observed above with the Autonomous library, resulting in common coefficients. For band 1 coupling to band 2, we identify equations of the following form:

$$\frac{\mathrm{d}x_{R,2}}{dt} = -ax_{R,1}x_{R,1} + 2bx_{R,1}y_{R,1} + ay_{R,1}y_{R,1}$$

$$\frac{\mathrm{d}y_{R,2}}{\mathrm{dt}} = -bx_{R,1}x_{R,1} - 2ax_{R,1}y_{R,1} + by_{R,1}y_{R,1}$$

Where a and b are coefficients in ξ_k. We then combined common terms as above and converted to polar coordinates, yielding cross frequency coupling functions which relate the product order (quadratic) to the frequency ratio between the source and target.

$$\frac{\mathrm{d}z_{R,2}}{\mathrm{dt}} = -ax_{R,1}x_{R,1} + 2bx_{R,1}y_{R,1} + ay_{R,1}y_{R,1} + \mathrm{i}(-bx_{R,1}x_{R,1} - 2ax_{R,1}y_{R,1} + by_{R,1}y_{R,1})$$

$$\frac{\mathrm{d}z_{R,2}}{\mathrm{dt}} = (-a - \mathrm{i}b)z_{R,1}^2$$

$$\mathrm{e}^{\mathrm{i}\theta_{R,2}}\left(\frac{\mathrm{d}A_{R,2}}{\mathrm{dt}} + \mathrm{i}A_{R,2}\frac{\mathrm{d}\theta_{R,2}}{\mathrm{dt}}\right) = (-a - \mathrm{i}b)A_{R,1}^2\mathrm{e}^{\mathrm{i}2\theta_{R,1}}$$

bioRxiv preprint doi: https://doi.org/10.1101/2025.09.23.678032; this version posted September 16, 2026. The copyright holder for this preprint (which was not certified by peer review) is the author/funder, who has granted bioRxiv a license to display the preprint in perpetuity. It is made available under a CC-BY 4.0 International license.

$$\left(\frac{\mathrm{d}A_{R,2}}{\mathrm{dt}} + \mathrm{i}A_{R,2}\frac{\mathrm{d}\theta_{R,2}}{\mathrm{dt}}\right) = (-a - \mathrm{i}b)A_{R,1}^2\mathrm{e}^{\mathrm{i}(2\theta_{R,1}-\theta_{R,2})}$$

$$\left(\frac{\mathrm{d}A_{R,2}}{\mathrm{dt}} + \mathrm{i}A_{R,2}\frac{\mathrm{d}\theta_{R,2}}{\mathrm{dt}}\right) = (-a - \mathrm{i}b)A_{R,1}^2((\cos\left(2\theta_{R,1} - \theta_{R,2}\right) + \mathrm{i}\sin\left(2\theta_{R,1} - \theta_{R,2}\right))$$

$$\frac{\mathrm{d}A_{R,2}}{\mathrm{dt}} = A_{R,1}^2\left(-a\cos\left(2\theta_{R,1} - \theta_{R,2}\right) + b\sin\left(2\theta_{R,1} - \theta_{R,2}\right)\right)$$

$$\frac{\mathrm{d}\theta_{R,2}}{\mathrm{dt}} = \frac{A_{R,1}^2}{A_{R,2}}\left(-b\cos\left(2\theta_{R,1} - \theta_{R,2}\right) - a\sin\left(2\theta_{R,1} - \theta_{R,2}\right)\right)$$

$$\frac{\mathrm{d}A_{R,2}}{\mathrm{dt}} = CA_{R,1}^2\cos(2\theta_{R,1} - \theta_{R,2} - \varphi_1)$$

$$\frac{\mathrm{d}\theta_{R,2}}{\mathrm{dt}} = C\frac{A_{R,1}^2}{A_{R,2}}\cos(2\theta_{R,1} - \theta_{R,2} - \varphi_2)$$

Where $C = \sqrt{a^2 + b^2}$, $\varphi_1 = \arctan\left(\frac{b}{-a}\right)$, $\varphi_2 = \arctan\left(\frac{-a}{-b}\right)$

Here, coupling strength grows with $A^2_{R,1}$ for amplitude and $A^2_{R,1}/A_{R,2}$ for phase coupling. This results in negligible modulation at 200 µV but grows to levels comparable to between-regional coupling at 1000 µV (Fig. S5I and J). Thus amplitude increases band 1-to-2 coupling strength, explaining the absence of these phase relationships outside seizures in experimental data (Fig. 2F, Fig. S2I).

In the final model, the Mot region deviated from the above relatively simple pattern though plotting the function on the torus gave an overall very similar form as the Som regions but with an additional distortion along diagonal trajectories (Fig. S5IJ). The following coefficients are divided by a normalizing scale factor of 19763881.45 to put them on a regular scale across terms.

		$dx_{(R,2)}/dt = a x_{R,1} x_{R,1} + b x_{R,1} y_{R,1} + a y_{R,1} y_{R,1}$			$dy_{(R,2)}/dt = b x_{R,1} x_{R,1} + a x_{R,1} y_{R,1} + b y_{R,1} y_{R,1}$		
Connection	(R,B)	$a\, x_{R,1} x_{R,1}$	$b\, x_{R,1} y_{R,1}$	$a\, y_{R,1} y_{R,1}$	$b\, x_{R,1} x_{R,1}$	$a\, x_{R,1} y$	$b\, y_{R,1} y_{R,1}$
MotL Band1 to MotL Band2	(1,2)	-43.90	171.01	0	-40.50	0	60.32
MotR Band1 to MotR Band2	(2,2)	-27.84	232.22	76.32	-30.16	-85.01	92.59
SomL Band1 to SomL Band2	(3,2)	-33.86	77.63	33.65	-38.30	-69.36	38.29
SomR Band1 to SomR Band2	(4,2)	-38.06	106.67	37.58	-52.36	-76.41	52.31
Connection	(R,B)	**Simplified Function** and remainder $dz_{(R,B)}/dt=$					

bioRxiv preprint doi: https://doi.org/10.1101/2025.09.23.678032; this version posted September 16, 2026. The copyright holder for this preprint (which was not certified by peer review) is the author/funder, who has granted bioRxiv a license to display the preprint in perpetuity. It is made available under a CC-BY 4.0 International license.

MotLBand1 to MotL Band2	(1,2)	(-43.9 – 40.5 i) Re($z_{R,1}$)^2+ (171) Re($z_{R,1}$)*Im($z_{R,1}$)+60Im($z_{R,1}$*)^2
MotRBand1 to MotR Band2	(2,2)	**(-71.53 -119.95 i) $z_{R,1}$^2** +(-4.79 + 27.36 i) ($z_{R,1}$*)^2 + - (48.48+ 62.43 i) $z_{R,1}$Re($z_{R,1}$)
SomLBand1 to SomL Band2	(3,2)	**(-34.12 -38.56 i) $z_{R,1}$^2**+(0.47 - 0.26 i) ($z_{R,1}$*)^2 - (0.21 - 0.0 i) $z_{R,1}$ Re($z_{R,1}$)
SomRBand1 to SomR Band2	(4,2)	**(-38.52- 52.81 i) $z_{R,1}$^2**+(-0.06- 0.50 i) ($z_{R,1}$*)^2 + -(0.52 + 0.046 i) $z_{R,1}$Re($z_{R,1}$)

Supp. Table 4. Band 1 to 2 coupling functions in the model. Bold indicates the simplified function in z.

We next expanded to include mixed products consisting of mixed quadratic functions and mixed cubic functions. Several functions were selected at this stage, many of which were pruned in the later models when cross regional functions were included in the library. The functions that remain in the final model at this stage are those shown graphically in Fig. 4B, middle, see accompanying MATLAB code for model expansion and sparsification steps. These functions contributed relatively little to overall model performance (Fig. S4F). See accompanying model spreadsheet for the exact coefficients.

SINDy: Between-region coupling libraries

We next added linear cross regional coupling within band functions to the candidate function library. We observe that two different normalized variants are selected. Only the MotL/R regions selected the un-normalized amplitude variants.

We identify functions of the following form:

$$\frac{\mathrm{d}x_{j,B}}{\mathrm{dt}} = (\pm a x_{k,B} \pm b y_{k,B})/A_{k,B}$$

$$\frac{\mathrm{d}y_{j,B}}{\mathrm{dt}} = (\pm b x_{k,B} \pm a y_{k,B})/A_{k,B}$$

$$\frac{\mathrm{d}z_{j,B}}{\mathrm{dt}} = (a + \mathrm{i}b) z_{k,B}/A_{k,B}$$

$$\mathrm{e}^{\mathrm{i}\theta_{j,B}}\left(\frac{\mathrm{d}A_{j,B}}{\mathrm{dt}} + \mathrm{i}A_{j,B}\frac{\mathrm{d}\theta_{j,B}}{\mathrm{dt}}\right) = ((a + \mathrm{i}b)A_{k,B}\mathrm{e}^{\mathrm{i}\theta_{k,B}})/A_{k,B}$$

$$\left(\frac{\mathrm{d}A_{j,B}}{\mathrm{dt}} + \mathrm{i}A_{j,B}\frac{\mathrm{d}\theta_{j,B}}{\mathrm{dt}}\right) = ((a + \mathrm{i}b)A_{k,B}\mathrm{e}^{\mathrm{i}(\theta_{k,B} - \theta_{j,B})})/A_{k,B}$$

$$\left(\frac{\mathrm{d}A_{j,B}}{\mathrm{dt}} + \mathrm{i}A_{j,B}\frac{\mathrm{d}\theta_{j,B}}{\mathrm{dt}}\right) = (a + \mathrm{i}b)(\cos\left(\theta_{k,B} - \theta_{j,B}\right) + \mathrm{i}\sin\left(\theta_{k,B} - \theta_{j,B}\right))$$

bioRxiv preprint doi: https://doi.org/10.1101/2025.09.23.678032; this version posted September 16, 2026. The copyright holder for this preprint (which was not certified by peer review) is the author/funder, who has granted bioRxiv a license to display the preprint in perpetuity. It is made available under a CC-BY 4.0 International license.

$$\frac{\mathrm{d}A_{j,B}}{\mathrm{dt}} = \left(a\cos\left(\theta_{k,B} - \theta_{j,B}\right) - b\sin\left(\theta_{k,B} - \theta_{j,B}\right)\right)$$

$$\frac{\mathrm{d}\theta_{j,B}}{\mathrm{dt}} = \frac{1}{A_{j,B}}\left(b\cos\left(\theta_{k,B} - \theta_{j,B}\right) + a\sin\left(\theta_{k,B} - \theta_{j,B}\right)\right)$$

$$\frac{\mathrm{d}A_{j,B}}{\mathrm{dt}} = C\cos(\theta_{k,B} - \theta_{j,B} - \varphi_1)$$

$$\frac{\mathrm{d}\theta_{j,B}}{\mathrm{dt}} = C\frac{1}{A_{j,B}}\cos(\theta_{k,B} - \theta_{j,B} - \varphi_2)$$

Where $C = \sqrt{a^2 + b^2}$, $\varphi_1 = \arctan\left(\frac{-b}{a}\right)$, $\varphi_2 = \arctan\left(\frac{a}{b}\right)$

The steps for the non-amplitude normalized functions are the same as above but without a divisor of $A_{k,B}$ resulting in coupling equations of the form:

$$\frac{dA_{j,B}}{dt} = CA_{k,B}cos(\theta_{k,B} - \theta_{j,B} - \varphi_1)$$

$$\frac{d\theta_{j,B}}{dt} = C\frac{A_{k,B}}{A_{j,B}}cos(\theta_{k,B} - \theta_{j,B} - \varphi_2)$$

We note that the above functions exhibit phase dependent and amplitude dependent interaction terms. Notably the phase difference coupling portion of the terms is similar to those used in, [38] and by extension the Kuramoto model of phase coupled oscillators. Below we list all of the above coupling functions. We observe that while the majority of each function is dominated by those of the form described above with a proportionally small remainder of the form (E+Fi)*Re(z). By truncating the coefficients corresponding to the (E+Fi)*Re(z) remainder components and keeping only the (A+Bi)*z components we found they did not impact on model performance (0.19 vs 0.19).The following coefficients are divided by a normalizing scale factor of 230 to put them on a regular scale across terms.

		Target j, Source k, band B<br>$dx_{(j,B)}/dt = a\,x_{k,B} + b\,y_{k,B}$		Target j, Source k, band B<br>$dy_{(j,B)}/dt = bx_{k,B} + a\,y_{k,B}$		
Connection	(j, B),<br>(k, B),	$ax_{k,B}$	$b\,y_{k,B}$	$b\,x_{k,B}$	$ay_{k,B}$	**Simplified Function** and remainder<br>$dz_{(j,B)}/dt=$
MotR to MotL Band 1	(1,1),<br>(2,1)	0.30	-2.27	2.29	0.33	**(0.33 + 2.27 i) $z_{2,1}$** - (0.03 - 0.02 i) $Re(z_{2,1})$
MotR to MotL Band 2	(1,2),<br>(2,2)	0.60	-4.48	4.01	0.48	**(0.48 + 4.48 i) $z_{2,2}$** + (0.12 - 0.47 i) $Re(z_{2,2})$
MotR to MotL Band 3	(1,3),<br>(2,3)	0.43	-5.82	5.82	0.43	**(0.43 + 5.82 i) $z_{2,3}$**

bioRxiv preprint doi: https://doi.org/10.1101/2025.09.23.678032; this version posted September 16, 2026. The copyright holder for this preprint (which was not certified by peer review) is the author/funder, who has granted bioRxiv a license to display the preprint in perpetuity. It is made available under a CC-BY 4.0 International license.

SomL to MotL Band 1	(1,1), (3,1)	535.91	0.00	0.00	466.65	**(466.65 i) $z_{3,1}$** + (69.26) Re($z_{3,1}$)
SomL to MotL Band 2	(1,2), (3,2)	1180.15	-249.13	249.49	1227.90	**(1227.9 + 249.13 i) $z_{3,2}$** + (47.75 - 0.36 i) Re($z_{3,2}$)
SomL to MotL Band 3	(1,3), (3,3)	663.82	66.96	-82.65	674.31	**(674.31 - 66.96 i) $z_{3,3}$** - (10.49 + 15.69 i) Re($z_{3,3}$)
MotL to MotR Band 1	(2,1), (1,1)	-0.11	-1.13	1.13	-0.12	**(-0.12 + 1.13 i) $z_{1,1}$** + (0.01) Re($z_{1,1}$)
MotL to MotR Band 2	(2,2), (1,2)	-0.21	-2.78	2.12	-0.19	**(-0.19 +2.78 i) $z_{1,2}$** - (0.02 + 0.66 i) Re($z_{1,2}$)
MotL to MotR Band 3	(2,3), (1,3)	-0.39	-2.94	2.92	-0.40	**(-0.04 + 2.94 i) $z_{1,3}$** + (0.01 - 0.02 i) Re($z_{1,3}$)
SomR to MotR Band 1	(2,1), (4,1)	673.14	-173.27	158.65	678.86	**(678.86 + 173.27i) $z_{4,1}$** - (5.72 + 14.62 i) Re($z_{4,1}$)
SomR to MotR Band 2	(2,2), (4,2)	1033.77	-498.02	469.17	1060.63	**(1060.63 + 498.02i) $z_{4,2}$** - (26.86 + 28.85 i) Re($z_{4,2}$)
SomR to MotR Band 3	(2,3), (4,3)	462.28	-228.30	230.57	471.37	**(471.37 + 228.3i) $z_{4,3}$** - (9.09 - 2.27i) Re($z_{4,3}$)
SomR to SomL Band 1	(3,1), (4,1)	0.00	-392.34	405.60	0.00	**(392.34 i) $z_{4,1}$** + (13.26 i) Re($z_{4,1}$)
SomR to SomL Band 2	(3,2), (4,2)	-0.28	-556.35	542.84	0.00	**(556.35i) $z_{4,2}$** - (0.28 + 13.51i) Re($z_{4,2}$)
SomR to SomL Band 3	(3,3), (4,3)	0.00	-193.35	194.25	0.00	**(193.35 i) $z_{4,3}$**+ (0.9 i) Re($z_{4,3}$)
MotL to SomL Band 1	(3,1), (1,1)	7.06	0.00	4.38	0.00	(7.06 + 4.38 i)Re($z_{1,1}$)
MotL to SomL Band 2	(3,2), (1,2)	-157.61	-310.06	300.40	-165.01	**(-165.01 + 310.06 i) $z_{3,2}$** + (7.4 - 9.66 i) Re($z_{1,2}$)
MotL to SomL Band 3	(3,3), (1,3)	-560.64	-54.93	79.79	-546.35	**(-546.35 + 54.93 i) $z_{1,3}$** - (14.29 - 24.86 i) Re($z_{1,3}$)

bioRxiv preprint doi: https://doi.org/10.1101/2025.09.23.678032; this version posted September 16, 2026. The copyright holder for this preprint (which was not certified by peer review) is the author/funder, who has granted bioRxiv a license to display the preprint in perpetuity. It is made available under a CC-BY 4.0 International license.

SomL to SomR Band 1	(4,1), (3,1)	247.21	683.97	-694.81	244.44	**(244.44 - 683.97 i) $z_{3,1}$** +(2.77 - 10.84 i) Re($z_{3,1}$)
SomLtoSomR Band 2	(4,2), (3,2)	744.39	-74.43	62.24	745.77	**(745.77 + 74.43 i) $z_{3,2}$** - (1.38 + 12.19 i) Re($z_{3,2}$)
SomL to SomR Band 3	(4,3), (3,3)	452.84	-100.43	100.71	463.17	**(463.17 + 100.43 i) $z_{3,3}$** - (10.33 - 0.28 i) Re($z_{3,3}$)
MotR to SomR Band 1	(4,1), (2,1)	-84.89	67.29	-73.58	-101.82	**(-101.82 - 67.29 i) $z_{2,1}$** + (16.93 - 6.29 i) Re($z_{2,1}$)
MotR to SomR Band 2	(4,2), (2,2)	-349.46	-238.50	239.02	-322.23	**(-322.23 + 238.5 i) $z_{2,2}$** - (27.23 - 0.52 i) Re($z_{2,2}$)
MotR to SomR Band 3	(4,3), (2,3)	-464.81	-143.46	143.48	-442.28	**(-442.28 + 143.46 i) $z_{2,3}$**-(22.53 - 0.02 i) Re($z_{2,3}$)

Supp. Table 5. Cross regional coupling functions in the model from cross frequency library 1. Bold indicates simplified function in z.

The remainder of selected functions are shown graphically in Fig. S4E. We note that the remaining functions contribute little to overall model performance or modulation of seizures (Fig. S4F and G). All coefficients with library selection steps are available in the accompanying MATLAB code available at https://github.com/hulljm/EmergentSeizures.
